# VCPIP1 facilitates pancreatic adenocarcinoma progression via Hippo/YAP signaling

**DOI:** 10.1038/s41419-025-07746-2

**Published:** 2025-05-28

**Authors:** Zhihao Liu, Chenmiao Zhang, Yingwen Gai, Peng Su, Beibei Wang, Peng Liu, Limin Wang, Yue Lin, Jian Zhu, Xiaodong Tan

**Affiliations:** 1 Department of General Surgery, Shengjing Hospital of China Medical University, Shenyang, 110000 China; 2 Xinxiang Key Laboratory of Tumor Migration and Invasion Precision Medicine, School of Medical Technology, Xinxiang Medical University, Xinxiang, 453003 Henan PR China; 3 Department of Pathology, Qilu Hospital, Cheeloo College of Medicine, Shandong University, Jinan, 250033 Shandong PR China; 4 Laboratory of Medical Genetics, Harbin Medical University, Harbin, 150081 Heilongjiang PR China; 5 Department of General Surgery, Tiemei General Hospital of Liaoning Province Health Industry Group, Tieling, 112000 Liaoning PR China

**Keywords:** Oncogenes, Deubiquitylating enzymes

## Abstract

Dysregulation of Hippo signaling is observed in pancreatic adenocarcinoma (PAAD). Moreover, overactivation of YAP is crucial for tumor progression. Although the inhibitory phospho-cascade is functional, the reason for YAP hyperactivation in PAAD remains unclear. Recent studies have revealed that the ubiquitin modification of YAP also plays an important role in the Hippo/YAP axis and cancer progression. To gain a better understanding of the potential mechanisms underlying the ubiquitination and deubiquitination of YAP, we carried out siRNA screening for critical deubiquitinases in PAAD. By using a deubiquitinase (DUB) library, we identified valosin-containing protein-interacting protein 1 (VCPIP1) as an important effector of YAP function and PAAD progression. Inhibition of VCPIP1 hampered PAAD progression via Hippo signaling. Clinical data revealed that VCPIP1 was elevated in PAAD and correlated with poor survival in PAAD patients. Biochemical assays demonstrated that VCPIP1 interacted with YAP, inhibiting K48-linked polyubiquitination and thereby increasing YAP stability. YAP directly binds to the VCPIP1 promoter region, enhancing its transcription in PAAD. Our study revealed a forward feedback loop between VCPIP1 and Hippo signaling in PAAD, indicating that VCPIP1 is a potential therapeutic drug target in PAAD.

## Introduction

Pancreatic cancer is among the deadliest human malignancies globally, with a median survival of approximately six months [1]. Pancreatic adenocarcinoma (PAAD) accounts for 90% of all cases, whereas current chemotherapy or targeted therapy has limited effects on survival [2]. Therefore, it is crucial to clarify signaling networks and identify new therapeutic targets for PAAD patients. Recent studies revealed that hyperactivation of the Hippo/YAP axis was frequently observed in PAAD samples, but further exploration of the function of YAP and its regulatory mechanisms is highly important for the treatment of PAAD [3].

Hippo signaling [4–6], which is an evolutionarily conserved pathway in mammals, was first discovered in Drosophila. Hippo signaling is a self-regulating pathway crucial for controlling organ size, regenerating tissue, and maintaining homeostasis [7]. The core of Hippo signaling comprises a series of phosphorylation kinases [8], including Set20-like kinase 1/2 (MST1/2), large tumor suppressor 1/2 (LATS1/2) and the transcriptional cofactors YAP and TAZ. When Hippo signaling is activated, MST1/2 promotes the phosphorylation of LATS1/2 [9], which subsequently leads to the phosphorylation of YAP/TAZ [10]. Phosphorylated YAP/TAZ can be sequestered in the cytosol and degraded by a group of ubiquitin ligases [11, 12]. When Hippo signaling is turned off, unphosphorylated YAP/TAZ can enter the nucleus and coactivate transcription factors such as TEADs, triggering various transcriptional programs [13, 14].

Hippo/YAP signaling has been found to be involved in various types of malignancies [15–18]. An increasing number of studies have revealed that perturbation of the Hippo pathway can also trigger tumorigenesis in PAAD [19–21]. For example, YAP is overexpressed in PAAD samples, and the activation of YAP can facilitate the expression of a set of genes involved in cell proliferation and antiapoptotic effects in cell lines [3]. As the most important effector of the Hippo pathway, YAP can also synergize with AP-1 to initiate carcinogenic processes in pancreatic ductal cells [19], whereas YAP deletion can hamper the progression of PAAD in *Kras*-mutant mice [21, 22]. However, why YAP is hyperactivated while the inhibitory kinase cascade, such as MST1/2 and LATS1/2, is still functional is unknown [23]. Recent research has indicated that posttranslational modifications, including ubiquitination, can regulate the function of YAP in PAAD tumorigenesis [24].

The equilibrium between E3 ubiquitin ligases and deubiquitinases regulates the stability of both the YAP protein and its transcriptome [25, 26]. Bioinformatics analysis of PAAD samples and DUB siRNA screening identified VCPIP1 as a key regulator of YAP activity and PAAD progression. Inhibiting VCPIP1 may be an effective approach for targeting Hippo-driven PAAD.

## Materials and methods

### Reagents

In this study, the reagents CAS-12290-201 (HY-159000), XMU-MP-1 (HY-100526), verteporfin (HY-B0146), CHX (HY-12320), and MG132 (HY-13259) were obtained from MedChemExpress (MCE).

### Cell culture

The AsPC-1, HEK-293T and PANC-1 cells were sourced from the American Type Culture Collection (ATCC). RPMI-1640 (PM150110B, Procell) was used for AsPC-1 cells, and DMEM (PM150210B, Procell) was used for HEK-293T and PANC-1 cells. The incubation conditions were set at 37 °C with a 5% CO_2_ atmosphere.

HEK293T cells were subjected to transduction with shRNA lentiviral particles to generate lentiviruses that target VCPIP1 for depletion. After the envelope plasmids psPAX2 and pMD2G were transfected into HEK-293T cells, the cells were incubated for two days. After that, the lentivirus was obtained. Subsequently, PANC-1 cells were treated with antibiotic-free medium containing lentivirus. Puromycin (2 μg/mL) was applied to sort the infected cells to generate stable cell lines.

### DUBs screening

The TCGA-PAAD data were obtained from the Genomic Data Commons. Patients in the cohort were evenly divided into two groups according to the expression level of deubiquitinating enzyme-encoding genes. Gene set enrichment analysis (GSEA) was performed with GSEA software version 4.3.3, and the normalized enrichment score (NES) in the ‘CORDENONSI_YAP_CONSERVED_SIGNATURE’ gene set was recorded. DUBs with NESs >1.5 were subjected to further siRNA screening; their siRNAs were transfected into PANC-1 cells for 48 h, after which the cells were harvested and subjected to qRT‒PCR analysis. The mRNA level of expression of CTGF was examined, and DUBs were ranked according to the CTGF expression level.

### Mouse xenograft tumor model

The mice were purchased from SPF Biotechnology (Beijing, China). Six-week-old female nude mice were assigned randomly to different groups, with 6 mice in each group. For the in vivo experiments, the mice were injected subcutaneously with 3 × 10^6^ PANC-1 cells suspended in 150 μL of PBS. The tumor-bearing mice were observed continuously for six weeks, and the tumor volume was recorded weekly. Volume = length × width^2^/2.

### Plasmids and siRNA

The Flag-VCPIP1, Flag-VCPIP1^C219A^ and VCPIP1 truncations were generated via PCR and then inserted into the pcDNA 3.1 vector. The HA-Ub, HA-K48-Ub, HA-K48R-Ub, HA-K63-Ub, HA-K63R-Ub, Myc-YAP and its mutant plasmids were stored in our laboratory and have been described in our previous works [27]. For plasmid transfection, Lipofectamine 2000 (1662298, Invitrogen) was utilized. Moreover, for siRNA transfection, RNA iMAX reagent (13778150, Invitrogen) was used. The following sequences of the VCPIP1 siRNAs were used: siVCPIP1#1: CAG GCA GCU UGA UCC UGA UUU GGU U; siVCPIP1#2: GAG GUG ACA GAA GUU UGC AAG AUA A; siControl: UUC UCC GAA CGU GUC ACG U; siYAP#1: GUC UCA GGA AUU GAG AAC A; siYAP#2: GUC AGA GAU ACU UCU UAA A.

### Luciferase reporter assay

A luciferase reporter plasmid containing the TEAD response element was used for this assay. The cells transfected with siVCPIP1 or siControl, the reporter plasmid and Renila were incubated for 24 hours and then collected and subjected to a luciferase assay via the Dual-Luciferase^®^ Reporter Assay System (E1090, Promega).

### RNA extraction and real-time PCR

An RNA isolator (R401, Vazyme) was used for total RNA extraction. cDNA was subsequently reverse transcribed from the RNA. Then, amplification was carried out via the indicated primers and SYBR GREEN (Q711-02, Vazyme) on a LightCycler®480 (Roche). 36B4 was used as an internal reference. The primers used were as follows: 36B4: forward: AGA CCT TCT TCC AGG CTT TG; reverse: GCT CCC ACC TTG TCT CCA GTC. VCPIP1: Forward: GCG CCT TCC TCA TAG AAC CA; Reverse: ATT GGC CCG CTT AAT GTC CT. CYR61: Forward: GGT CAA AGT TAC CGG GCA GT; Reverse: GGA GGC ATC GAA TCC CAG C. CTGF: Forward: CAG CAT GGA CGT TCG TCT G; Reverse: AAC CAC GGT TTG GTC CTT GG. YAP1: Forward: GAA CTC GGC TTC AGG TCC TC; Reverse: GGT TCA TGG CAA AAC GAG GG. TAZ: Forward: GAT GTG AGA GCC GAA GCC C; Reverse: CGG ATT CAT CTT CTG GGC GG.

### Western blotting

The reagents used for western blotting were as follows: RIPA buffer (SW104-01, Seven), protease inhibitor cocktail (P1045, Beyotime), enhanced chemiluminescence (ECL) luminescence reagent (MA0186, Meilunbio), and PVDF membranes (IPVH00010, Merck Millipore). The following antibodies were used: anti-VCPIP1 (88153S, CST; sc-515291, Santa Cruz), anti-Flag (20543-1-AP, Proteintech), anti-β-Actin (4967S, CST), anti-Myc (16286-1-AP, Proteintech), anti-YAP (14074S, CST), anti-Phospho-YAP (Ser127) antibody (13008S, CST), anti-TAZ antibody (83669S, CST) and anti-HA (90513, Biolegend). The secondary antibodies used were goat anti-rabbit IgG (A0208, Beyotime) or goat anti-mouse IgG (A0216, Beyotime). Protein samples were extracted via RIPA buffer with a protease cocktail, followed by electrophoresis and transfer to a PVDF membrane. The membrane was subsequently blocked with 5% milk and incubated with primary and secondary antibodies in sequence. Luminescence was detected via an enhanced chemiluminescence (ECL) system (Bio-Rad ChemiDoc).

### CCK8 assay

After the indicated transfection or treatment, the cells were seeded in 96-well plates at 2000 cells per well. After cell adhesion, 10 μl of CCK8 reagent (Beyotime, C0038) was added to the wells at the indicated time points for 5 consecutive days, and the OD450 was measured after a 2-hour incubation at 37 °C with 5% CO_2_. The first time point was determined as 0 h, and proliferation rates were calculated for each time point.

### IC50 determination and synergy score calculation

For IC50 determination, AsPC-1 or PANC-1 cells were pretreated with CAS-12290-201 (4 μM) for 48 h and then treated with the indicated concentrations of gemcitabine. After 24 h of gemcitabine treatment, cell viability was measured via the CCK8 reagent.

For synergy score calculation, CAS-12290-201-pretreated cells were treated with the indicated concentrations of gemcitabine, and then, cell viability was measured via a CCK8 assay. The experiments were repeated 3 times, and the average cell viability at each concentration gradient was uploaded to the SynergyFinder website (synergyfinder.org) for the calculation of the synergy score [28].

### Transwell assay

After the indicated transfection or treatment, the cells were starved in serum-free medium for 12 h and then collected and resuspended in serum-free medium. The cells were diluted to 5 × 10^5^/ml and seeded into transwell chambers (#3422, Corning) with 100 µl of cells in each chamber. To exclude proliferation-induced confounding effects, serum-free medium was maintained in the upper chamber throughout the Transwell assay. Five hundred microlitres of medium supplemented with 20% FBS was added to the lower chamber. For invasion experiments, 100 μL of matrix gel was added to each chamber before the cells were seeded. The cells were incubated for 12 h, fixed with 4% paraformaldehyde and stained with crystal violet.

### Wound healing assay

After the indicated transfection or treatment, the cells were seeded in 6-well plates at 5 × 10^5^ cells per well. After the cells grew to confluence, a scratch wound was made on the monolayer of cells via a sterile tip. Images of the gap between the two edges of the scratch were captured via an EVOS microscope immediately after scratching and 24 h after scratching. The images were subsequently processed via ImageJ to calculate the migration gap area. Migration gap area = (0 h gap area – 24 h gap area)/0 h gap area.

### Apoptosis analysis

After the indicated transfection or treatment, the cells were collected and incubated with an apoptosis detection reagent (556547, BD Biosciences) for 15 minutes in the dark and subsequently analyzed via flow cytometry (BD Biosciences).

A Caspase-3 Assay Kit (C1168S, Beyotime) was used to detect caspase-3/7 activity. After the indicated treatments, the cells were incubated with caspase-3/7 substrate for 1 hour, after which images were captured with an EVOS microscope.

### Immunofluorescence assay

The cells were fixed with 4% paraformaldehyde and permeabilized with 0.2% Triton X-100. Then, the cells were blocked with 5% BSA (ST025, Beyotime) for 1 hour. The cells were subsequently incubated with an anti-VCPIP1 antibody (mouse, sc-515291; Santa Cruz) and an anti-YAP antibody (rabbit, 14074S; CST) at 4 °C overnight. Then, the cells were incubated with secondary antibodies and stained with DAPI (C1005, Beyotime). The secondary antibodies used in this study were as follows: CoraLite488-conjugated goat anti-rabbit (SA00013-2, Proteintech) for the rabbit antibody and CoraLite594-conjugated goat anti-mouse (SA00013-3, Proteintech) for the mouse antibody. A confocal laser scanner was used to capture the images.

### Coimmunoprecipitation assay

The cells were lysed (P0013, Beyotime) and centrifuged to obtain the supernatant. The supernatant was divided into IP and IgG groups and incubated with the indicated antibody or IgG and protein A/G (sc-2003, Santa Cruz) at 4 °C overnight. On the following day, the protein A/G beads were centrifuged and washed five times with lysis buffer. Then, 2x SDS‒PAGE loading buffer was added, and the mixture was denatured at 99 °C for 10 minutes and subjected to immunoblotting.

### Ubiquitination assay

The cells were treated with 20 μM MG132 for 6 h. The cells were subsequently harvested and precleaned with protein A/G agarose for 2 h to prevent nonspecific binding. The extract was subsequently incubated with anti-YAP or anti-Myc antibodies at 4 °C overnight. Finally, the precipitate was washed and denatured as described above, and the ubiquitination of the YAP protein was evaluated by immunoblotting with an anti-HA antibody.

### Mass spectrometry

The SDS‒PAGE gels were stained with Coomassie brilliant blue, and the protein band close to the target was excised. The protein within the gel was then digested overnight with 50 mM NH_4_HCO_3_ containing 2 μg of trypsin at 37 °C. The polypeptide was extracted from the gel via a solution containing 50% acetonitrile and 0.1% formic acid following digestion. The peptides were dried via a speed vacuum concentrator and reconstituted in 0.1% formic acid for LC‒MS/MS analysis with an EASY nLC‒1200 system (Thermo Scientific).

### Molecular docking analysis

Amber 22 (San Francisco, CA, USA) was employed to perform the molecular simulations. The ff19SB force field [29] was employed to calculate the system force field parameters. The TIP3P water model was utilized for solvation, and counterions were added to neutralize the system. The results of the molecular docking simulations were visualized via PyMOL.

### Chromatin immunoprecipitation

Chromatin immunoprecipitation was performed via a ChIP assay kit (P2078, Beyotime) following the manufacturer’s instructions. The antibody used in the ChIP experiment was an anti-YAP antibody (14074S, CST). The enriched DNA was purified with a DNA extraction kit (28106, Qiagen). The primer sequences for VCPIP1 used in ChIP‒qPCR were as follows: forward: ATA GCT CCT GGC TCT CGT GT; reverse: AAA GGA AAG TGC TTC GCC CT.

### Immunohistochemistry (IHC)

The tissue was fixed with 4% PFA and embedded in paraffin wax. The tissue was subsequently sectioned with a S700 paraffin microtome (RWD, USA) and stained with an immunohistochemical kit (PV-9000, ZSBG-BIO). The primary antibody was incubated at 4 °C overnight, and then the secondary antibody was incubated at room temperature for 2 h. The immune complexes were visualized with DAB chromogenic solution, and the nuclei were restained with hematoxylin. The images were captured via a digital pathology scanner. The antibodies used in the IHC experiments were as follows: anti-YAP (14074S, CST), anti-VCPIP1 (sc-515291, Santa Cruz), and anti-Ki-67 (27309-1-AP, Proteintech).

### Clinical samples

Paraffin-embedded tissue blocks were obtained from the Pathology Department of Shengjing Hospital. A total of 52 normal pancreatic tissues and 57 pancreatic cancer tissues were collected to detect the expression of VCPIP1 and YAP. The clinical data of 57 pancreatic cancer patients were subsequently retrieved for clinical relevance analysis. The personal information of the patients was well preserved, and no extra troubles were introduced to the patients.

### Analysis of publicly available clinical data

Gene expression data from GETx and TCGA-PAAD were obtained via UCSC Xena (xena.ucsc.edu). The expression levels of VCPIP1 were determined and visualized via GraphPad Prism 9. Kaplan‒Meier survival analysis was performed via GEPIA using TCGA-PAAD data.

### Transcriptome sequencing and data analysis

Three biological replicates of AsPC-1 cells transfected with siControl or siVCPIP1 were collected, and their RNA was extracted. The samples were sent to BGI (Shenzhen, China) for RNA sequencing analysis. Differentially expressed genes were identified via DESeq2, with significance defined as log2FC > 1 and p < 0.05. Volcanoplot and dotplot data were plotted via the online platform SRplot [30]. The data are available in the GEO database with the series number GSE284347.

### Data statistics

SPSS 25.0 was used for data analysis. All the data are presented as the means ± standard deviations from a minimum of three biological replicates, with at least six replicates used for the animal studies. Student’s t test was used to assess the differences between the two groups of continuous data. Chi-square test was used to assess the differences between the two groups of categorical data. Two-way ANOVA was used to assess differences between two groups across various time intervals. The variable Hill slope model of nonlinear regression was employed to estimate the IC50. A *p* value < 0.05 was considered statistically significant.

## Results

### VCPIP1 is a critical regulator of the Hippo pathway in PAAD

**Fig. 1:**
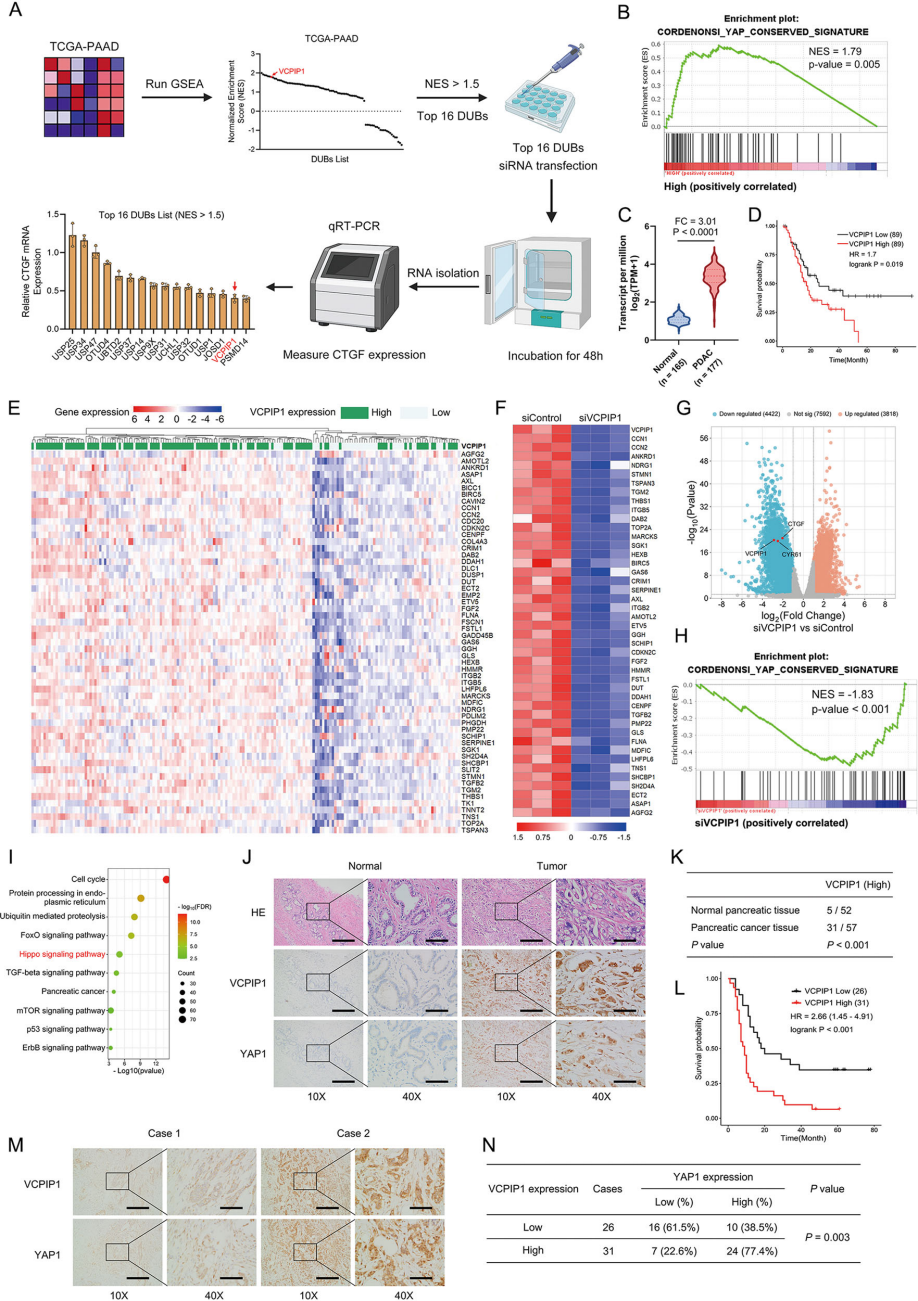
VCPIP1 is a critical regulator of the Hippo pathway in PAAD. **A** Illustration of deubiquitinase screening. Data from the TCGA-PAAD cohort were subjected to GSEA. Deubiquitinases were sorted by their normalized enrichment score (NES), and VCPIP1 ranked at the top of the list. The top 16 deubiquitinases with NESs >1.5 were further screened by transfecting their siRNAs (at a concentration of 50 nM) into PANC-1 cells. The expression of the YAP target gene CTGF was used as a readout of Hippo signaling activity. **B** GSEA of TCGA-PAAD data. High expression of VCPIP1 is positively correlated with the YAP gene set. **C** Data from GTEx and TCGA-PAAD were combined to analyze the expression of VCPIP1 in normal and PAAD tissues. Data were downloaded from UCSC Xena and normalized to enable comparability. **D** Kaplan‒Meier plot showing the relationship between VCPIP1 expression and disease-free survival in TCGA-PAAD patients. **E** Heatmap showing the correlation between VCPIP1 expression and genes in the YAP signature. TCGA-PAAD data were utilized. **F** Heatmap showing that the expression of YAP signature genes was downregulated in the siVCPIP1 group. **G** Genes that were differentially expressed between the siVCPIP1 and siControl groups were visualized via a volcano plot. **H** GSEA was performed on our RNA-Seq data (siVCPIP1 vs siControl). The gene expression of the siVCPIP1 group was negatively correlated with the YAP gene set. **I** Enrichment analysis was performed on the genes with differential expression in our RNA-Seq data. The dot plot shows the top enriched KEGG pathways. **J**, **K** Immunohistochemistry (IHC) showing the expression of VCPIP1 and YAP in normal and pancreatic cancer tissues. VCPIP1 is significantly more highly expressed in pancreatic cancer samples than in normal pancreas samples. The scale bars are 400 μm for 10X and 100 μm for 40X. The P value was calculated via the chi-square test. **L** Kaplan‒Meier plot showing the relationship between VCPIP1 expression and overall survival in 57 pancreatic cancer patients in our hospital. **M**, **N** The correlation between VCPIP1 and YAP protein expression in pancreatic cancer patients was analyzed by immunohistochemistry. The scale bars are 400 μm for 10X and 100 μm for 40X. The P value was calculated via the chi-square test.

To identify important deubiquitinases in the Hippo pathway in PAAD, we carried out bioinformatics screening of the TCGA database, performed gene signature enrichment analysis (GSEA) of Hippo signaling and investigated the expression of each DUB in PAAD samples. The correlation scores were ranked via normalized enrichment scores (NESs) in PAAD samples (Fig. 1A). We further selected candidate DUBs (NES > 1.5; P < 0.05) that were significantly correlated with Hippo signaling activity in PAAD. The expression of these candidate DUBs was depleted via siRNA in PAAD cells, and the expression of CTGF was used as a readout for Hippo signaling activity, which revealed that VCPIP1 could be a critical regulator of Hippo signaling in PAAD (Fig. 1A). GSEA of data from the TCGA database revealed that the expression of VCPIP1 was positively correlated with Hippo/YAP function, which might indicate that VCPIP1 facilitates YAP function in PAAD (Fig. 1B). Next, we analyzed VCPIP1 expression using combined GETx and TCGA databases and found that the expression of VCPIP1 was elevated in PAAD (Fig. 1C) as well as in several other TCGA cancer types (Fig. S1A). Additionally, we found that high VCPIP1 expression is associated with poor prognosis not only in pancreatic cancer (PAAD) (Disease-free survival, Fig. 1D; Overall survival, Fig. S1B) but also in multiple other cancers (Fig. S1C–G), including lung adenocarcinoma (LUAD), kidney renal papillary cell carcinoma (KIRP), cervical squamous cell carcinoma (CESC), uterine corpus endometrial carcinoma (UCEC), and thyroid carcinoma (THCA). Moreover, the heatmap grouped by VCPIP1 from PAAD samples revealed a significant positive correlation between VCPIP1 and YAP target genes (Fig. 1E).

We further depleted VCPIP1 in PAAD cells for RNA sequencing analysis. Heatmap analysis revealed that a subset of YAP target genes was downregulated following VCPIP1 knockdown (Fig. 1F). A volcano plot revealed that CTGF and CYR61, which are classical YAP target genes, were significantly downregulated (Fig. 1G). GSEA indicated that the signature genes of YAP were generally reduced under VCPIP1 depletion conditions (Fig. 1H). Gene enrichment analysis revealed that VCPIP1 depletion affected the Hippo signaling pathway (Fig. 1I). Further immunohistochemistry validation using the same set of PAAD tissue samples revealed that the expression level of VCPIP1 was greater in PAAD than in normal pancreatic tissue (Fig. 1J, K) and that the expression of VCPIP1 was positively correlated with YAP expression (P = 0.003; Fig. 1M, N). Survival analysis of 57 PAAD patients in our department confirmed that VCPIP1 expression was correlated with poor survival (Fig. 1L). We further investigated the correlation between the expression of VCPIP1 and the clinical characteristics of PAAD samples. The findings revealed a positive correlation between VCPIP1 expression and both clinical stage and lymph node invasion (Supplementary Table 1).

### VCPIP1 is required for PAAD progression

**Fig. 2:**
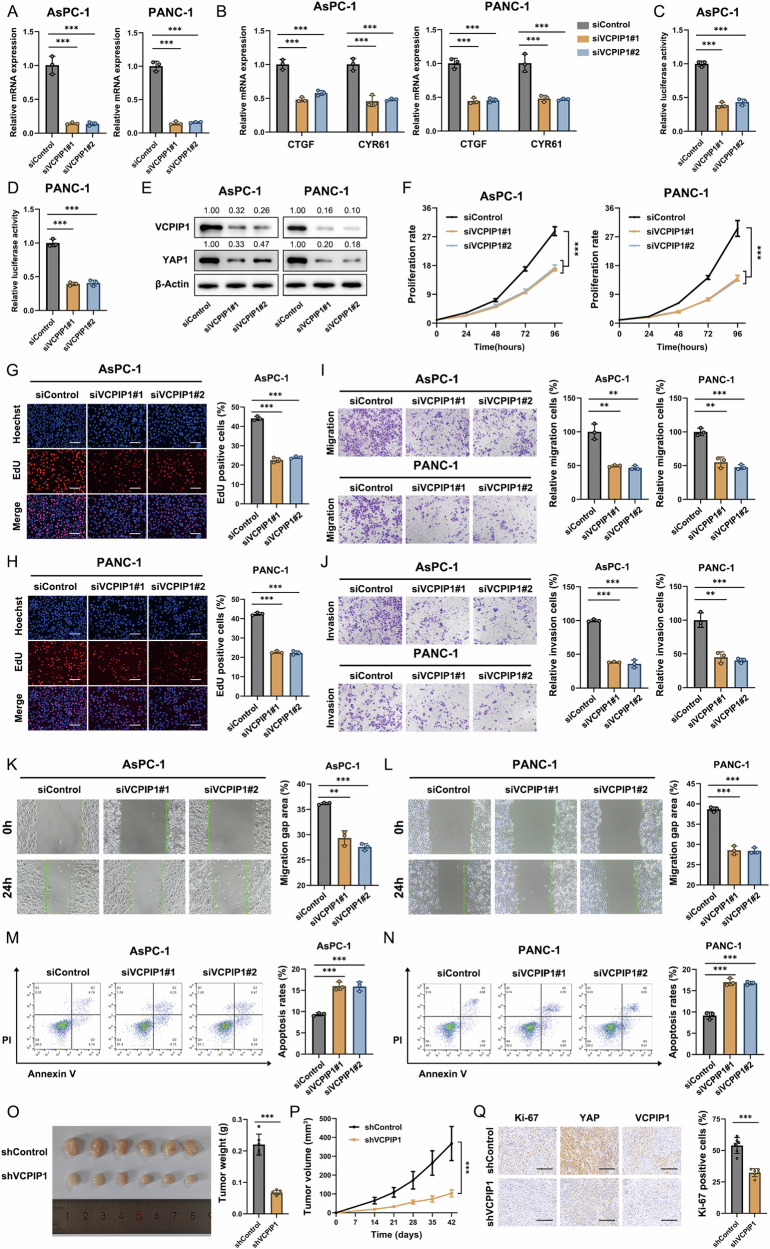
VCPIP1 is required for PAAD progression. **A** PAAD cells were transfected with either siControl or siVCPIP1#1/#2 at a concentration of 50 nM. The knockdown efficiency of VCPIP1 was subsequently measured via qRT‒PCR. **B** The mRNA expression levels of CTGF and CYR61 in PAAD cells after transfection with siControl or siVCPIP1 were measured via qRT‒PCR. **C**, **D** A luciferase reporter gene assay was used to detect TEAD transcriptional activity in AsPC-1 and PANC-1 cells after transfection with siControl or siVCPIP1. **E** The protein levels of VCPIP1 and YAP in PAAD cells after transfection with siControl or siVCPIP1 were measured via Western blotting. Proliferation rates of PAAD cells were evaluated by CCK-8 (**F**) and EdU (**G**, **H**) assays after transfection with siControl or siVCPIP1. Nuclei in the EdU assay were stained with Hoechst 33342. The scale bar is 200 μm. Transwell assays were performed to assess the migration (**I**) and invasion (**J**) abilities of PAAD cells after transfection with siControl or siVCPIP1. **K**, **L** A wound healing assay was performed to assess the migration ability of PAAD cells after transfection with siControl or siVCPIP1. **M**, **N** PAAD cell apoptosis was evaluated via flow cytometry after the cells were transfected with siControl or siVCPIP1 and then stained with PI and FITC. **O**–**Q** PANC-1 cells stably transfected with shControl or shVCPIP1 were inoculated subcutaneously into nude mice. Tumor weight (**O**) and volume (**P**) were evaluated. An IHC assay was used to evaluate Ki-67, YAP and VCPIP1 expression (**Q**). The percentage of Ki-67-positive cells was calculated. n = 6. The scale bar is 200 μm. The experiments were performed in triplicate. All the data are presented as the means ± SDs. Statistical methods: Student’s t test for (**A**–**D**, **G**–**O**, **Q**); two-way ANOVA for (**F**, **P**). *P < 0.05; **P < 0.01; ***P < 0.001.

We further explored the effect of VCPIP1 on PAAD progression by utilizing AsPC-1 and PANC-1 cells. The knockdown efficiency of VCPIP1 was determined in AsPC-1 and PANC-1 cells (Fig. 2A). The qRT‒PCR data revealed that VCPIP1 silencing inhibited the expression of CTGF and CYR61, which are classic YAP target genes, in both AsPC-1 and PANC-1 cells (Fig. 2B). A luciferase assay targeting the TEAD response element demonstrated that YAP activity was significantly inhibited by VCPIP1 depletion in both AsPC-1 and PANC-1 cells (Fig. 2C, D). The western blot data revealed that VCPIP1 depletion in AsPC-1 and PANC-1 cells decreased the protein level of YAP (Fig. 2E). The results of the CCK8 assay revealed that VCPIP1 depletion significantly repressed PAAD cell growth (Fig. 2F). In the EdU assay, VCPIP1 depletion led to a significant reduction in the number of proliferating cells (Fig. 2G, H). Transwell experiments demonstrated that VCPIP1 knockdown significantly impaired the migration and invasion abilities of PAAD cells (Fig. 2I, J). A wound-healing assay demonstrated that the migratory ability of cells decreased after VCPIP1 depletion (Fig. 2K, L). The results of the apoptotic assay revealed that VCPIP1 knockdown increased the proportion of apoptotic AsPC-1 and PANC-1 cells (Fig. 2M, N). A xenograft model in nude mice was used to evaluate the in vivo function of VCPIP1. The results revealed that the growth of xenograft tumors was significantly impeded after VCPIP1 knockdown (Fig. 2O, P; Fig. S2A–C). Ki67 staining of xenograft tumors revealed that the percentage of Ki67-positive cells was reduced when VCPIP1 was depleted (Fig. 2Q).

### Hydrolyzing activity is dispensable for the ability of VCPIP1 to regulate YAP function

**Fig. 3:**
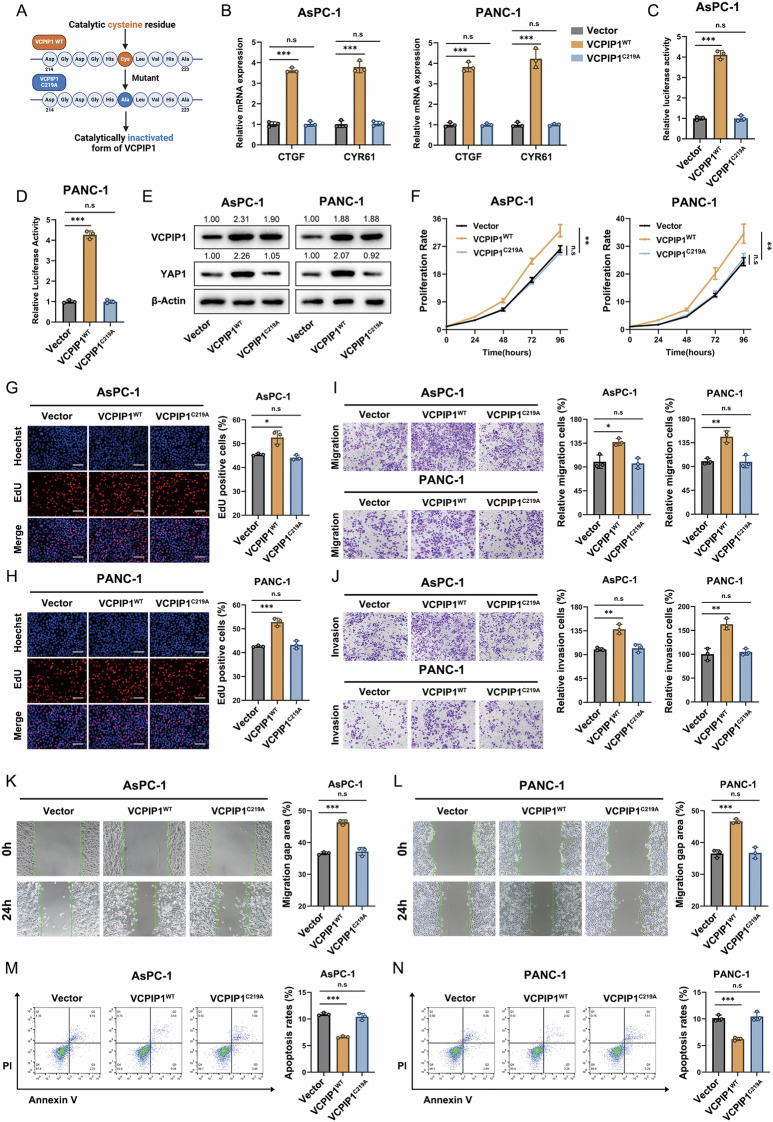
Hydrolysis activity is dispensable for the ability of VCPIP1 to regulate YAP function. **A** Schematic diagram of the C219A mutation of VCPIP1. **B** After the vectors or VCPIP1 WT or VCPIP1 C219A were transfected into PAAD cells, the mRNA expression levels of CTGF and CYR61 were measured via qRT‒PCR. A luciferase reporter gene assay was used to detect TEAD transcriptional activity in AsPC-1 (**C**) and PANC-1 (**D**) cells after transfection with the vector or with VCPIP1 WT or VCPIP1 C219A. **E** The protein levels of VCPIP1 and YAP in PAAD cells after transfection with vector, WT VCPIP1 or C219A VCPIP1 were measured via Western blotting. The proliferation rates of PAAD cells were evaluated by CCK-8 (**F**) and EdU (**G**, **H**) assays after the cells were transfected with the vector or with VCPIP1 WT or VCPIP1 C219A. For the EdU assay, the nuclei were stained with Hoechst 33342. The scale bar is 200 μm. Cell migration (**I**) and invasion (**J**) abilities were evaluated via Transwell assays in AsPC-1 and PANC-1 cells after transfection with vector or with VCPIP1 WT or VCPIP1 C219A. Cell migration ability was evaluated by a wound healing assay in AsPC-1 (**K**) and PANC-1 (**L**) cells after transfection with the vector or with VCPIP1 WT or VCPIP1 C219A. AsPC-1 (**M**) and PANC-1 (**N**) cells were transfected with vector, VCPIP1 WT or VCPIP1 C219A and stained with PI and FITC. Cell apoptosis was evaluated by flow cytometry. The experiments were performed in triplicate. All the data are presented as the means ± SDs. Statistical methods: Student’s t test for (**B**–**D**) and (**G**–**N**); two-way ANOVA for (**F**). *P < 0.05; **P < 0.01; ***P < 0.001.

Since VCPIP1 belongs to the deubiquitinase family, it is likely that VCPIP1 modulates YAP function through its deubiquitinase activity. Studies have shown that the 219th cysteine is the catalytic residue of VCPIP1 [31, 32]. To better understand the mechanisms involved, we aimed to explore the specific role of the catalytic activity of VCPIP1. Thus, we constructed the catalytically inactivated form of VCPIP1 (C219A) (Fig. 3A) and then tested its effect on PAAD cells. Consistently, the overexpression of wild-type VCPIP1 increased the expression of the classic YAP target genes CTGF and CYR61 in both AsPC-1 and PANC-1 cells, whereas the C219A mutant form of VCPIP1 did not have such effects (Fig. 3B). A luciferase assay of the TEAD response element revealed that VCPIP1 overexpression increased YAP activity in AsPC-1 and PANC-1 cells, whereas the mutant form of VCPIP1 did not have such effects (Fig. 3C, D). The western blot data revealed that VCPIP1 expression in AsPC-1 and PANC-1 cells increased the level of the YAP protein, whereas the mutant form of VCPIP1 did not have these effects (Fig. 3E). The results of the CCK8 assay revealed that VCPIP1 overexpression increased the growth of PAAD cells, whereas the mutant form of VCPIP1 did not (Fig. 3F). In the EdU incorporation assay, VCPIP1 overexpression dramatically increased the number of proliferating cells, whereas the mutant form of VCPIP1 did not have such effects (Fig. 3G, H). The transwell experiments demonstrated that VCPIP1 overexpression significantly increased the invasion and migration capacity of PAAD cells, whereas the mutant form of VCPIP1 did not have these effects (Fig. 3I, J). A wound-healing assay revealed that VCPIP1 overexpression enhanced PAAD cell migration, whereas the mutant form of VCPIP1 did not have such effects (Fig. 3K, L). The results of the apoptotic assay revealed that VCPIP1 overexpression inhibited the apoptosis of both AsPC-1 and PANC-1 cells, whereas the mutant form of VCPIP1 did not have such effects (Fig. 3M, N).

### YAP rescued the inhibitory effect of VCPIP1 knockdown on PAAD

**Fig. 4:**
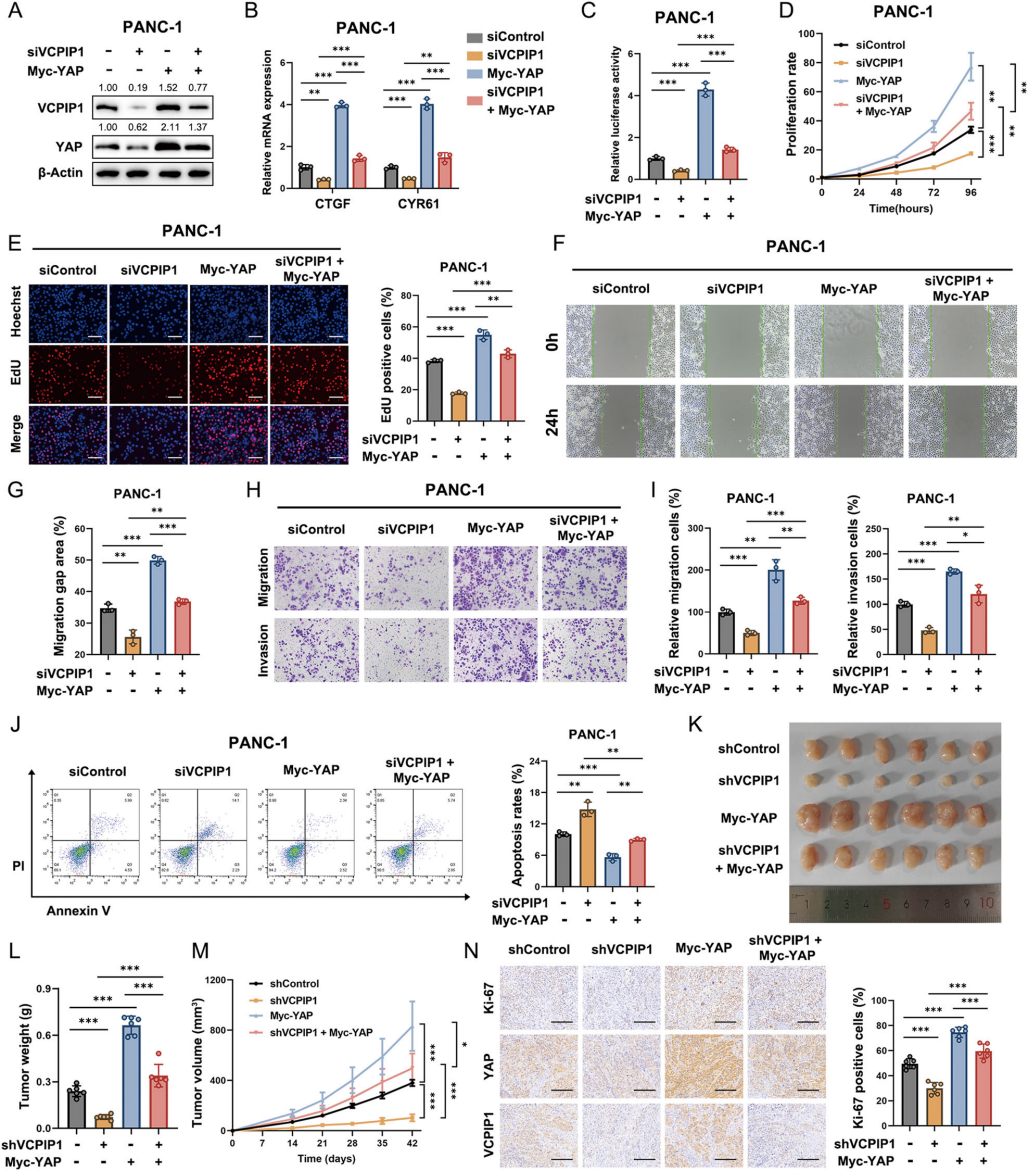
YAP rescued the inhibitory effect of VCPIP1 knockdown on PAAD. **A** PANC-1 cells were transfected with siControl + vector, siVCPIP1 + vector, siControl + YAP or siVCPIP1 + YAP. The protein levels of VCPIP1 and YAP were subsequently examined by immunoblotting. **B** The mRNA expression levels of CTGF and CYR61 in PANC-1 cells after transfection with the indicated siRNAs or plasmids were measured via qRT‒PCR. **C** TEAD transcriptional activity in PANC-1 cells after transfection with the indicated siRNA or plasmid was measured via a luciferase reporter gene assay. The proliferation rates of PANC-1 cells were evaluated by CCK-8 (**D**) and EdU (**E**) assays after transfection with the indicated siRNAs and plasmids. For the EdU assay, the nuclei were stained with Hoechst 33342. The scale bar is 200 μm. **F**, **G** Migration ability was evaluated by a wound healing assay in PANC-1 cells following transfection with the indicated siRNA or plasmid. **H**, **I** Cell migration and invasion abilities were evaluated via Transwell assays in PANC-1 cells following transfection with the indicated siRNAs and plasmids. **J** Apoptosis was evaluated by flow cytometry in PANC-1 cells after transfection with the indicated siRNA or plasmid. **K**–**N** PANC-1 cells stably transfected with the indicated lentivirus were inoculated subcutaneously into nude mice. Then, the tumor weight (**L**) and volume (**M**) were measured. Subsequently, an IHC assay was used to evaluate Ki-67, YAP and VCPIP1 expression (**N**). The percentage of Ki-67-positive cells was calculated. n = 6. The scale bar is 200 μm. The experiments were performed in triplicate. All the data are presented as the means ± SDs. Statistical methods: Student’s t test for (**B**, **C**, **E**‒**L**, **N**); two‒way ANOVA for (**D**, **M**). *P < 0.05; **P < 0.01; ***P < 0.001.

To verify whether the inhibitory effect of VCPIP1 knockdown depends on its regulation of the YAP protein, we conducted additional rescue experiments. We depleted VCPIP1 in PANC-1 cells and then further overexpressed YAP. Western blot analysis revealed that overexpression of YAP restored its protein level under VCPIP1 depletion conditions (Fig. 4A). The qRT‒PCR results demonstrated that depleting VCPIP1 suppressed the expression of YAP target genes, and this effect was reversed by subsequent YAP overexpression in PAAD cells (Fig. 4B). In the luciferase assay, VCPIP1 depletion decreased YAP activity in PAAD cells, whereas these effects were reversed by YAP overexpression in PAAD cells (Fig. 4C). VCPIP1 knockdown inhibited PAAD cell proliferation, which was restored by YAP overexpression, as demonstrated by the results of the CCK-8 (Fig. 4D) and EdU (Fig. 4E) assays. The migration rate of PAAD cells was also rescued by YAP overexpression under VCPIP1 depletion conditions, as shown by the results of the wound healing assay (Fig. 4F, G). We performed a transwell assay to assess the migration and invasion abilities of PAAD cells under VCPIP1 depletion and YAP overexpression conditions. The reduction in migration and invasion abilities due to VCPIP1 depletion was reversed by overexpressing YAP (Fig. 4H, I). An annexin V/PI staining apoptosis assay demonstrated that the percentage of apoptotic cells was elevated upon VCPIP1 depletion, whereas YAP overexpression alleviated the proapoptotic effects induced by VCPIP1 depletion (Fig. 4J). We utilized a xenograft mouse model to assess the in vivo impact of depleting VCPIP1 and overexpressing YAP. The results showed that the growth inhibitory effect caused by VCPIP1 depletion could be effectively rescued by YAP overexpression in PAAD cells (Fig. 4K–N; Fig. S2D). Ki67 staining indicated that the suppressed proliferation activity caused by VCPIP1 depletion could be restored through YAP overexpression (Fig. 4N).

### VCPIP1 interacts with YAP and regulates YAP protein stability

**Fig. 5:**
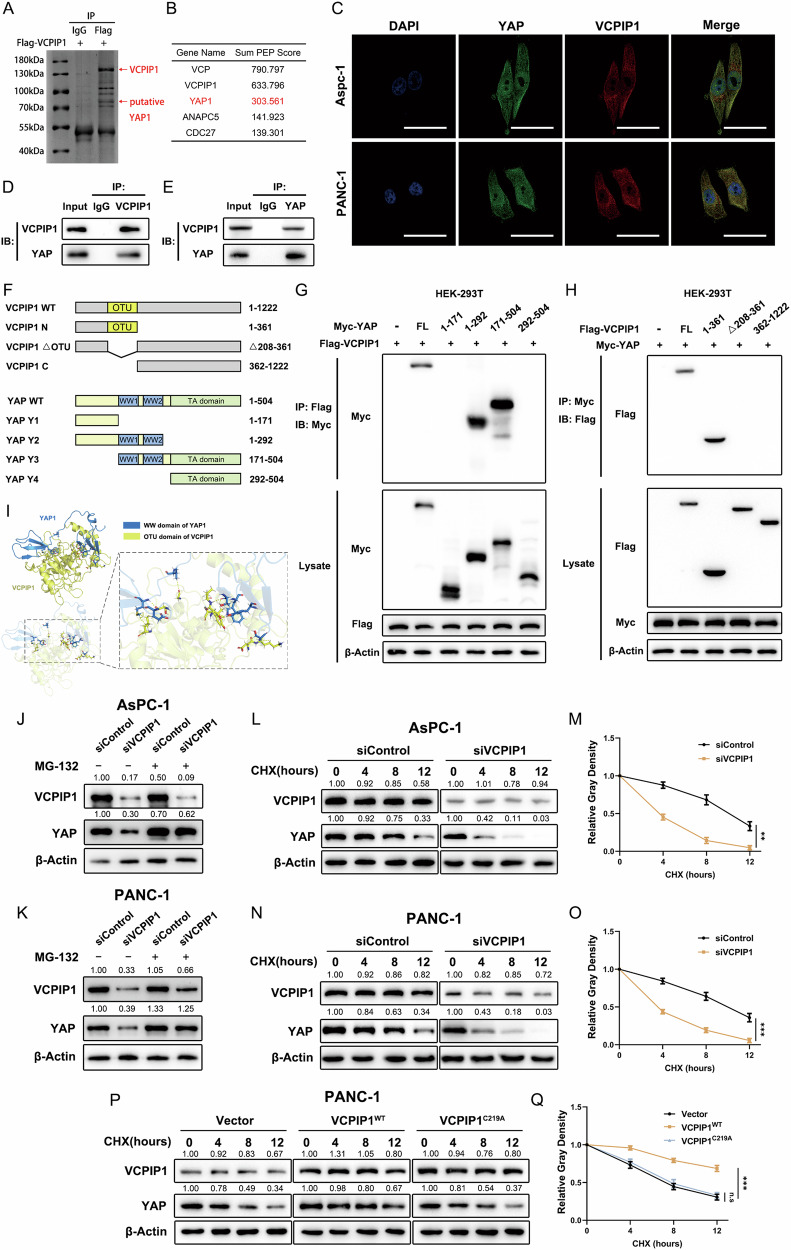
VCPIP1 interacts with YAP and regulates YAP protein stability. **A** PANC-1 cells transfected with Flag-VCPIP1 were lysed and immunoprecipitated with a Flag tag antibody or isotype IgG. Immunoprecipitated samples were denatured, subjected to electrophoresis and subsequently stained with Coomassie blue. The red arrow indicates the putative band of VCPIP1 and YAP. **B** Top 5 proteins enriched in the samples obtained via immunoprecipitation and then analyzed via protein mass spectrometry, as indicated in (**A**). **C** Immunofluorescence assays demonstrated the localization of the VCPIP1 and YAP proteins in AsPC-1 and PANC-1 cells. Green and red represent YAP and VCPIP1, respectively. Nuclei were stained with DAPI. The scale bar is 50 μm. **D**, **E** The interaction between endogenous VCPIP1 and YAP was detected through a co-IP assay. The lysates of PANC-1 cells were separately subjected to immunoprecipitation with anti-VCPIP1 (**D**) or anti-YAP1 (**E**) antibodies. Subsequently, western blotting was employed to analyze and detect the corresponding results. **F** The schematic illustrates wild-type and truncated VCPIP1 mutants (residues 1--361, Δ208--361, and 362--1222) as well as YAP and its truncated mutants (residues 1--171, 1--292, 171--504, and 292--504), which were used in the following Co-IP assay. Co-IP assays demonstrated that the WW domain of YAP (**G**) and the OTU domain of VCPIP1 (**H**) are required for their interaction. **I** The docking diagram, which depicts the interaction between the OTU domain of VCPIP1 and the WW domain of YAP, was visualized via PyMOL. MG132 abrogates the ability of siVCPIP1 to downregulate the protein level of YAP in AsPC-1 (**J**) and PANC-1 (**K**) cells. The cells were transfected with siControl or siVCPIP1 and then treated with 10 μM MG132 as indicated for 12 h. VCPIP1 and YAP were examined by immunoblotting at the protein level. Knockdown of VCPIP1 decreases YAP protein stability in AsPC-1 (**L**, **M**) and PANC-1 (**N**, **O**) cells. The cells were transfected with siControl or siVCPIP1 and then treated with CHX (100 μM) at different time points. **P**, **Q** VCPIP1 overexpression promotes YAP protein stability, whereas overexpression of the VCPIP1 C219A mutant has no significant effect on YAP protein stability. The CHX concentration was 100 μM. **P < 0.01; ***P < 0.001. Two-way ANOVA was employed for statistical analysis.

We further investigated the potential mechanism by which VCPIP1 affects YAP function. We overexpressed Flag-tagged VCPIP1 in PANC-1 cells and then performed immunoprecipitation with an anti-Flag antibody followed by mass spectrometry analysis. The results demonstrated that YAP was among the highest-ranked proteins (Fig. 5A, B). Immunofluorescence staining revealed that the YAP protein was present in both the cytosol and nucleus, whereas VCPIP1 was predominantly found in the cytosol (Fig. 5C). Co-IP assays revealed the endogenous interaction of VCPIP1 and YAP in PAAD cells (Fig. 5D, E). We further constructed truncated forms of VCPIP1 and YAP to assess the interactions among the domains of the two proteins (Fig. 5F). A Co-IP assay demonstrated that the interaction between VCPIP1 and YAP requires the OTU domain of VCPIP1 and the WW domain of YAP (Fig. 5G, H). This result was consistent with our molecular docking analysis, which predicted that the OTU domain of VCPIP1 and the WW domain of YAP are responsible for the VCPIP1-YAP interaction (Fig. 5I). We further used MG132, which is a potent inhibitor of the proteasome, to verify whether VCPIP1 increases YAP stability through the proteasome pathway. The results revealed that the protein level of YAP decreased after VCPIP1 depletion, whereas this effect was diminished after MG132 treatment (Fig. 5J, K). A protein stability assay using cycloheximide revealed that VCPIP1 depletion led to a reduction in YAP protein stability in AsPC-1 and PANC-1 cells (Fig. 5L–O). We further overexpressed both the wild-type form and the mutant form of VCPIP1 (C219A) to examine whether the ubiquitin catalytic function was essential for YAP stability. The results showed that VCPIP1 overexpression stabilized the YAP protein, but the mutant form of VCPIP1 did not (Fig. 5P, Q).

### VCPIP1 stabilizes YAP by reducing YAP K48-linked polyubiquitination

**Fig. 6:**
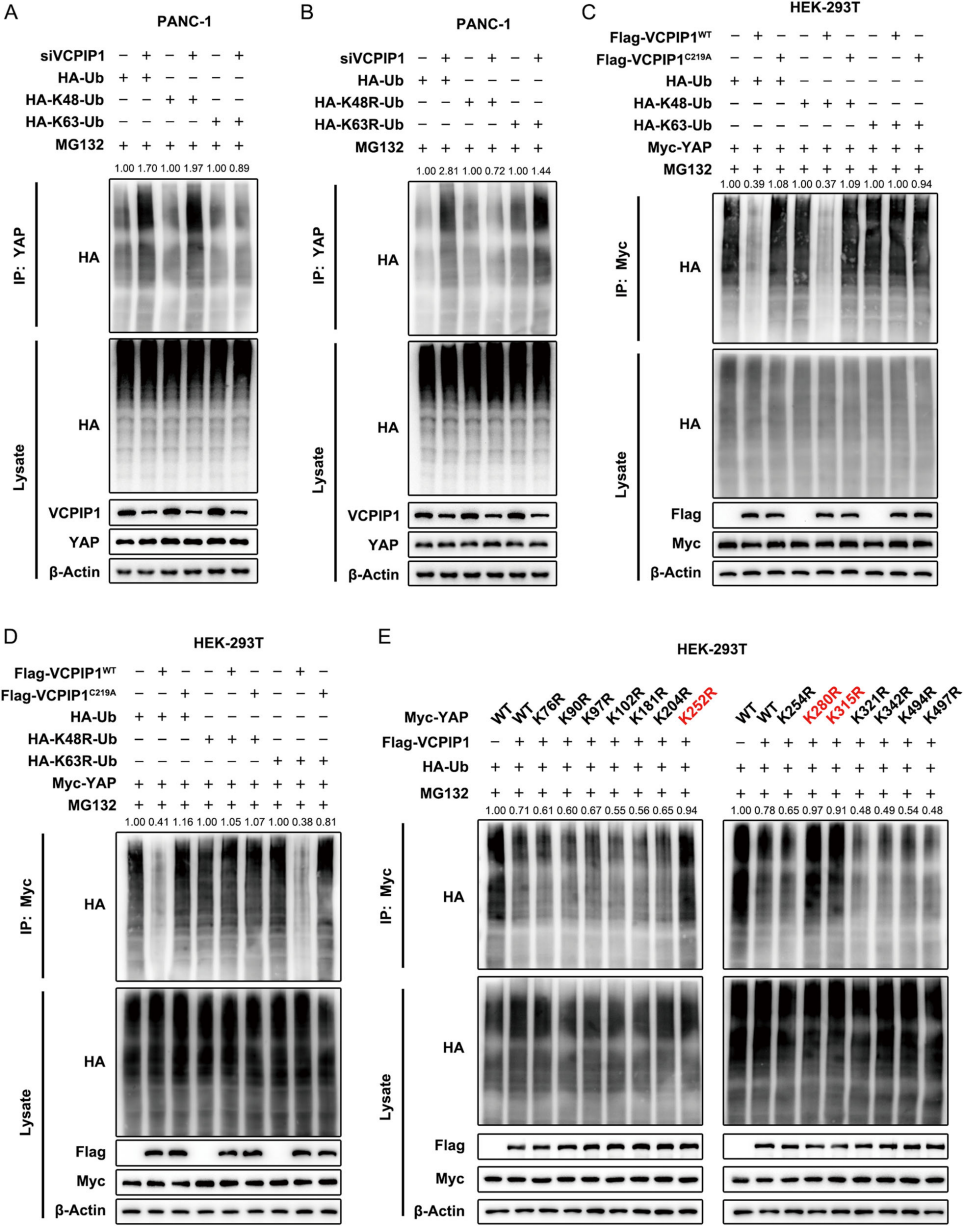
VCPIP1 stabilizes YAP by reducing YAP K48-linked polyubiquitination. **A**, **B** VCPIP1 knockdown increased K48-linked polyubiquitination of the YAP protein but did not affect K63-linked polyubiquitination of the YAP protein. PANC-1 cells were transfected with HA-tagged ubiquitin or K48 ubiquitin or K63 ubiquitin plasmids (**A**) or K48R mutant ubiquitin or K63 R mutant ubiquitin plasmids (**B**) and siControl or siVCPIP1 and then treated with 20 µM MG132 for 6 h. The cells were harvested and immunoprecipitated with an anti-YAP antibody, and the level of ubiquitinated YAP protein was examined by immunoblotting with an HA tag. **C**, **D** VCPIP1 overexpression decreased total ubiquitination and K48-linked polyubiquitination of the YAP protein but did not affect K63-linked polyubiquitination of the YAP protein, whereas overexpression of the VCPIP1 C219A mutant affected neither K48 nor K63-linked polyubiquitination of the YAP protein. HEK-293T cells were transfected with HA-tagged ubiquitin or K48 ubiquitin or K63 ubiquitin plasmids (**C**) or K48R mutant ubiquitin or K63R mutant ubiquitin plasmids (**D**), along with Flag-VCPIP1 WT or Flag-VCPIP1 C219A and Myc-YAP plasmids, and then treated with MG132 (20 µM) for 6 h. The cells were harvested and immunoprecipitated with an anti-Myc antibody, and the level of ubiquitinated YAP protein was examined by immunoblotting with an HA tag. **E** VCPIP1 removes the polyubiquitination of the YAP protein at the K252, K280 and K315 sites. HEK293T cells were transfected with HA-tagged ubiquitin and Flag-VCPIP1 and Myc-YAP WT or Myc-tagged point mutant forms of YAP (as shown in the panel). The cells were subsequently treated with MG132 (20 µM) for 6 h, harvested and immunoprecipitated with an anti-Myc antibody. The level of ubiquitinated YAP protein was examined by immunoblotting with an HA tag antibody.

Since VCPIP1 is an OTU domain-containing deubiquitinase, we further tested whether VCPIP1 affects the ubiquitination of the YAP protein. We performed a Co-IP assay to assess the level of ubiquitinated YAP protein. The results demonstrated that VCPIP1 depletion increased total ubiquitination and K48-linked polyubiquitination of the YAP protein but did not affect K63-linked ubiquitination (Fig. 6A). We validated this conclusion via the use of the dominant-negative ubiquitin mutants K48R and K63R. This study revealed that depleting VCPIP1 significantly reduced the degree of YAP ubiquitination via K48R mutant ubiquitin, whereas K63R mutant ubiquitin had a minimal effect (Fig. 6B). In addition, further ubiquitin assays revealed that the effect of VCPIP1 on YAP polyubiquitination is dependent on the intact catalytic function of VCPIP1, since the catalytically dead form of VCPIP (C219A) failed to affect YAP polyubiquitination (Fig. 6C, D). We further explored the specific ubiquitination sites on the YAP protein. Given that there are fourteen lysine sites available for ligation on the YAP protein, we generated the corresponding lysine mutant forms. The results of the ubiquitination assay demonstrated that VCPIP1 was capable of restraining the polyubiquitination of the YAP protein at specific locations, specifically K252, K280, and K315 (Fig. 6E).

### YAP transcriptionally regulates VCPIP1 expression in PAAD

**Fig. 7:**
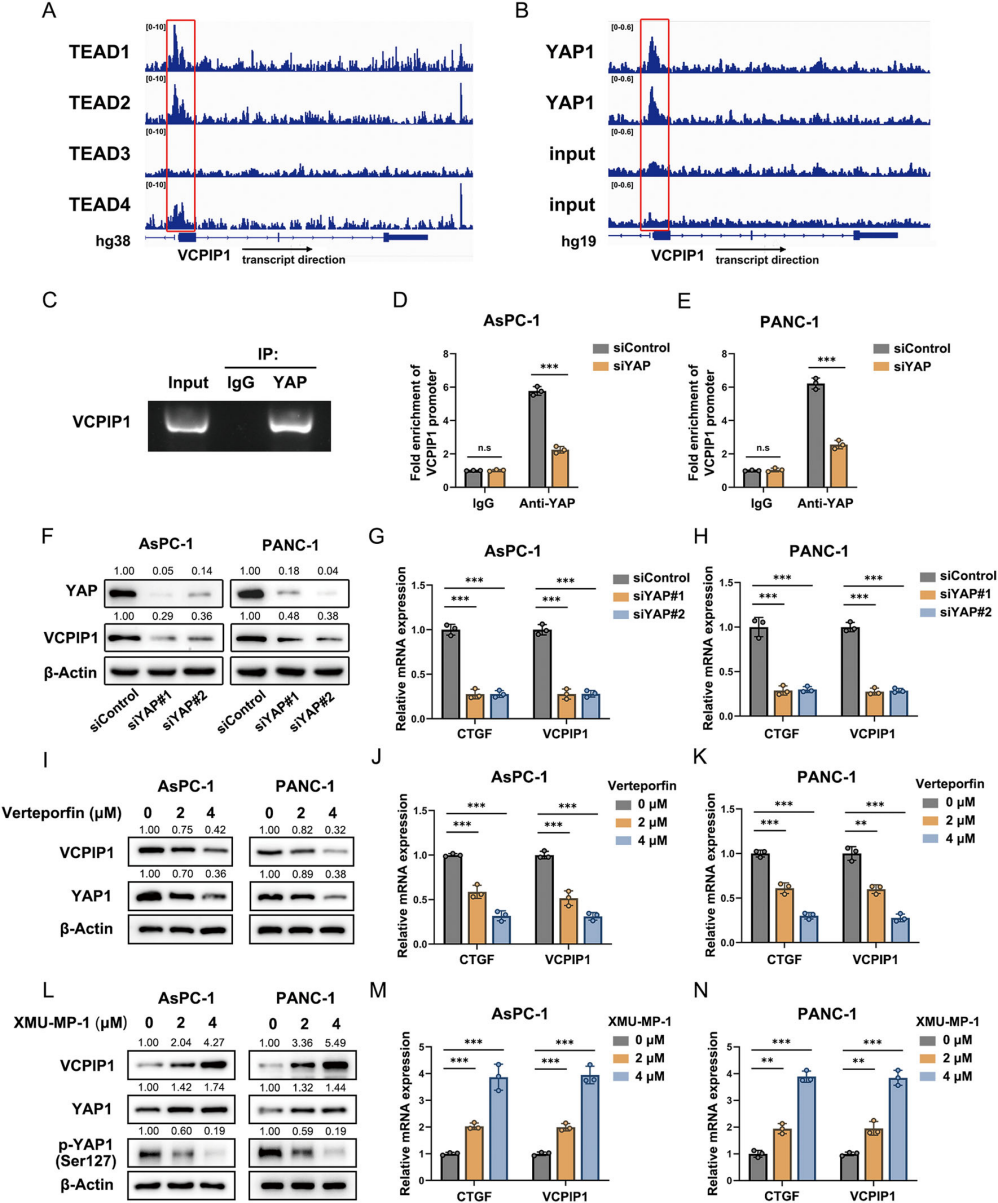
YAP transcriptionally regulates VCPIP1 expression in PAAD. ChIP-seq data analysis of TEAD (**A**) and YAP (**B**) revealed prominent peaks accumulating in the VCPIP1 promoter region. The data used in the analysis were TEAD1 (GSM2534071), TEAD2 (GSE91936), TEAD3 (GSM2534286), TEAD4 (GSE170161), YAP and input (GSE61852). The numbers on the upper left of each track indicate the fold change for TEAD1-4 and the peak reads for YAP and the input. **C** The binding of YAP to the VCPIP1 promoter region was verified via a ChIP assay. Fixed PANC-1 cells were subjected to chromatin immunoprecipitation with an anti-YAP antibody or with isotype IgG as a negative control. The enriched DNA was analyzed by gel electrophoresis. ChIP‒qPCR assay indicating decreased enrichment of the VCPIP1 promoter in AsPC-1 (**D**) and PANC-1 (**E**) cells after YAP knockdown. The cells were fixed, lysed and then immunoprecipitated with an anti-YAP antibody or isotype IgG. The enriched DNA was subjected to qPCR analysis. **F** PAAD cells were transfected with siControl or siYAP1, and the protein levels of VCPIP1 and YAP1 were examined by immunoblotting. **G**, **H** The mRNA expression levels of CTGF and VCPIP1 were measured via qRT‒PCR in AsPC-1 (**H**) and PANC-1 (**I**) cells after transfection with siControl or siYAP1. **I** AsPC-1 and PANC-1 cells were treated with the indicated concentrations of verteporfin for 48 h, and the protein levels of VCPIP1 were examined by immunoblotting. The mRNA expression levels of VCPIP1 and CTGF were measured via qRT‒PCR in AsPC-1 (**J**) and PANC-1 (**K**) cells after treatment with the indicated concentrations of verteporfin for 48 h. **L** AsPC-1 and PANC-1 cells were treated with the indicated concentrations of XMU-MP-1 for 48 h, and the protein levels of VCPIP1 were subsequently examined by immunoblotting. The mRNA expression levels of CTGF and VCPIP1 were measured via qRT‒PCR in AsPC-1 (**M**) and PANC-1 (**N**) cells after treatment with the indicated concentrations of XMU-MP-1 for 48 h. The experiments were performed in triplicate. All the data are presented as the means ± SDs. Statistical methods: Student’s t test for (**D**–**N**). **P < 0.01; ***P < 0.001.

On the basis of the important role of YAP in PAAD progression, we further analyzed ChIP sequence data from the Gene Expression Omnibus (GEO) database, which revealed distinct binding peaks on the promoter regions of VCPIP1 by YAP and TEADs (Fig. 7A, B). To confirm this binding, we performed chromatin immunoprecipitation to validate the interaction between YAP and the VCPIP1 promoter region (Fig. 7C). We further depleted YAP in PAAD cells via siRNA and observed decreased binding of YAP to VCPIP1 promoter regions, as detected by a ChIP assay (Fig. 7D, E). Consistently, YAP depletion in PAAD cells caused a decrease in the mRNA and protein levels of VCPIP1 (Fig. 7F–H). This conclusion was further confirmed through the YAP inhibitor verteporfin (VP). Treatment of AsPC-1 and PANC-1 cells with VP resulted in decreases in both the mRNA and protein levels of VCPIP1 (Fig. 7I–K; Fig. S2E). Moreover, the activation of YAP via XMU-MP-1 increased the mRNA and protein levels of VCPIP1 in AsPC-1 and PANC-1 cells (Fig. 7L–N).

### The VCPIP1 inhibitor CAS-12290-201 restrains PAAD progression

**Fig. 8:**
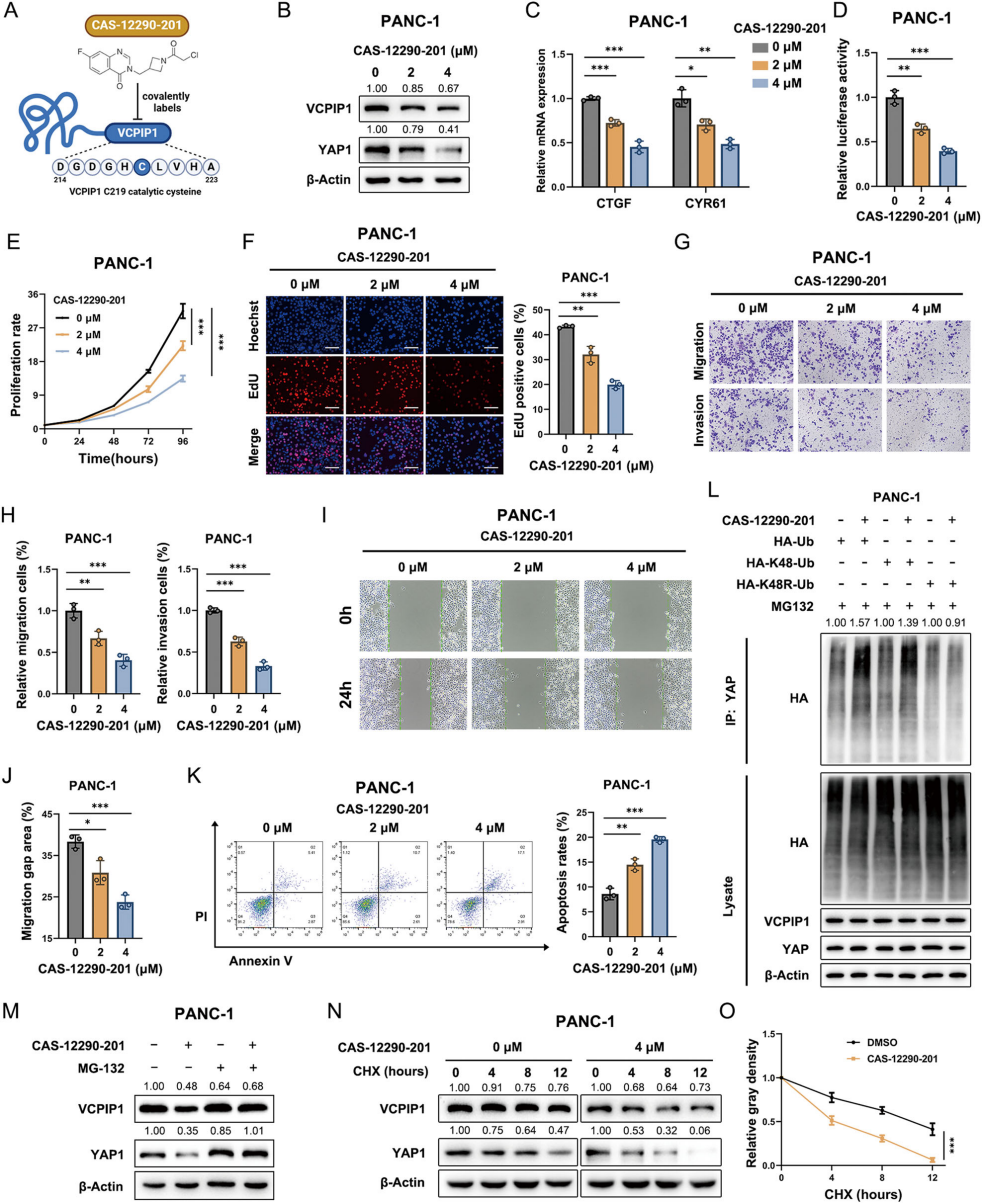
The VCPIP1 inhibitor CAS-12290-201 restrains PAAD progression. **A** Schematic diagram of the inhibitory effect of CAS-12290-201 on VCPIP1. CAS-12290-201 covalently labels the catalytic cysteine of VCPIP1 and inhibits its hydrolysis activity. **B** PANC-1 cells were treated with various concentrations of the VCPIP1 inhibitor CAS-12290-201 for 48 h, and the protein levels of VCPIP1 and YAP1 were examined by immunoblotting. **C** The mRNA expression levels of CTGF and CYR61 were measured via qRT‒PCR in PANC-1 cells after treatment with the indicated concentrations of CAS-12290-201 for 48 h. **D** A luciferase reporter gene assay was used to detect TEAD transcriptional activity in PANC-1 cells after treatment with the indicated concentrations of CAS-12290-201 for 48 h. The proliferation rate was assessed by CCK-8 (**E**) and EdU (**F**) assays in PANC-1 cells after treatment with the indicated concentrations of CAS-12290-201 for 48 h. The scale bar is 200 μm. **G**, **H** Migration and invasion abilities were assessed by Transwell assays in PANC-1 cells after treatment with the indicated concentrations of CAS-12290-201 for 48 h. **I**, **J** Cell migration ability was evaluated by a wound healing assay in PANC-1 cells after treatment with the indicated concentrations of CAS-12290-201 for 48 h. **K** PANC-1 cells were treated with 2 μM or 4 μM CAS-12290-201 for 48 h and stained with PI and FITC. Cell apoptosis was evaluated by flow cytometry. **L** The VCPIP1 inhibitor CAS-12290-201 increased K48-linked polyubiquitination of the YAP protein. PANC-1 cells transfected with HA-tagged ubiquitin, K48 ubiquitin or K48R mutant ubiquitin plasmids were treated with CAS-12290-201 (4 µM) for 48 h and MG132 (20 µM) for 6 h and then subjected to immunoprecipitation and immunoblotting. **M** PANC-1 cells were treated with 4 μM CAS-12290-201 for 48 h and 10 μM MG132 as indicated for 12 h. The protein levels of VCPIP1 and YAP were examined by immunoblotting. **N**, **O** The VCPIP1 inhibitor CAS-12290-201 decreases YAP protein stability. PANC-1 cells were treated with 4 μM CAS-12290-201 for 48 h and then treated with CHX (100 μM) at different time points. The protein levels of YAP were subsequently examined by immunoblotting at corresponding time points to assess changes in protein stability. The experiments were performed in triplicate. All the data are presented as the means ± SDs. Statistical methods: Student’s t test for (**C**, **D**, **F**–**K)**; two-way ANOVA for (**E**, **O**). *P < 0.05; **P < 0.01; ***P < 0.001.

CAS-12290-201 (CAS) is a fluoroquinazolinone that can selectively target the catalytic cysteine of VCPIP1 to inhibit its deubiquitinase activity (Fig. 8A) [32]. To evaluate the pharmaceutical value of VCPIP1 in treating PAAD, we utilized CAS to evaluate its effect on PAAD phenotypes. The western blot results revealed that CAS treatment reduced the protein level of YAP (Fig. 8B). The q-PCR data revealed that CAS treatment inhibited the expression of YAP target genes, including CTGF and CYR61 (Fig. 8C). A luciferase assay targeting the TEAD response element demonstrated that CAS treatment was capable of decreasing YAP activity in PAAD cells (Fig. 8D). We further examined the effect of CAS on the PAAD phenotype. The results of the CCK8 and EdU assays demonstrated that CAS treatment significantly inhibited PAAD proliferation (Fig. 8E, F). Moreover, the transwell assay results revealed that CAS treatment reduced the migration and invasion capacity of PAAD cells (Fig. 8G, H). The results of the wound-healing assay revealed that CAS treatment inhibited PAAD migration (Fig. 8I, J). The results of the apoptotic assay revealed that CAS treatment increased the percentage of apoptotic cells (Fig. 8K). We further examined the effect of CAS on YAP protein stability in detail. The western blot results revealed that after treatment with MG132, the ability of CAS to reduce the YAP protein level was diminished, indicating that CAS decreases the level of the YAP protein through the proteasome pathway (Fig. 8M). We performed a protein stability experiment by using CHX. The results showed that the VCPIP1 inhibitor CAS accelerated YAP protein degradation in PANC-1 cells (Fig. 8N, O). Further experiments aimed at assessing the ubiquitination level of YAP revealed that the VCPIP1 inhibitor CAS increased the K48-linked polyubiquitination of the YAP protein (Fig. 8L).

Since YAP and TAZ have partially overlapping and partially distinct functions in cancer, we set out to validate whether VCPIP1 also have a regulatory effect on TAZ protein. The results showed that VCPIP1 depletion or inhibition has no effect on TAZ protein levels, (Fig. S3A, B). Consistently, VCPIP1 overexpression has no impact on TAZ protein (Fig. S3C). Moreover, qPCR analysis showed that modulation of VCPIP1 protein did not alter TAZ mRNA levels (Fig. S3D–F). Collectively, these results indicate that VCPIP1 lacks a substantial regulatory effect on TAZ, and its biological function is mainly dependent on its modulation of YAP.

### The VCPIP1 inhibitor CAS-12290-201 sensitizes PAAD cells to gemcitabine treatment

**Fig. 9:**
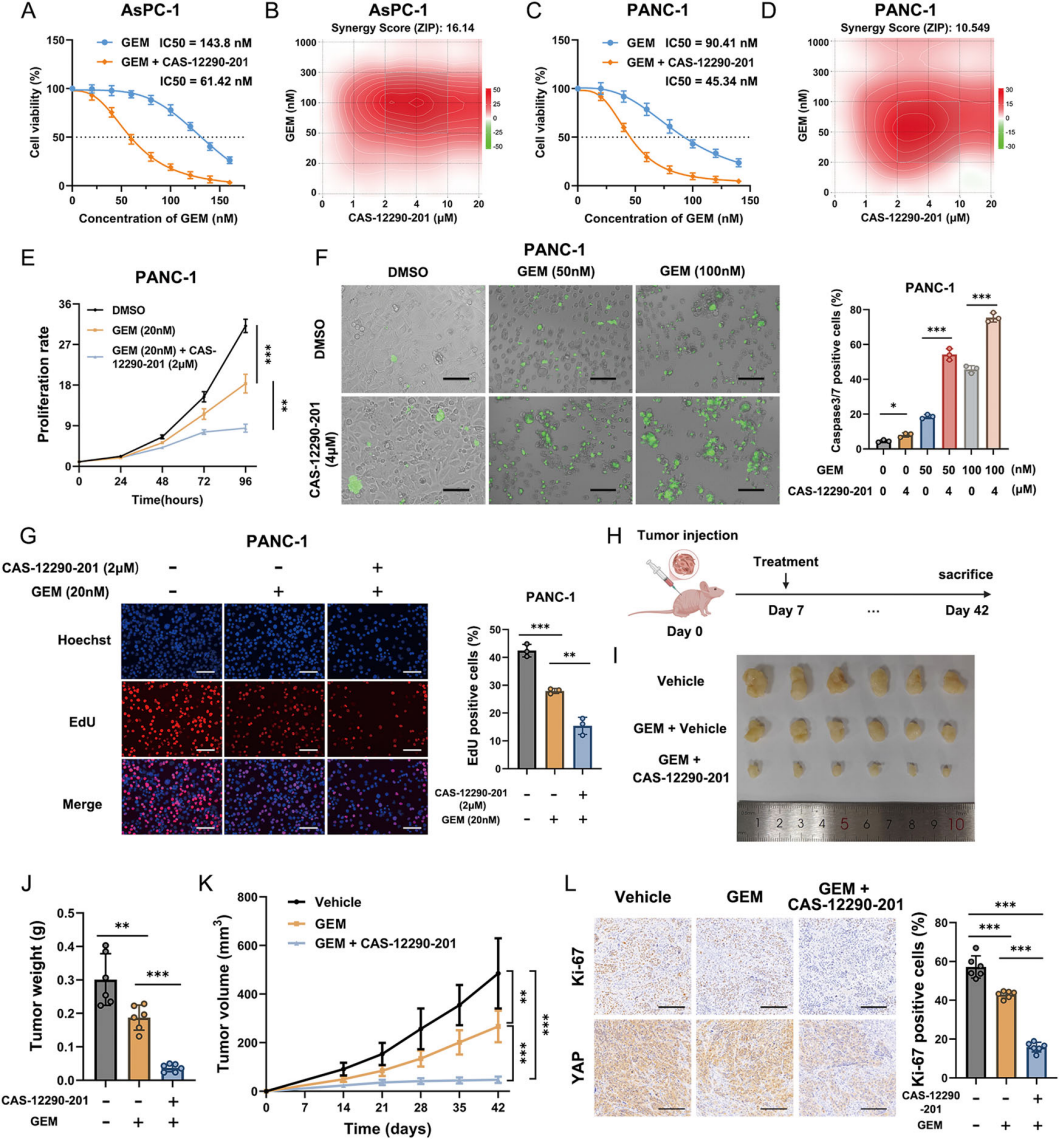
The VCPIP1 inhibitor CAS-12290-201 sensitizes PAAD cells to gemcitabine treatment. **A** AsPC-1 cells were pretreated with DMOS or 4 µM CAS-12290-201 for 48 h and then treated with the indicated concentrations of gemcitabine for 24 h. Cell viability was evaluated by a CCK-8 assay. The variable Hill slope model of nonlinear regression was employed to estimate the IC50. For the GEM group, the estimated IC50 was 143.8 nM. For the GEM + CAS-12290-201 group, the estimated IC50 was 61.42 nM. **B** The synergy score of the combined treatment of GEM and CAS-12290-201 in AsPC-1 cells was calculated via SynergyFinder 3.0 (synergyfinder.org). The cells were pretreated with the indicated concentrations of CAS-12290-201 for 48 h and then treated with the indicated concentrations of GEM for 24 h. Cell viability was evaluated via the CCK-8 assay, and the data were uploaded to the SynergyFinder website to calculate the synergy score. **C** PANC-1 cells were treated in the same way as described in (**A**). The estimated IC50 for the GEM group was 90.41 nM, and that for the GEM + CAS-12290-201 group was 45.34 nM. **D** Synergy score of the combination treatment of GEM and CAS-12290-201 in PANC-1 cells. Details are described in (**B**). **E** PANC-1 cells were treated with DMSO, 20 nM GEM or 20 nM GEM + 2 µM CAS-12290-201. Cell proliferation was evaluated by a CCK-8 assay. **F** PANC-1 cells were pretreated with 4 µM CAS-12290-201 for 48 h and then treated with 50 nM or 100 nM GEM for 24 h. Cell apoptosis was evaluated by using a caspase 3/7 green fluorescence reagent. The scale bar is 200 μm. **G** PANC-1 cells were treated with DMSO, 20 nM GEM or 20 nM GEM + 2 µM CAS-12290-201. Cell proliferation was evaluated by an EdU assay. The scale bar is 200 μm. **H**–**L** PANC-1 cells were inoculated subcutaneously into nude mice, and treatment started 7 days after inoculation. The mice were randomly assigned to three groups and subjected to different treatments. For the vehicle group, the mice were treated with vehicle solution (5% NMP, 5% Solutol, or 20% DMSO). For the GEM group, the mice were treated with gemcitabine (20 mg/kg, i.p., weekly). For the GEM + CAS-12290-201 group, the mice were treated with gemcitabine (10 mg/kg, i.p., weekly) or CAS-12290-201 (40 mg/kg, i.p., every day). Tumor weight (**J**) and volume (**K**) were measured. An IHC assay was used to evaluate Ki-67 and YAP expression (**L**). The percentage of Ki-67-positive cells was calculated. n = 6. The scale bar is 200 μm. The experiments were performed in triplicate. All the data are presented as the means ± SDs. Statistical methods: Student’s t test for (**F**, **G**, **J**, **L**); two-way ANOVA for (**E**, **K**). *P < 0.05; **P < 0.01; ***P < 0.001.

The chemotherapy drug gemcitabine (GEM) is widely used in PAAD treatment, but drug resistance is common in PAAD patients [33]. Recent studies have shown that YAP is highly involved in drug resistance [34]. Since VCPIP1 plays a crucial role in YAP stabilization, we further investigated whether the VCPIP inhibitor CAS could sensitize PAAD cells to GEM treatment. Following validation of YAP inhibition by CAS-12290-201 (Fig. S4A–D), we systematically evaluated the synergistic effects of CAS-12290-201 with gemcitabine (GEM) through a series of functional assays. The results of the CCK8 assay revealed that, compared with GEM alone, the combination of CAS and GEM improved the killing effect (Fig. 9A–D). In addition, CAS also synergized with GEM to enhance the anti-proliferative effect in PAAD cells (Fig. 9E). An apoptotic assay revealed that CAS synergized with GEM to increase the number of apoptotic PANC-1 cells (Fig. 9F). The results of the EdU incorporation assay further confirmed that CAS could also synergize with GEM to enhance the anti-proliferative effect in PAAD cells (Fig. 9G). We further evaluated the synergistic effect of GEM and CAS in a xenograft mouse model and showed that, compared with GEM alone, GEM plus CAS could further inhibit tumor growth (Fig. 9H–K). This conclusion was also confirmed via Ki67 staining of xenograft tumors (Fig. 9L).

## Discussion

**Fig. 10:**
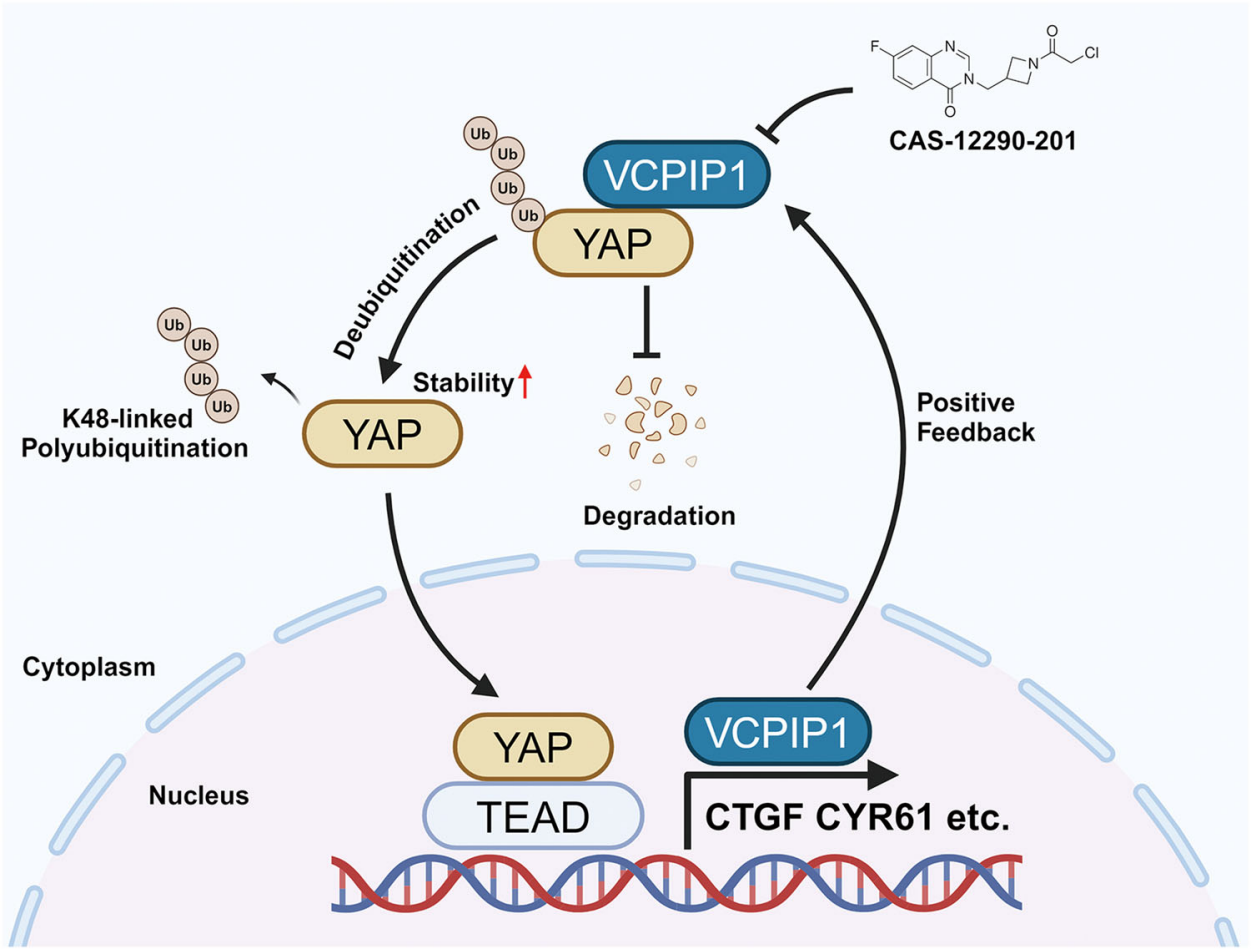
VCPIP1 forms a positive feedback loop and regulates the Hippo/YAP axis. VCPIP1 interacts with YAP and reduces the K48-linked ubiquitination of the YAP protein. As a result, it stabilizes the YAP protein and enhances the transcription of downstream target genes. VCPIP1 was identified as one of the target genes of the YAP/TEAD complex. Transcriptionally activated VCPIP1, in turn, stabilizes YAP, forming a positive feedback loop that ultimately contributes to pancreatic cancer progression.

In the present study, we revealed that VCPIP1 is a critical modulator of the Hippo/YAP axis in PAAD progression. VCPIP1 can stabilize YAP by inhibiting its K48-linked ubiquitination and degradation, thereby suppressing YAP-dependent PAAD progression. Targeting VCPIP1 via CAS could also sensitize PAAD to GEM-based therapy. YAP was observed to bind to the VCPIP1 promoter region, enhancing its transcription. This finding implies that VCPIP1 serves as both an upstream modulator and a downstream target of Hippo signaling in pancreatic cancer. We discovered a new positive feedback loop between VCPIP1 and Hippo signaling in PAAD progression. Therefore, blocking VCPIP1 could be a plausible strategy for treating YAP-driven pancreatic adenocarcinoma (Fig. 10).

Pancreatic cancer remains a challenging problem in the clinic, but therapeutic improvements have been limited in the past 30 years [1]. GEM-based chemotherapy has been the first-line therapy for unresectable pancreatic cancer [35], but only approximately 10%-30% of patients achieve significant clinical benefit. A novel approach for pancreatic adenocarcinoma therapy and corresponding targets are urgently needed. Despite the efforts of genome-wide association studies and molecular biology studies to uncover the driver molecules involved in pancreatic cancer progression, the detailed mechanisms are still awaiting discovery [36]. Recent studies revealed the critical roles of the Hippo/YAP axis in the progression of pancreatic adenocarcinoma [19, 37–39]. Further decoding the internal regulation of YAP is highly important for pancreatic cancer research and potential clinical application.

Since overactivation of the Hippo/YAP axis was observed in most PAAD samples, blocking the interaction between YAP and TEAD might be a promising approach for cancers driven by the Hippo signaling pathway [40, 41]. However, pharmacological drugs such as Super-TDU and verteporfin [40, 42, 43], which are proposed to block the Hippo/YAP axis, have failed to be translated into clinical applications in several preclinical studies on Hippo-driven cancers [44]. Thus, we shifted our strategy toward the identification of novel targets for YAP degradation. Through bioinformatics analysis of PAAD samples from the TCGA database, we discovered a set of DUBs (deubiquitinases) that were correlated with Hippo signaling activity. Further validation via siRNA indicated that VCPIP1 serves as a crucial modulator in regulating YAP activity and facilitating the progression of PAAD. We also revealed that VCPIP1 was correlated with YAP expression and poor survival in specimens from patients with pancreatic cancer. VCPIP1 interacts with the YAP protein and facilitates its deubiquitination, which subsequently stabilizes and promotes its function. Interestingly, the ChIP data indicated that YAP could bind to the promoter region of VCPIP1 and facilitate VCPIP1 transcription, which suggested the reciprocal regulation of VCPIP1 and the Hippo/YAP axis in PAAD. Targeting VCPIP1 pharmaceutically via CAS inhibited the progression of PAAD and enhanced sensitivity to GEM treatment, suggesting that blocking VCPIP1 might be a viable strategy for treating Hippo-driven pancreatic adenocarcinoma.

In general, our study revealed an unexpected positive feedback loop between VCPIP1 and Hippo signaling in pancreatic cancer. We found that the activation of VCPIP1 deubiquitinated and stabilized YAP, which subsequently enhanced the YAP transcriptome and tumorigenesis. On the other hand, YAP can directly bind to the VCPIP1 promoter region and facilitate its transcription. Our study demonstrated that VCPIP1 inhibition could inhibit PAAD progression in vivo and in vitro, suggesting that targeting VCPIP1 could provide an avenue to overcome PAAD progression.

## Supplementary information

Supplementary Figure 1

Supplementary Figure 2

Supplementary Figure 3

Supplementary Figure 4

supplementary figure legends

Supplementary Table.1 Clinicopathological correlation of VCPIP1 expression in PDAC.

VCPIP1 original data

IPMS_data_clear

## Data Availability

The results of mass spectrometry are available in the Supplementary files. The RNA-seq data can be found in the GEO database (GSE284347).
