# Supplementary figures and images for: VCPIP1 facilitates pancreatic adenocarcinoma progression via Hippo/YAP signaling
Source: Cell Death Dis. 2025 May 28;16(1):422. doi: 10.1038/s41419-025-07746-2 (PMC12120113; doi:10.1038/s41419-025-07746-2)

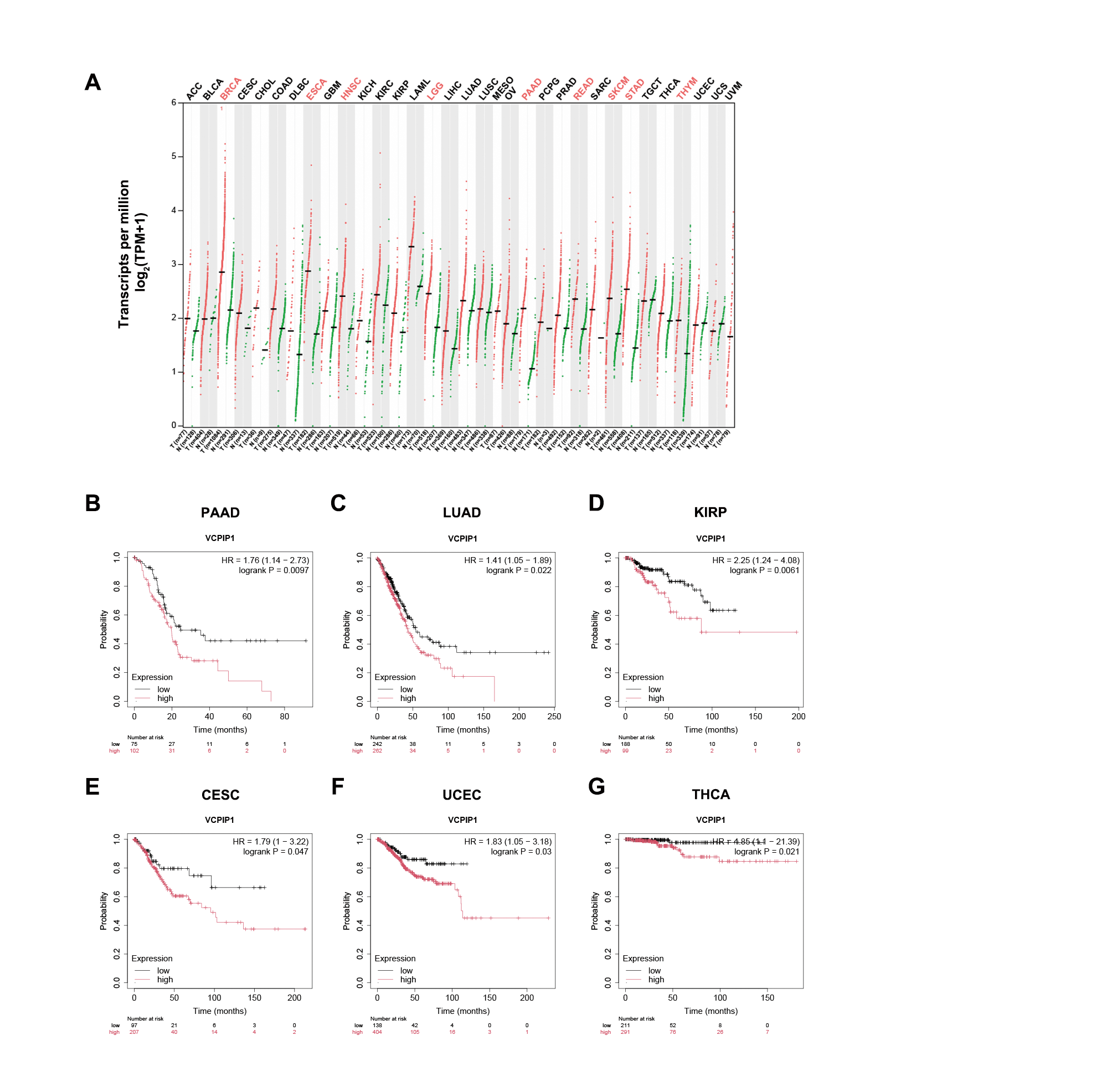

Supplement: Supplementary file 1 — Supplementary Figure 1 [file 41419_2025_7746_MOESM1_ESM.png]

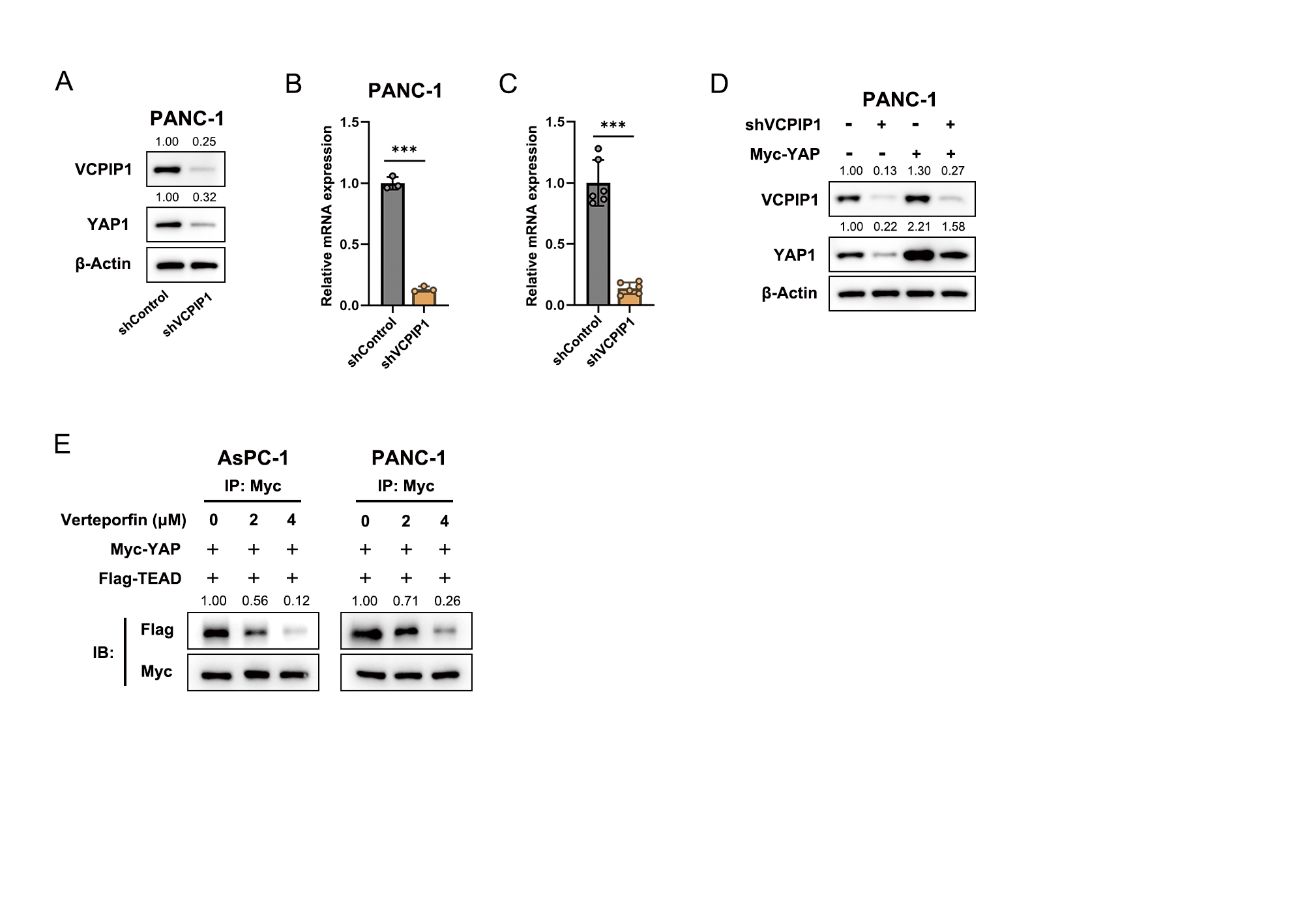

Supplement: Supplementary file 2 — Supplementary Figure 2 [file 41419_2025_7746_MOESM2_ESM.png]

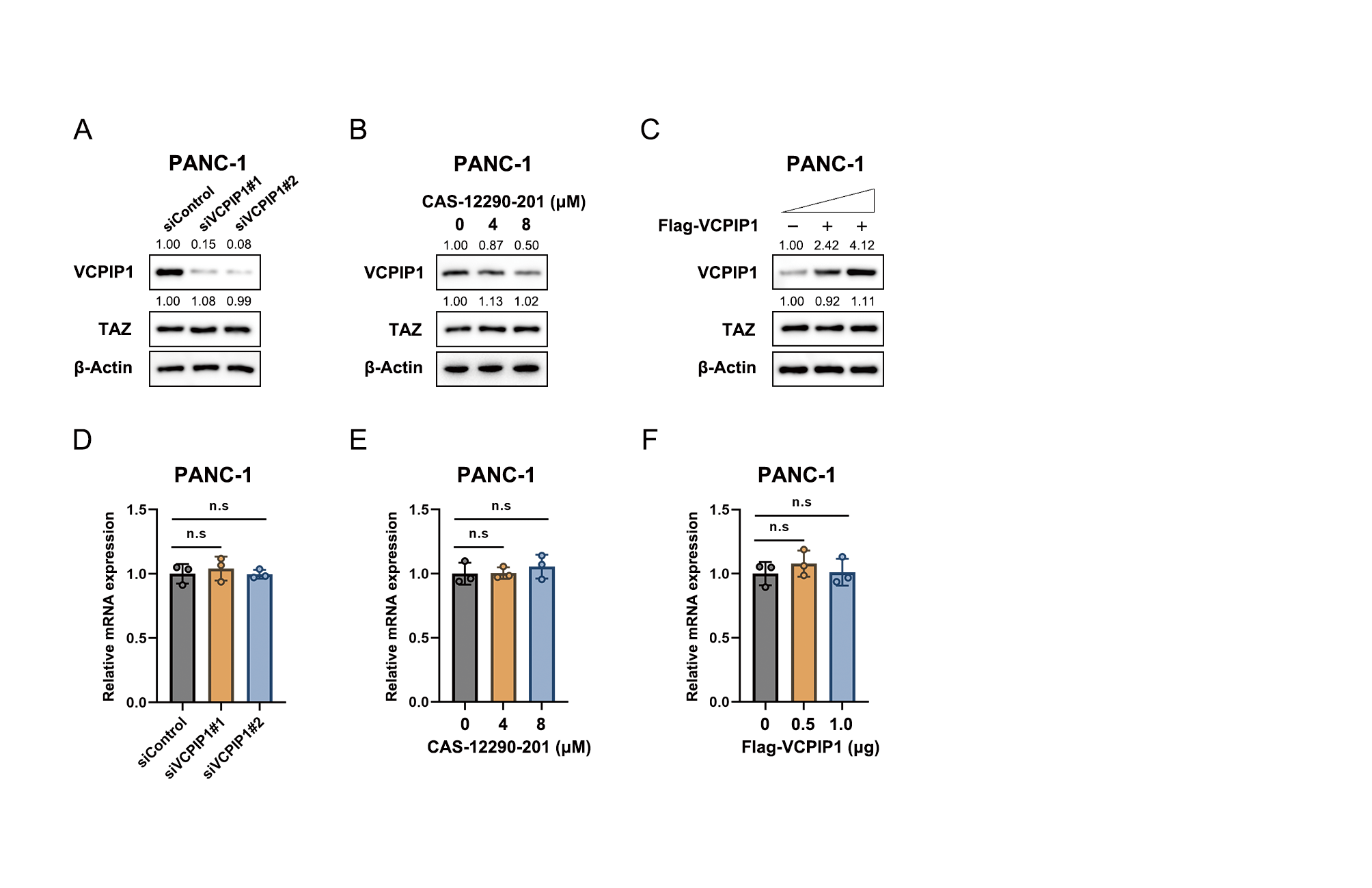

Supplement: Supplementary file 3 — Supplementary Figure 3 [file 41419_2025_7746_MOESM3_ESM.png]

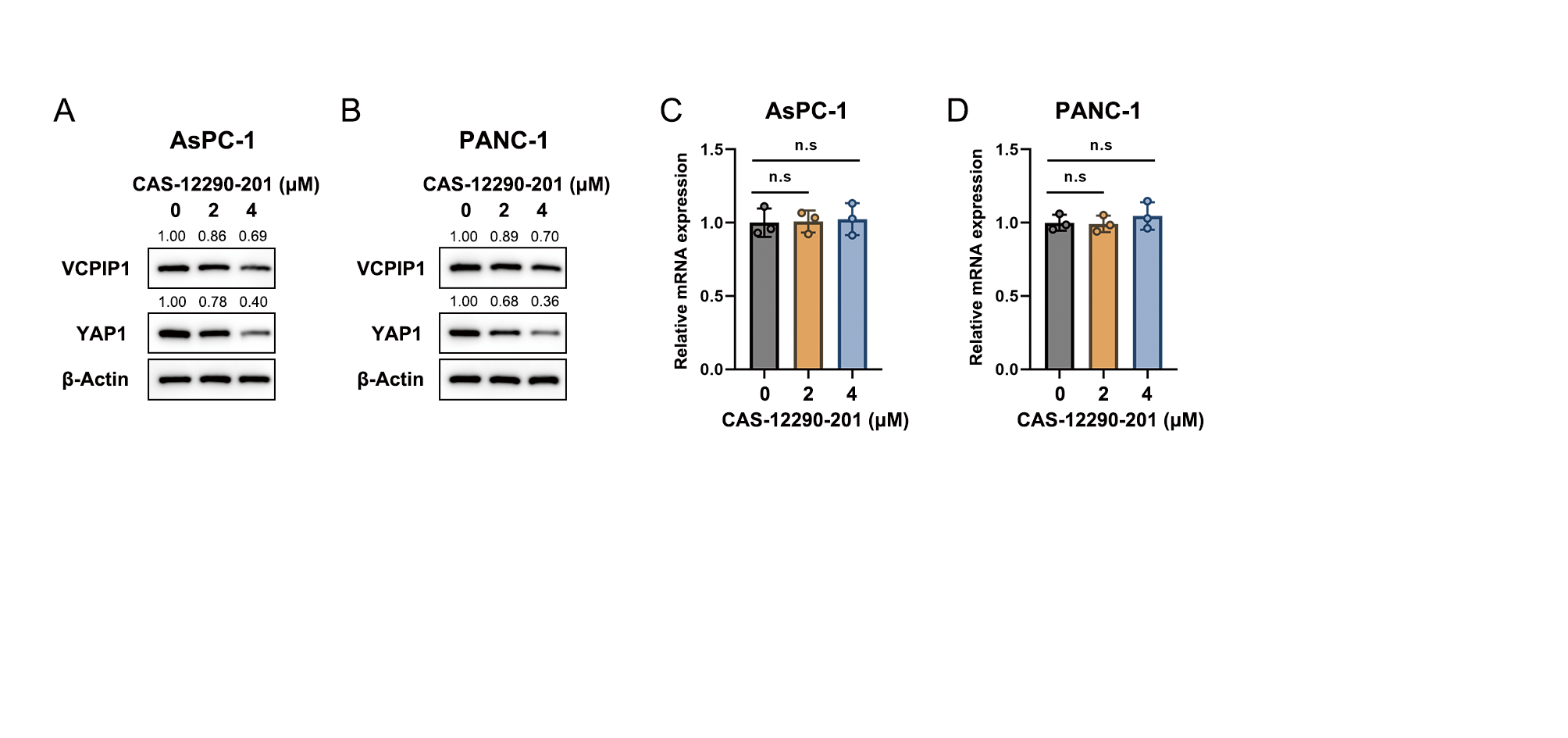

Supplement: Supplementary file 4 — Supplementary Figure 4 [file 41419_2025_7746_MOESM4_ESM.png]
