# Supplementary material for: VCPIP1 facilitates pancreatic adenocarcinoma progression via Hippo/YAP signaling
Source: Cell Death Dis. 2025 May 28;16(1):422. doi: 10.1038/s41419-025-07746-2 (PMC12120113; doi:10.1038/s41419-025-07746-2)
Supplement: Supplementary file 5 — supplementary figure legends [file 41419_2025_7746_MOESM5_ESM.docx]

**Figure. S1**

**A**: Expression profiles of VCPIP1 across 33 TCGA cancer types. Data analyzed and visualized using GEPIA2 (http://gepia2.cancer-pku.cn/). Normalized expression values (log2[TPM+1]) are shown as dot plots.

**B-G**: Kaplan-Meier analysis of overall survival (OS) in TCGA cohorts of PAAD, LUAD, KIRP, CESC, UCEC, THCA. Data analyzed and visualized using Kaplan-Meier plotter (https://kmplot.com/analysis/). Log-rank test p-values are shown.

PAAD: Pancreatic Adenocarcinoma; LUAD: Lung Adenocarcinoma; KIRP: Kidney Renal Papillary Cell Carcinoma; CESC: Cervical Squamous Cell Carcinoma; UCEC: Uterine Corpus Endometrial Carcinoma; THCA: Thyroid Carcinoma.

**Figure. S2**

**A**: Western-blot analysis of VCPIP1 and YAP1 protein expression in stable cell lines transfected with shControl or shVCPIP1.

**B**: RT-qPCR analysis of VCPIP1 mRNA expression in stable cell lines transfected with shControl and shVCPIP1.

**C**: RT-qPCR analysis of VCPIP1 mRNA expression in xenograft mouse tumors.

**D**: Western-blot analysis of VCPIP1 and YAP1 protein levels in stable cell lines transfected with shControl or shVCPIP1 plus vector or Myc-YAP plasmid.

E: Co-IP analysis of YAP-TEAD interaction after Verteporfin treatment (0, 2μM, 4μM) for 48 h.

The experiments were performed in triplicate. All the data are presented as the means ± SDs. Statistical methods: Student’s t test for **B-C**. ***P < 0.001.

**Figure. S3**

**A**: Western-blot analysis of VCPIP1 and TAZ protein levels in cells transfected with siControl, siVCPIP1#1 or siVCPIP1#2.

**B**: Western-blot analysis of VCPIP1 and TAZ protein levels in cells treated with CAS-12290-201 of indicated concentration (0, 4μM, 8μM) for 48 h.

**C**: Western-blot analysis of VCPIP1 and TAZ protein levels in cells transfected with vector, Flag-VCPIP1 0.5μg or Flag-VCPIP1 1.0μg.

**D**: RT-qPCR analysis of TAZ mRNA expression in cells transfected with siControl, siVCPIP1#1 or siVCPIP1#2.

**E**: RT-qPCR analysis of TAZ mRNA expression in cells treated with CAS-12290-201 of indicated concentration (0, 4μM, 8μM) for 48 h.

**F**: RT-qPCR analysis of TAZ mRNA expression in cells transfected with vector, Flag-VCPIP1 0.5μg or Flag-VCPIP1 1.0μg.

The experiments were performed in triplicate. All the data are presented as the means ± SDs. Statistical methods: Student’s t test for **D-F**.

**Figure. S4**

**A-B**: Western-blot analysis of VCPIP1 and YAP1 protein levels in AsPC-1 and PANC-1 cells treated with CAS-12290-201 of indicated concentration (0, 2μM, 4μM) for 48 h.

**C-D**: RT-qPCR analysis of YAP1 mRNA expression in AsPC-1 and PANC-1 cells treated with CAS-12290-201 of indicated concentration (0, 2μM, 4μM) for 48 h.

The experiments were performed in triplicate. All the data are presented as the means ± SDs. Statistical methods: Student’s t test for **C-D**.
