# Supplementary material for: VCPIP1 facilitates pancreatic adenocarcinoma progression via Hippo/YAP signaling
Source: Cell Death Dis. 2025 May 28;16(1):422. doi: 10.1038/s41419-025-07746-2 (PMC12120113; doi:10.1038/s41419-025-07746-2)
Supplement: Supplementary file 6 — Supplementary Table.1 Clinicopathological correlation of VCPIP1 expression in PDAC. [file 41419_2025_7746_MOESM6_ESM.docx]

Supplementary Table 1.

Clinicopathological correlation of VCPIP1 expression in PDAC.

| Characteristics | Cases (n = 57) | VCPIP1 expression | | |
| --- | --- | --- | --- | --- |
|  |  | Low (n = 26) | High (n = 31) | *P*-value |
| Gender |  |  |  |  |
| Male | 30 | 13 | 17 | 0.716 |
| Female | 27 | 13 | 14 |  |
| Age (years) |  |  |  |  |
| ≤60 | 31 | 14 | 17 | 0.940 |
| >60 | 26 | 12 | 14 |  |
| Differentiation |  |  |  |  |
| low | 12 | 5 | 7 | 0.423 |
| mid | 28 | 11 | 17 |  |
| high | 17 | 10 | 7 |  |
| Clinical stage |  |  |  |  |
| I | 23 | 17 | 6 | **0.001**** |
| II | 22 | 7 | 15 |  |
| III+IV | 12 | 2 | 10 |  |
| T stage |  |  |  |  |
| T1 + T2 | 31 | 17 | 14 | 0.127 |
| T3 + T4 | 26 | 9 | 17 |  |
| N stage |  |  |  |  |
| No | 38 | 23 | 15 | **0.001**** |
| Yes | 19 | 3 | 16 |  |

The correlation analysis between VCPIP1 and the clincial pathological characteristics in 57

PDAC samples. Statistical significance (*P* < 0.05) is shown in bold. ***P* < 0.01
