# Supplementary material for: VCPIP1 facilitates pancreatic adenocarcinoma progression via Hippo/YAP signaling
Source: Cell Death Dis. 2025 May 28;16(1):422. doi: 10.1038/s41419-025-07746-2 (PMC12120113; doi:10.1038/s41419-025-07746-2)

Figure 2A

AsPC-1

siControl	siVCPIP1#1	siVCPIP1#2
1.01208591	0.13849294	0.16197244
1.13535111	0.13202108	0.14314574
0.87026683	0.16001876	0.10935526

PANC-1

siControl	siVCPIP1#1	siVCPIP1#2
1.05772906	0.13128464	0.16443006
1.02879455	0.17385243	0.16392616
0.91896063	0.12941865	0.14984455

Figure 2B

AsPC-1

	CTGF			CYR61		
siControl	1.07067828	0.92440115	1.0103702	1.013082805	0.90961923	1.08516412
siVCPIP1#1	0.46494196	0.52074572	0.46049593	0.41947768	0.55468426	0.40579097
siVCPIP1#2	0.53937534	0.61571871	0.58148669	0.496637755	0.48555045	0.46130562

PANC-1

	CTGF			CYR61		
siControl	1.06335414	1.02506809	0.91742244	1.14487981	0.98726103	0.88472461
siVCPIP1#1	0.43118106	0.49509533	0.41319828	0.52157839	0.49478144	0.42948337
siVCPIP1#2	0.48605501	0.46174659	0.4231937	0.48013916	0.45014542	0.4717901

Figure 2C

AsPC-1	siControl	siVCPIP1#1	siVCPIP1#2
	1.022496472	0.359700162	0.383645769
	0.952671064	0.370624493	0.473651987
	1.024832463	0.435571928	0.441308892

Figure 2D

PANC-1	siControl	siVCPIP1#1	siVCPIP1#2
	0.960626887	0.417109624	0.445237459
	1.063088611	0.367164536	0.360832399
	0.976284501	0.396968967	0.418202237

Figure 2F

AsPC-1

time (h)	proliferation rate								
0	1	1	1	1	1	1	1	1	1
24	3.361814	3.164315	3.194274	2.240179	2.366041	2.340641	2.277577	2.452236	2.618056
48	7.560127	6.637097	7.375256	5.133929	4.887372	4.905565	5.001808	5.46748	5.83631
72	17.36392	16.19254	17.27096	9.866071	9.351536	9.53204	9.524412	9.60874	10.74306
96	30.30169	27.5877	28.41513	17.12589	16.48464	16.53204	16.60127	17.23577	18.27679
	siControl			siVCPIP1#1			siVCPIP1#2		

PANC-1

time (h)	proliferation rate								
0	1	1	1	1	1	1	1	1	1
24	2.085179	2.082443	1.984211	1.695883	1.911548	1.847482	2.225554	1.942931	1.908976
48	6.155026	6.00916	6.612281	3.160691	3.538084	3.854676	3.623207	3.592737	3.730721
72	14.37479	13.66565	14.81404	7.12085	7.255528	7.939568	7.138201	7.433204	7.151707
96	29.70358	27.1084	32.06316	14.52324	13.04914	14.70216	13.69361	13.43061	13.39949
	siControl			siVCPIP1#1			siVCPIP1#2		

Figure 2F

AsPC-1

time (h)	proliferation rate								
0	1	1	1	1	1	1	1	1	1
24	3.361814	3.164315	3.194274	2.240179	2.366041	2.340641	2.277577	2.452236	2.618056
48	7.560127	6.637097	7.375256	5.133929	4.887372	4.905565	5.001808	5.46748	5.83631
72	17.36392	16.19254	17.27096	9.866071	9.351536	9.53204	9.524412	9.60874	10.74306
96	30.30169	27.5877	28.41513	17.12589	16.48464	16.53204	16.60127	17.23577	18.27679
	siControl			siVCPIP1#1			siVCPIP1#2		

PANC-1

time (h)	proliferation rate								
0	1	1	1	1	1	1	1	1	1
24	2.085179	2.082443	1.984211	1.695883	1.911548	1.847482	2.225554	1.942931	1.908976
48	6.155026	6.00916	6.612281	3.160691	3.538084	3.854676	3.623207	3.592737	3.730721
72	14.37479	13.66565	14.81404	7.12085	7.255528	7.939568	7.138201	7.433204	7.151707
96	29.70358	27.1084	32.06316	14.52324	13.04914	14.70216	13.69361	13.43061	13.39949
	siControl			siVCPIP1#1			siVCPIP1#2		

Figure 2G

siControl	siVCPIP1#1	siVCPIP1#2
0.453125	0.2387707	0.2459016
0.438119	0.225974	0.2406948
0.432836	0.2136986	0.2341463

Figure 2H

siControl	siVCPIP1#1	siVCPIP1#2
0.431325	0.234414	0.2334906
0.425316	0.2233766	0.2230769
0.417062	0.2227723	0.2130751

Figure 2I

AsPC-1

siControl	siVCPIP1#1	siVCPIP1#2
1.120916	0.506947	0.502441
0.98911	0.499061	0.449493
0.889974	0.478783	0.448367

PANC-1

siControl	siVCPIP1#1	siVCPIP1#2
0.949615	0.460581	0.448133
0.992294	0.606402	0.517487
1.058091	0.583284	0.469472

Figure 2J

AsPC-1

siControl	siVCPIP1#1	siVCPIP1#2
1.02009	0.390256	0.415871
0.994475	0.379709	0.3556
0.985434	0.379709	0.302863

PANC-1

siControl	siVCPIP1#1	siVCPIP1#2
1.121588	0.513648	0.42928
0.962779	0.476427	0.414392
0.915633	0.35732	0.364764

Figure 2K

siControl	siVCPIP1#1	siVCPIP1#2
0.36288	0.277543	0.276547
0.362632	0.29816	0.282044
0.359117	0.304814	0.26876

Figure 2L

siControl	siVCPIP1#1	siVCPIP1#2
0.391467	0.296744	0.292558
0.38874	0.284568	0.280438
0.380053	0.27552	0.279405

Figure 2M

siControl	siVCPIP1#1	siVCPIP1#2
8.98	15.18	14.76
9.48	15.74	16.12
9.63	17.08	16.86

Figure 2N

siControl	siVCPIP1#1	siVCPIP1#2
8.2	16.33	16.29
9.55	16.77	16.86
9.71	17.96	17.09

Figure 2O

shControl	shVCPIP1
0.2779	0.0774
0.223	0.0732
0.2342	0.0643
0.1953	0.0651
0.203	0.0702
0.1883	0.0575

Figure 2P

	shControl					shVCPIP1				
weeks	2	3	4	5	6	2	3	4	5	6
volume	81.9396	136.0141	227.5744	322.198	457.3021	22.35177	42.9821	69.67241	101.5721	125.2544
	86.98497	129.0411	228.6435	341.8629	485.6226	22.7495	34.72806	57.9005	77.63213	112.592
	63.55516	114.8811	152.5684	233.4391	355.175	15.99469	30.93125	47.42343	60.25003	90.65297
	44.34794	75.2535	115.5753	182.9321	260.6122	19.93136	30.32822	59.7849	71.48974	104.2639
	47.4016	78.23632	145.5261	207.3066	281.75	21.37625	33.88327	63.7245	76.0384	113.7824
	62.87002	117.4195	165.6776	296.7525	364.5912	11.73758	21.805	40.86742	53.96327	72.21244

Figure 2Q

shControl	shVCPIP1
0.577444	0.348181
0.613303	0.314379
0.5623	0.339033
0.437092	0.2639
0.497622	0.326247
0.550539	0.350703

Figure 3B

AsPC-1	CTGF	CYR61
vector	1.02836281	0.90190143
	0.87986536	1.2056503
	1.1051912	0.91964362
VCPIP1 WT	1.55860666	2.11929133
	1.77203749	1.60690176
	1.56851468	1.64712228
VCPIP1 C219A	1.16304504	1.14541113
	0.92950669	1.03953188
	0.97432821	0.92661005

PANC-1	CTGF	CYR61
vector	0.97371515	1.13519857
	1.12713465	0.94569367
	0.91115502	0.93148889
VCPIP1 WT	2.62084807	3.81074525
	2.76450244	4.12970362
	3.08894524	4.74059194
VCPIP1 C219A	0.93377967	1.04510615
	1.0678468	0.94297544
	0.98186667	1.01218935

Figure 3C

VECTOR	VCPIP1WT	VCPIP1C219A
1.069982	4.171887781	1.152586435
0.910854	3.881862252	0.885041743
1.019165	4.277820006	0.970167227

Figure 3D

VECTOR	VCPIP1WT	VCPIP1C219A
0.944137	4.327345558	0.927205495
1.07988	4.425328774	1.082980038
0.975983	4.052776528	0.981932406

Figure 3F

AsPC-1

time(h)	proliferation rate								
0	1	1	1	1	1	1	1	1	1
24	3.051813	2.836094	3.25791	4.037775	4.467685	4.053862	3.017442	2.93627	3.020913
48	6.396373	6.068875	7.021093	9.205666	10.11751	8.814024	6.907946	6.604499	6.784221
72	16.14594	15.32171	16.86098	22.7681	22.76968	21.14329	15.84884	14.86973	15.20627
96	26.22884	25.02877	27.19847	31.56978	34.2832	30.43394	25.77229	24.18744	25.19487
	Vector			VCPIP1 WT			VCPIP1 C219A		

PANC-1

time(h)	proliferation rate								
0	1	1	1	1	1	1	1	1	1
24	1.656477	1.798675	1.78582	3.463366	3.310458	3.207815	1.774286	1.678445	1.581242
48	4.921397	4.29404	4.633678	7.718812	6.831699	6.950266	5.121429	4.994111	5.412153
72	12.70451	11.68742	12.74594	21.75446	17.81209	20.93606	13.76571	11.37338	12.85733
96	24.23435	23.1245	25.99409	38.20396	31.33824	34.15631	27.24286	24.00942	26.07398
	Vector			VCPIP1 WT			VCPIP1 C219A		

Figure 3G

vector	VCPIP1 WT	VCPIP1 C219A
0.453826	0.5454545	0.453125
0.449749	0.4935622	0.438118812
0.459016	0.5382586	0.432835821

Figure 3H

vector	VCPIP1 WT	VCPIP1 C219A
0.425316	0.5418605	0.452173913
0.423529	0.5104167	0.431325301
0.430657	0.5333333	0.417061611

Figure 3I

migration					
AsPC-1			PANC-1		
vector	VCPIP1 WT	VCPIP1 C219A	vector	VCPIP1 WT	VCPIP1 C219A
114.44%	144.45%	107.17%	106.02%	155.40%	112.42%
93.88%	135.68%	95.66%	98.75%	149.23%	93.90%
91.69%	131.29%	87.71%	95.22%	134.24%	90.82%

Figure 3J

invasion					
AsPC-1			PANC-1		
vector	VCPIP1 WT	VCPIP1 C219A	vector	VCPIP1 WT	VCPIP1 C219A
103.42%	147.47%	108.47%	101.28%	175.73%	103.19%
98.68%	137.05%	104.37%	86.77%	159.85%	99.09%
97.89%	129.63%	93.79%	111.95%	153.56%	112.23%

Figure 3K

vector	VCPIP1 WT	VCPIP1 C219A
0.467596	0.36721751	0.380287989
0.46868	0.369927886	0.377754063
0.45476	0.361318232	0.356651318

Figure 3L

vector	VCPIP1 WT	VCPIP1 C219A
0.474594	0.374238702	0.385227686
0.464466	0.369554328	0.366441264
0.461307	0.351534045	0.350483703

Figure 3M

vector	VCPIP1 WT	VCPIP1 C219A
10.68	6.43	9.73
10.82	6.59	10.49
11.19	6.8	11

Figure 3N

vector	VCPIP1 WT	VCPIP1 C219A
9.66	5.88	9.83
9.96	6.3	10.16
10.78	6.48	11.38

Figure 4B

	CTGF	CYR61
siControl	1.026931	0.917759
	0.867268	1.083191
	1.122808	1.005927
siVCPIP1	0.425692	0.462412
	0.390842	0.456961
	0.435352	0.477783
Myc-YAP	4.117073	3.997172
	3.929262	3.807058
	3.881542	4.287214
siVCPIP1 + Myc-YAP	1.392905	1.591227
	1.300464	1.214764
	1.586913	1.619801

Figure 4C

siControl	siVCPIP1	Myc-YAP	siVCPIP1+Myc-YAP
0.921433	0.457381	4.267586	1.317885179
1.099645	0.420995	3.996115	1.380679512
0.978922	0.376116	4.614955	1.548423875

Figure 4D

time(h)	siControl			siVCPIP1			Myc-YAP			siVCPIP1+Myc-YAP		
0	1	1	1	1	1	1	1	1	1	1	1	1
24	3.069767	2.676568	2.613027	2	1.731034	2.003257	6.702586	8.134409	7.272277	2.715686	3.12945	3.727642
48	8.569767	8.419142	9.398467	4.482143	4.558621	4.29316	15.24569	17.33333	14.88119	10.41176	10.01294	11.87805
72	18.14729	16.55446	18.12644	8.428571	8.489655	6.990228	32.31034	40.00538	36.28218	19.5098	20.11327	25.65447
96	34.94961	31.38614	35.42146	16.65	18.61379	17.57655	68.43103	87.39247	76.06436	43.51961	42.99676	53.42276

Figure 4E

siControl	siVCPIP1	Myc-YAP	siVCPIP1+Myc-YAP
0.390164	0.186486	0.556995	0.40530303
0.3875	0.175127	0.517572	0.429333333
0.371795	0.172662	0.576923	0.454545455

Figure 4G

siControl	siVCPIP1	Myc-YAP	siVCPIP1+Myc-YAP
0.362339	0.274609	0.511006	0.370551968
0.340968	0.262442	0.500091	0.374837651
0.339533	0.232334	0.486614	0.358944588

Figure 4I

migration			
siControl	siVCPIP1	Myc-YAP	siVCPIP1+Myc-YAP
1.074627	0.567164	2.211443	1.358208955
0.995025	0.49005	2.062189	1.246268657
0.930348	0.447761	1.736318	1.201492537
invasion			
siControl	siVCPIP1	Myc-YAP	siVCPIP1+Myc-YAP
1.057836	0.548507	1.648321	1.360074627
0.990672	0.45056	1.597948	1.231343284
0.951493	0.439366	1.701493	1.013059701

Figure 4J

siControl	siVCPIP1	Myc-YAP	siVCPIP1+Myc-YAP
9.73	9.73	4.86	8.69
9.9	9.9	5.94	9.05
10.52	13.66	6.14	9.19

Figure 4L

siControl	siVCPIP1	Myc-YAP	siVCPIP1+Myc-YAP
0.2301	0.0955	0.7019	0.4816
0.2919	0.0684	0.6754	0.3219
0.2425	0.0743	0.7133	0.3128
0.2256	0.0772	0.712	0.3137
0.1875	0.074	0.5869	0.2746
0.2541	0.048	0.5944	0.3298

Figure 4M

shControl					
week	2	3	4	5	6
volume	62.75912	120.2234	193.1857	282.6706	380.1635
	70.89727	117.6226	174.0469	288.8085	375.9208
	74.41875	122.0586	201.9246	278.9243	385.687
	75.33664	132.0945	222.6995	274.8351	407.8368
	57.55295	99.144	184.2262	240.3477	335.0676
	75.16087	136.6118	212.7178	316.1879	398.426

shVCPIP1					
week	2	3	4	5	6
volume	31.11279	64.0775	75.34652	120.902	146.8408
	21.35097	48.97989	53.66088	73.2982	105.7055
	21.14892	37.69599	54.44368	71.70031	94.60164
	18.90657	42.86506	64.76503	80.93193	103.0261
	23.17005	47.385	52.96282	75.06173	107.235
	11.55465	27.75518	32.41706	44.89729	60.45206

Myc-YAP					
week	2	3	4	5	6
volume	173.3255	322.3925	557.3488	757.4804	1075.066
	141.256	267.9515	456.7194	644.0799	903.5166
	126.711	238.3099	339.4872	502.0186	757.2664
	168.9431	280.4448	445.9282	723.9991	995.8748
	120.9249	191.9322	336.2135	518.6726	716.9326
	89.50469	150.937	278.2153	389.4976	544.1653

siVCPIP1+Myc-YAP					
week	2	3	4	5	6
volume	130.7723	233.2463	386.5853	571.2825	707.8093
	91.23016	136.2231	233.9964	366.3734	462.0206
	82.36848	124.4786	202.5235	339.1388	436.7664
	99.01861	176.4286	316.4745	444.8015	557.2642
	73.16886	148.8128	241.1447	297.909	425.3991
	77.3353	119.556	189.6291	301.695	392.1086

Figure 4N

siControl	siVCPIP1	Myc-YAP	siVCPIP1+Myc-YAP
0.518231	0.288265	0.756835	0.577790148
0.512252	0.356366	0.75991	0.661793646
0.477166	0.243607	0.788752	0.560662289
0.552994	0.327349	0.776504	0.657593123
0.469813	0.332624	0.71378	0.517973856
0.443458	0.253207	0.681548	0.601864934

Figure 5L

Time(hour)	siControl			siVCPIP1		
0	1	1	1	1	1	1
4	0.923907	0.847328	0.856657	0.423857	0.496823	0.436984
8	0.746166	0.613882	0.683216	0.110276	0.186537	0.130658
12	0.328613	0.276578	0.394683	0.028397	0.079672	0.032698

Figure 5O

Time(hour)	siControl			siVCPIP1		
0	1	1	1	1	1	1
4	0.840864	0.806972	0.882487	0.433784	0.465962	0.410976
8	0.62756	0.594681	0.696812	0.183241	0.226814	0.166136
12	0.343471	0.312543	0.41967	0.031608	0.086312	0.047951

Figure 5Q

Time(hour)	Vector			VCPIP1^{WT}			VCPIP1^{C219A}		
0	1	1	1	1	1	1	1	1	1
4	0.776234	0.732256	0.687618	0.984734	0.957906	0.931106	0.807427	0.728753	0.773429
8	0.485392	0.44917	0.404863	0.795067	0.818068	0.764159	0.544431	0.439976	0.467298
12	0.342092	0.311098	0.264773	0.673987	0.729318	0.654043	0.370714	0.293901	0.324195

Figure 7D

AsPC-1	siControl			siYAP		
IgG	1.01284	0.984277	1.002882	1.057922	0.961193	1.026258
anti-YAP	5.753082	5.523426	6.046068	2.238891	2.449502	2.056555

Figure 7E

PANC-1	siControl			siYAP		
IgG	0.97901	1.029181	0.991809	1.035297	1.154384	0.936424
anti-YAP	6.252711	5.868606	6.525758	2.53578	2.818046	2.313274

Figure 7G

AsPC-1	siControl			siYAP#1			siYAP#2		
CTGF	1.0153524	1.05126	0.933388	0.238009	0.339704	0.254518	0.242503	0.318858	0.272509
VCPIP1	1.0628114	0.947992	0.989197	0.276622	0.335588	0.221948	0.285208	0.316099	0.242288

Figure 7H

PANC-1	siControl			siYAP#1			siYAP#2		
CTGF	1.0705643	1.054313	0.875122	0.244218	0.345182	0.273403	0.275662	0.336298	0.28454
VCPIP1	1.0590116	0.981032	0.959956	0.257088	0.320358	0.250589	0.266745	0.314318	0.278084

Figure 7J

AsPC-1	0uM			2uM			4uM		
CTGF	1.017393	0.996499	0.986108	0.670405	0.559823	0.530302	0.256487	0.365963	0.329485
VCPIP1	1.048495	0.975142	0.976362	0.594778	0.429701	0.521267	0.32698	0.344954	0.263479

Figure 7K

PANC-1	0uM			2uM			4uM		
CTGF	0.956679	1.022558	1.020763	0.631369	0.542914	0.658185	0.261063	0.315952	0.328077
VCPIP1	1.025281	1.060406	0.914314	0.62448	0.543616	0.629882	0.32595	0.242315	0.265915

Figure 7M

AsPC-1	0uM			2uM			4uM		
CTGF	1.019468	1.037484	0.943048	1.953819	1.962518	2.17016	3.893539	3.365774	4.339107
VCPIP1	1.02757	1.046718	0.925712	1.952014	2.147303	1.883911	4.329942	3.745309	3.768507

Figure 7N

PANC-1	0uM			2uM			4uM		
CTGF	1.120973	1.012648	0.86638	1.93656	1.755868	2.131978	3.782009	3.753233	4.128088
VCPIP1	1.02801	1.047363	0.924628	1.823555	2.249554	1.788838	4.127404	3.845303	3.563199

Figure 8C

	0μM			2μM			4μM		
CTGF	1.018173	0.977171	1.005097	0.76375	0.721755	0.691145	0.522082	0.391145	0.449192
CYR61	0.967611	1.112662	0.92883	0.736728	0.635658	0.748866	0.483603	0.434689	0.539117

Figure 8D

0μM	2μM	4μM
1.072123	0.655722	0.389996
0.922397	0.594437	0.369196
1.005481	0.696011	0.429282

Figure 8E

time (h)	proliferation rate								
0	1	1	1	1	1	1	1	1	1
24	2.359606	2.445159	2.177174	2.017776	2.193474	1.921904	1.77718	1.856913	1.710121
48	6.442843	6.116726	5.819921	5.212987	5.540399	4.973358	3.638374	3.862124	3.515104
72	15.39021	15.77282	14.99118	10.6311	11.37239	9.817427	7.072708	7.464887	6.992929
96	31.26956	33.54362	29.66912	22.57315	23.22954	20.85656	13.43433	14.2501	12.6162
	0μM			2μM			4μM		

Figure 8F

0μM	2μM	4μM
0.4375	0.349398	0.21513
0.434783	0.328261	0.200514
0.426923	0.285171	0.1825

Figure 8H

migration				invasion		
0μM	2μM	4μM		0μM	2μM	4μM
0.903958	0.649077	0.424274		1.033019	0.673181	0.38814
1.074934	0.595251	0.326121		0.994609	0.640836	0.317385
1.021108	0.763061	0.465435		0.972372	0.570081	0.291105

Figure 8J

0μM	2μM	4μM
0.395195	0.29297	0.257213
0.390188	0.290738	0.230352
0.365045	0.342198	0.225037

Figure 8K

0μM	2μM	4μM
8.28	13.31	20.01
7.62	14.54	19.78
9.89	15.63	18.92

Figure 8O

time(h)	DMSO			CAS-12290-201		
0	1	1	1	1	1	1
4	0.75491	0.835656	0.732212	0.530705	0.456872	0.551014
8	0.644201	0.658775	0.583063	0.321987	0.266322	0.33422
12	0.471236	0.428719	0.337752	0.056406	0.043532	0.089373

Figure 9A

AsPC-1	GEM			GEM+CAS-12290-201		
concentration(nM)	Viability			Viability		
0	1	1	1	1	1	1
20	1.04295	0.962206	0.984208	0.987603	0.908276	0.897341
40	0.996195	0.921075	1.019982	0.811368	0.737611	0.717426
60	0.981581	0.907992	0.934203	0.5342	0.458527	0.441647
80	0.91981	0.829717	0.89815	0.346433	0.286893	0.259389
100	0.828173	0.721124	0.78919	0.227654	0.181257	0.165833
120	0.642941	0.565957	0.623724	0.14793	0.102873	0.078431
140	0.43663	0.361501	0.41395	0.103854	0.055993	0.035365
160	0.294182	0.227595	0.268797	0.065436	0.021201	0.012035

Figure 9B

AsPC-1						
GEM(nM)\CAS(μM)	0	1	2	4	10	20
0	100.00%	89.44%	78.12%	64.44%	53.45%	48.33%
20	98.20%	83.81%	72.86%	58.88%	49.61%	44.63%
50	93.79%	71.53%	51.54%	35.78%	28.16%	25.10%
100	79.61%	53.49%	24.19%	13.79%	10.40%	9.21%
300	25.73%	20.62%	14.90%	9.70%	8.33%	7.87%
1000	15.36%	13.29%	8.78%	4.15%	3.87%	3.31%

Figure 9C

PANC-1	GEM			GEM+CAS-12290-201		
concentration(nM)	Viability			Viability		
0	1	1	1	1	1	1
20	0.988173	1.065763	0.946788	0.913287	0.95698	0.879068
40	0.896731	0.982601	0.874335	0.604114	0.640695	0.557938
60	0.762153	0.857897	0.740443	0.311231	0.333679	0.24307
80	0.587696	0.68809	0.557043	0.164751	0.204266	0.126814
100	0.426397	0.475433	0.392564	0.095841	0.134537	0.065722
120	0.312301	0.382469	0.306644	0.067128	0.097111	0.033241
140	0.221871	0.283856	0.203354	0.041054	0.076615	0.02242

Figure 9D

PANC-1						
GEM(nM)\CAS(μM)	0	1	2	4	10	20
0	100.00%	91.15%	76.99%	62.70%	52.53%	46.11%
20	102.28%	87.42%	63.25%	57.89%	48.62%	42.33%
50	85.16%	68.28%	38.74%	31.55%	28.67%	23.14%
100	45.51%	32.97%	20.81%	13.12%	11.46%	9.56%
300	23.00%	18.41%	11.15%	9.53%	8.87%	6.47%
1000	15.90%	11.80%	7.43%	4.17%	3.10%	2.26%

Figure 9F

DMSO	DMSO+CAS	GEM50	GEM50+CAS	GEM100	GEM100+CAS
3.80%	6.63%	18.54%	53.98%	46.33%	73.22%
4.35%	8.80%	19.78%	51.14%	43.44%	78.38%
5.25%	8.59%	17.44%	57.73%	47.47%	74.98%

Figure 9G

DMSO	GEM	GEM+CAS
0.421053	0.286408	0.180693069
0.403599	0.28246	0.160714286
0.448454	0.269417	0.120320856

Figure 9J

DMSO	GEM	GEM+CAS
0.403	0.2225	0.0499
0.3848	0.1964	0.0358
0.3065	0.1876	0.0476
0.2544	0.2284	0.043
0.2237	0.1573	0.024
0.2349	0.1316	0.0235

Figure 9K

weeks	vehicle							weeks	GEM					
2	124.4803	80.42063	124.6266	68.74903	68.12134	82.16934		2	58.74192	63.35941	51.01835	51.79995	37.08812	34.69195
3	194.7211	128.341	225.5802	111.9998	133.0462	127.0516		3	115.516	97.80443	80.64159	91.19268	67.15661	54.55722
4	373.7701	210.8195	355.2394	191.1404	191.2205	220.3776		4	179.4269	164.5151	135.8021	128.7291	99.63072	100.3223
5	451.5863	329.4662	467.8577	288.2058	289.206	301.1124		5	280.0526	224.8994	203.2347	190.5042	178.024	130.0735
6	659.6389	412.6611	677.9633	348.4138	383.6042	430.3805		6	363.428	315.8508	250.6523	270.6969	218.1393	183.4104

weeks	GEM+CAS					
2	29.25279	31.04665	29.54661	25.08878	17.61457	12.4656
3	40.87502	46.49157	43.40981	40.28766	25.8935	19.99488
4	47.89396	47.91066	54.55109	47.72651	30.20744	24.0216
5	46.60687	51.33983	58.14662	52.40318	34.37661	25.31993
6	54.76666	58.06246	56.8146	54.20461	35.99411	26.6328

Figure 9L

Vehicle	GEM	GEM+CAS
66.99%	44.67%	18.95%
57.56%	39.86%	17.48%
51.02%	44.34%	16.63%
59.88%	43.87%	11.66%
53.68%	43.33%	15.35%
53.99%	41.65%	15.19%

Figure S2B

shControl	shVCPIP1
1.059387	0.158069
0.962708	0.126025
0.977905	0.115853

Figure S2C

shControl	shVCPIP1
1.191665	0.195971
0.933371	0.151225
0.892338	0.119813
1.280327	0.183744
0.834951	0.077244
0.867348	0.094684

Figure S3D

shControl	shVCPIP1#1	shVCPIP1#2
1.05058	1.06709987	0.98288083
1.035693	1.11816671	1.03698488
0.913727	0.93781441	0.97210937

Figure S3F

0	0.5	1.0
1.049194	0.98647211	0.9678603
0.895841	1.06247826	1.13229706
1.054965	1.18832291	0.93644302

Figure S3E

0	2	4
0.939537	0.98476441	1.14031317
0.962191	1.05622965	0.95584023
1.098272	0.97200063	1.07051509

Figure S4C

0	2	4
1.110651	1.032788	1.026438
0.930744	1.067499	1.131921
0.958605	0.923247	0.91399

Figure S4D

0	2	4
1.060594	0.983882	1.146441
0.985121	1.051612	0.96148
0.954284	0.940338	1.029576

Figure 2E

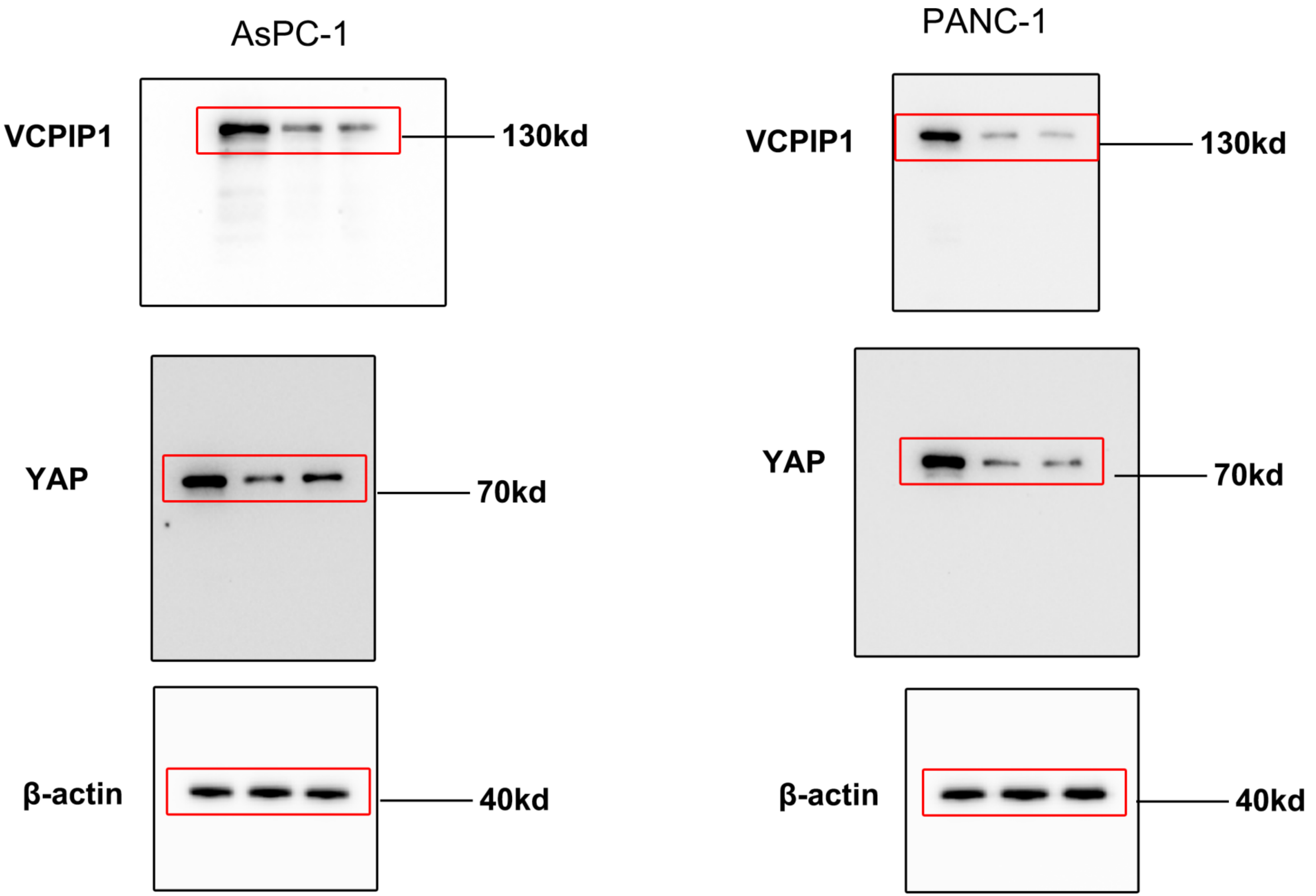

Figure 3E

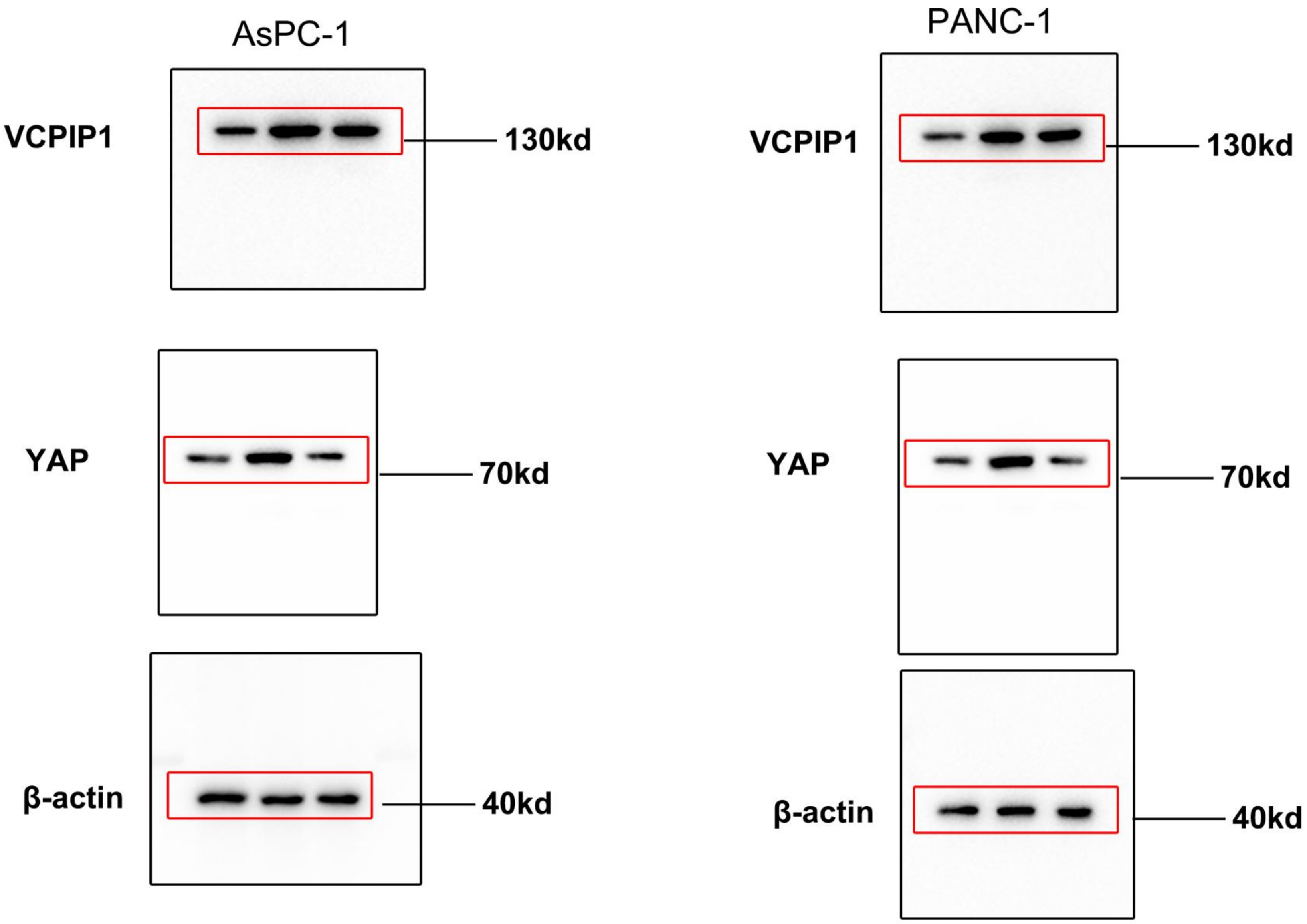
AsPC-1
PANC-1
VCPIP1
130kd
YAP
70kd
β-actin
40kd
VCPIP1
130kd
YAP
70kd
β-actin
40kd

Figure 4A

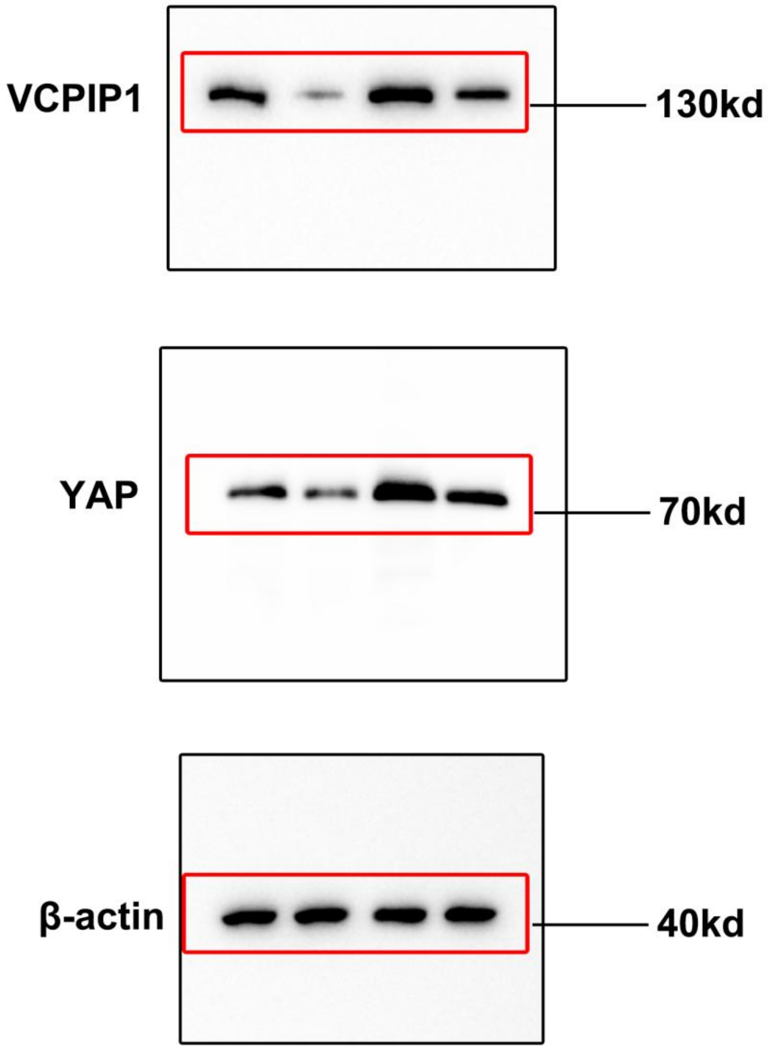

Figure 5D

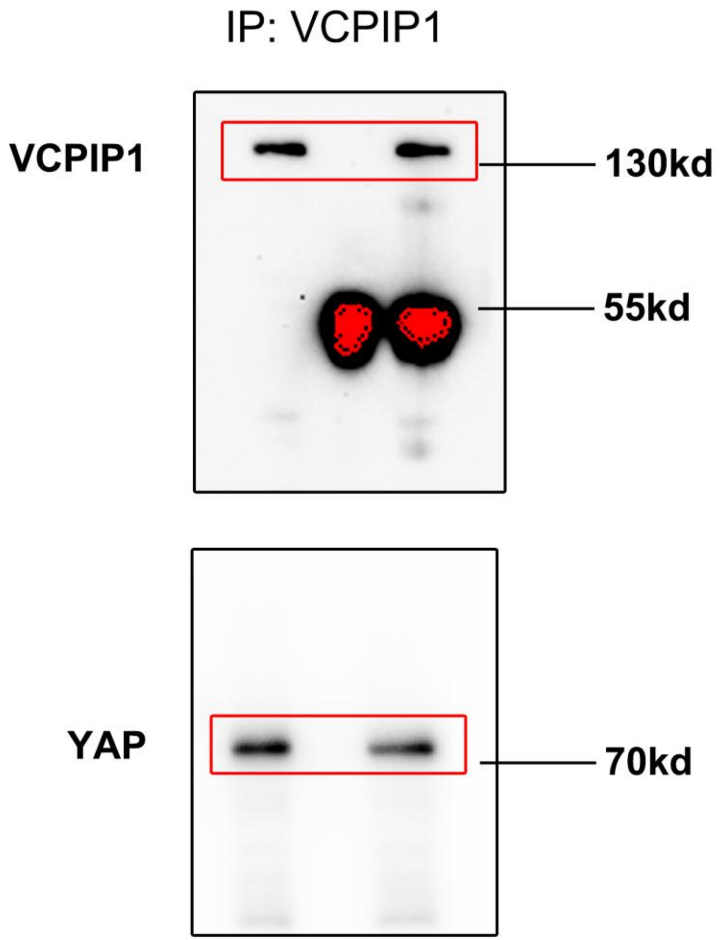

Figure 5E

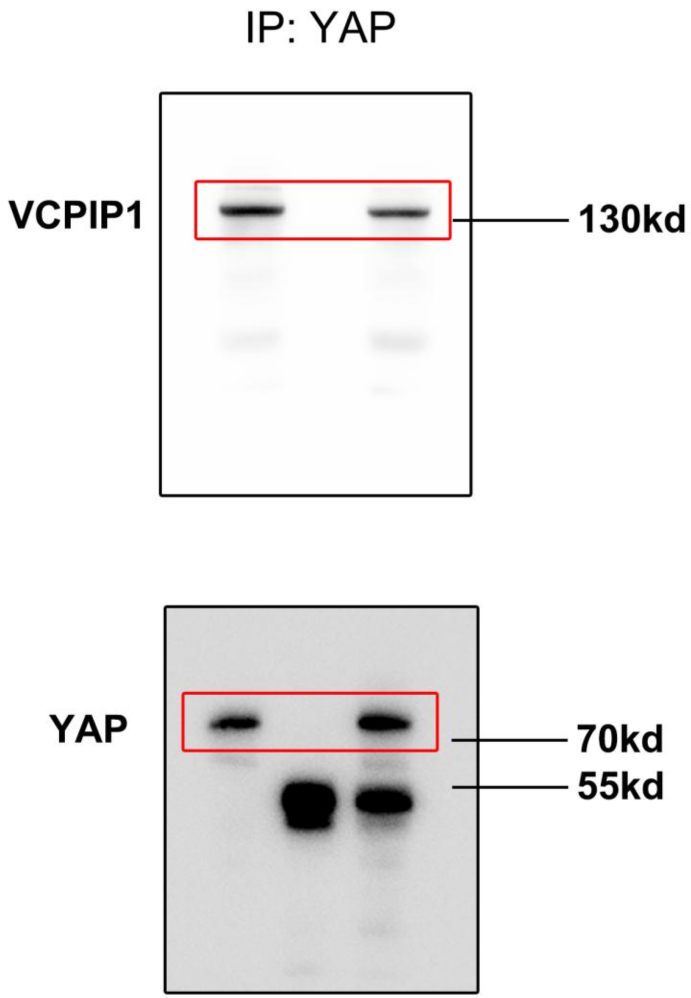

Figure 5G

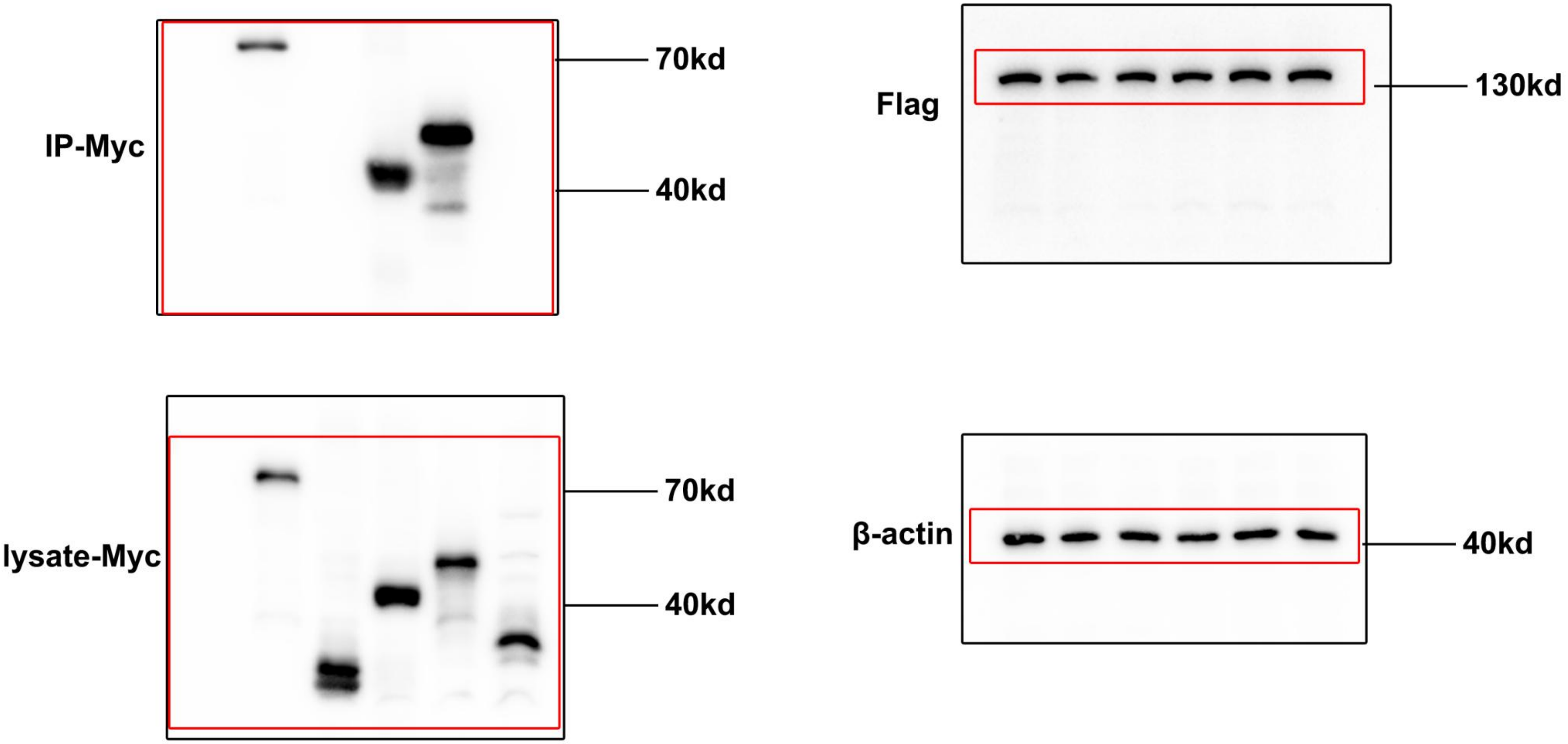

Figure 5H

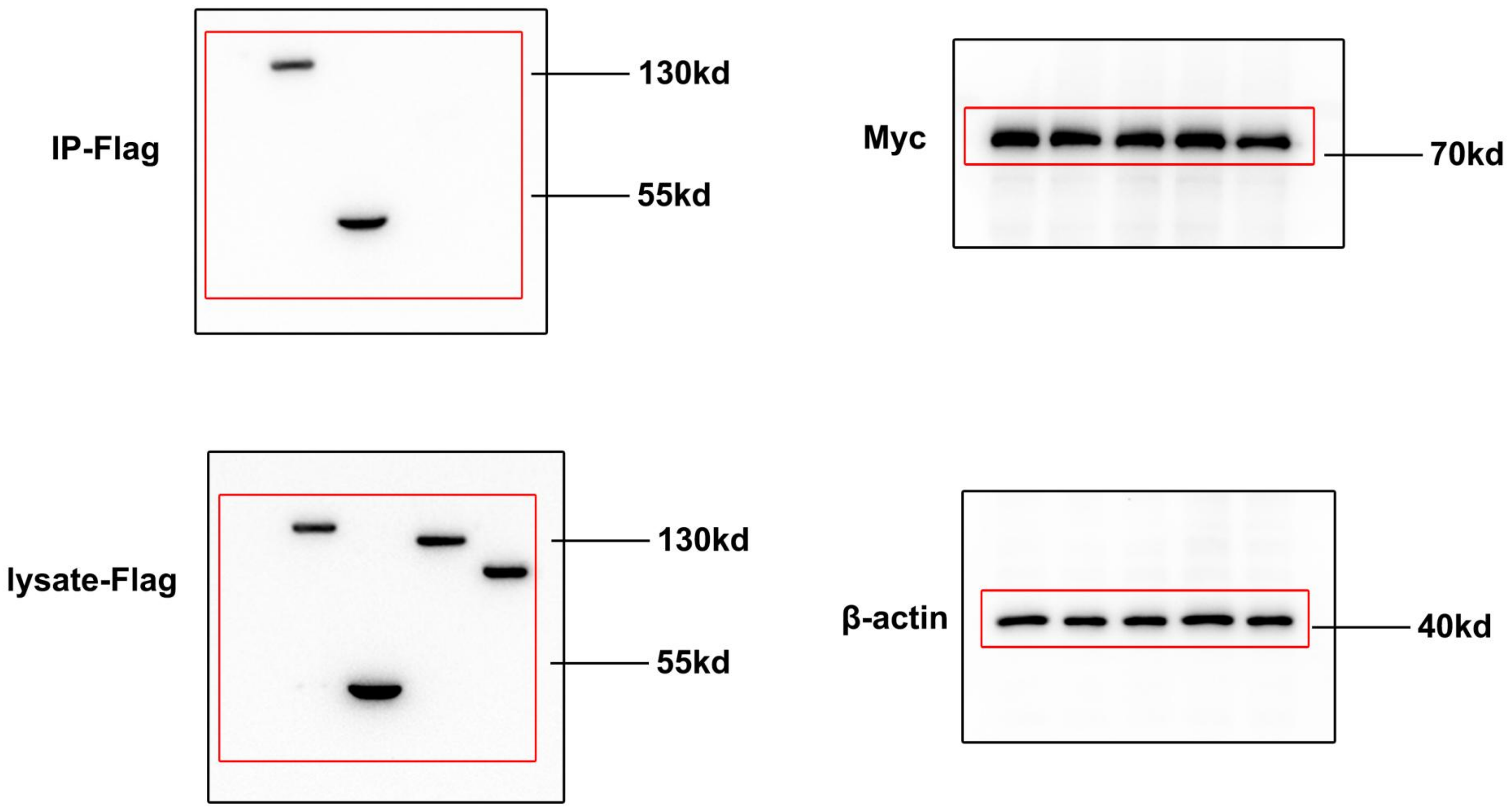
IP-Flag
130kd
55kd
Myc
70kd
lysate-Flag
130kd
55kd
β-actin
40kd

Figure 5J

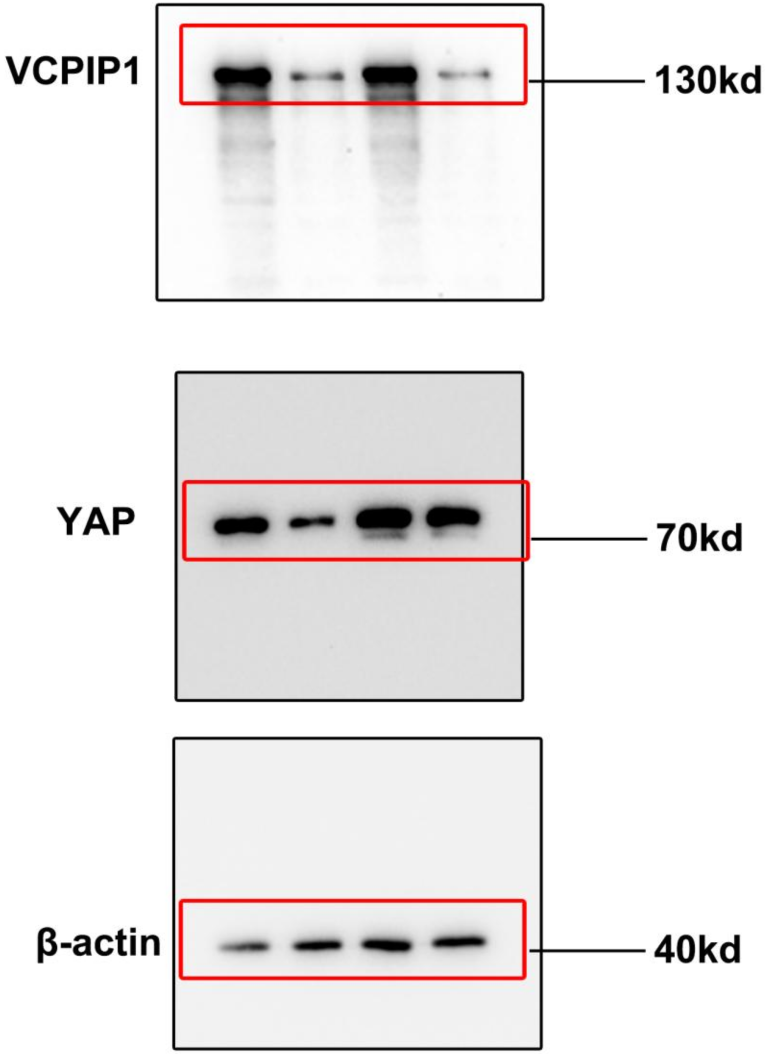

Figure 5K

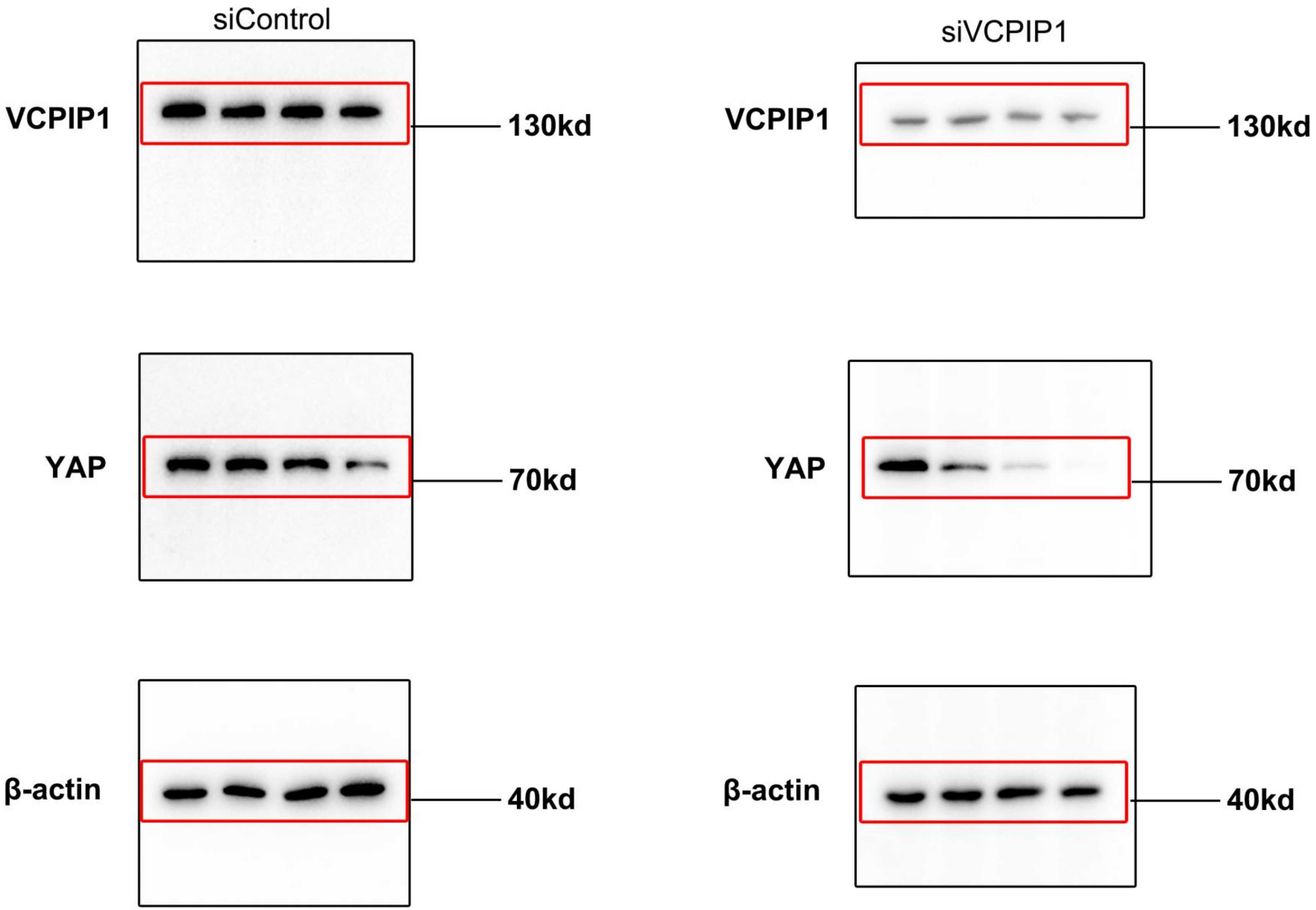

Figure 5M

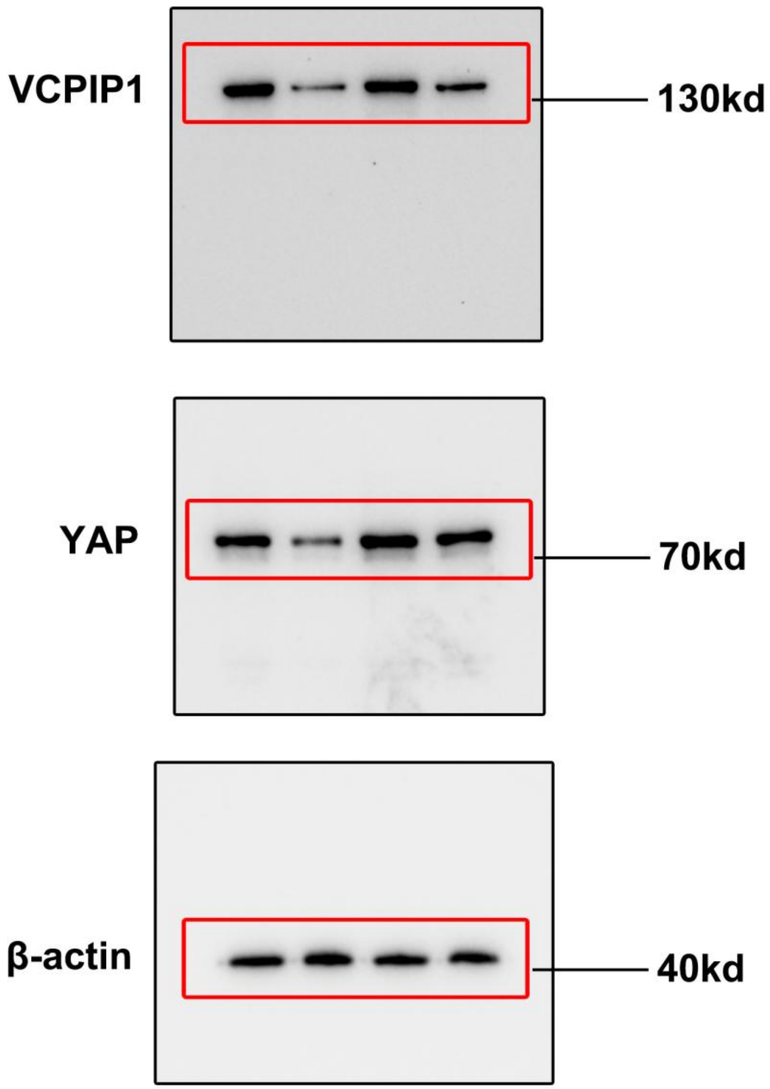

Figure 5N

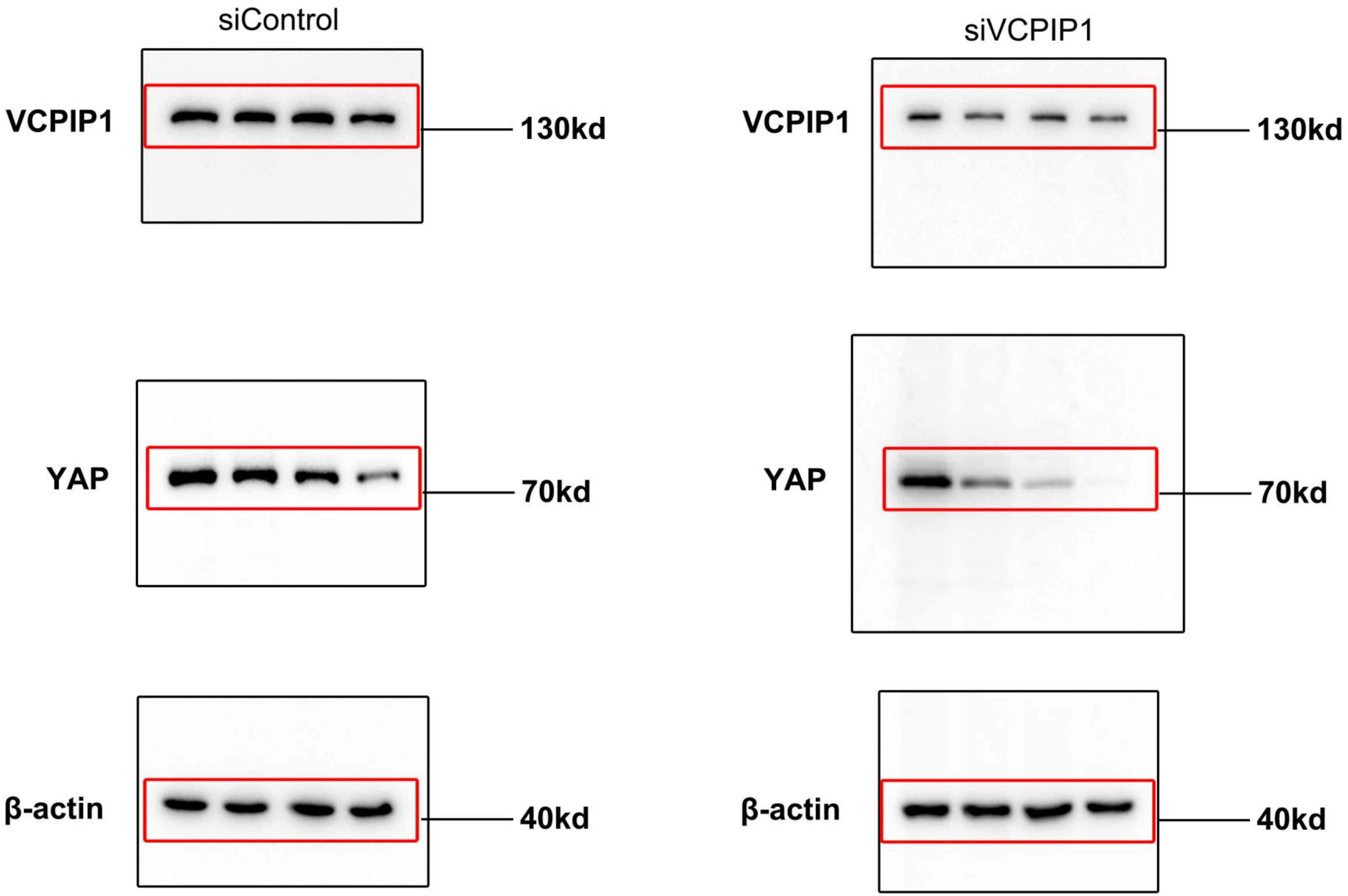
siControl
siVCPIP1
VCPIP1
130kd
VCPIP1
130kd
YAP
70kd
YAP
70kd
β-actin
40kd
β-actin
40kd

Figure 5P

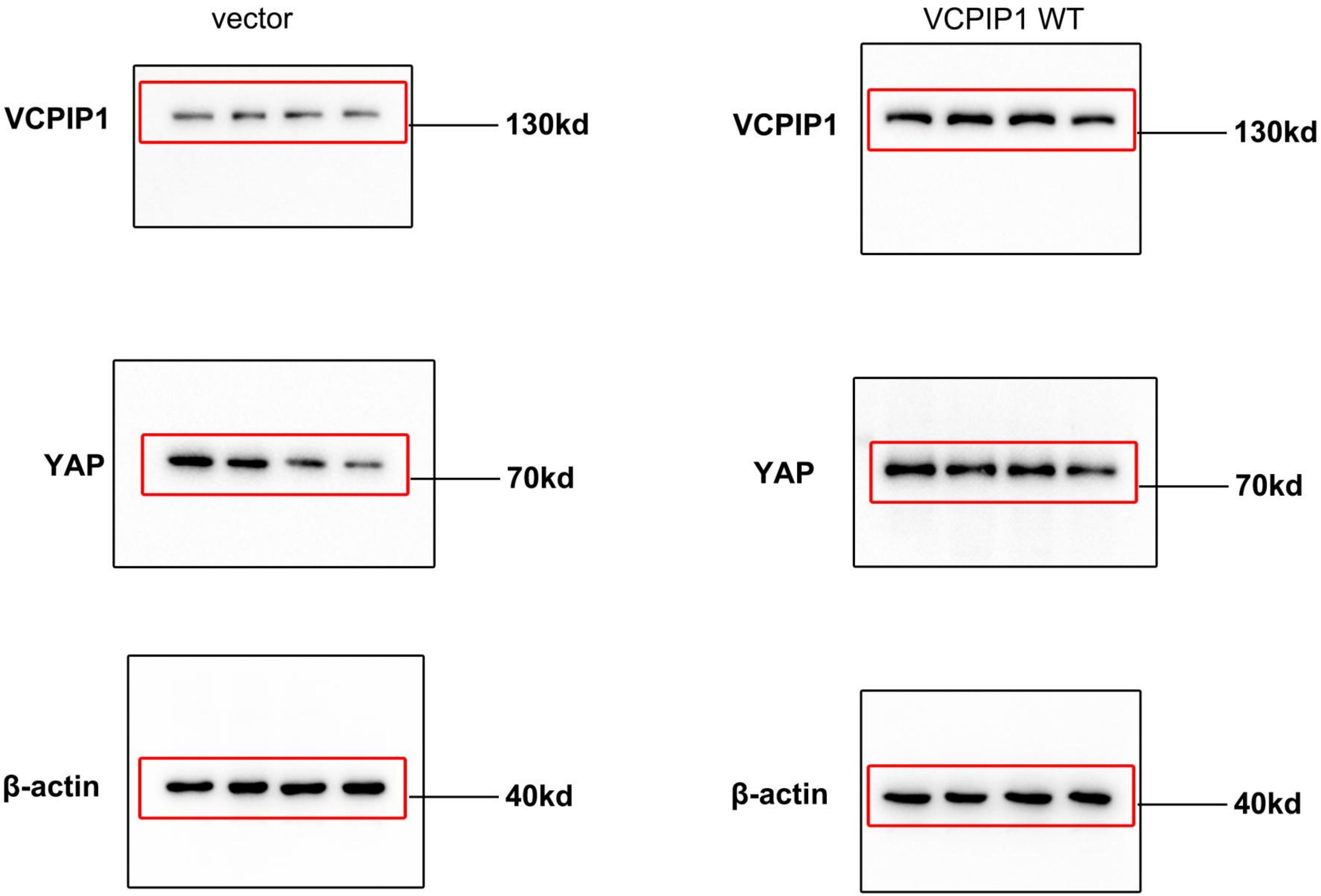
vector
VCPIP1 WT
VCPIP1
130kd
VCPIP1
130kd
YAP
70kd
YAP
70kd
β-actin
40kd
β-actin
40kd

Figure 5P

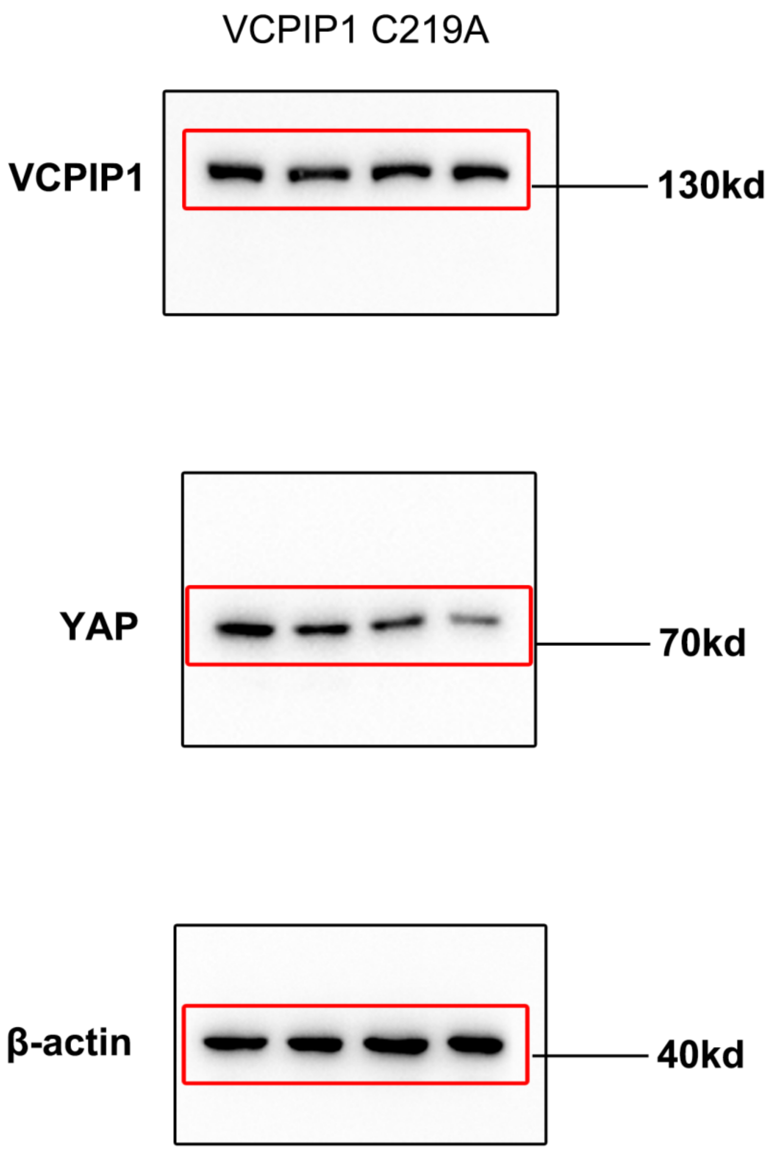

Figure 6A

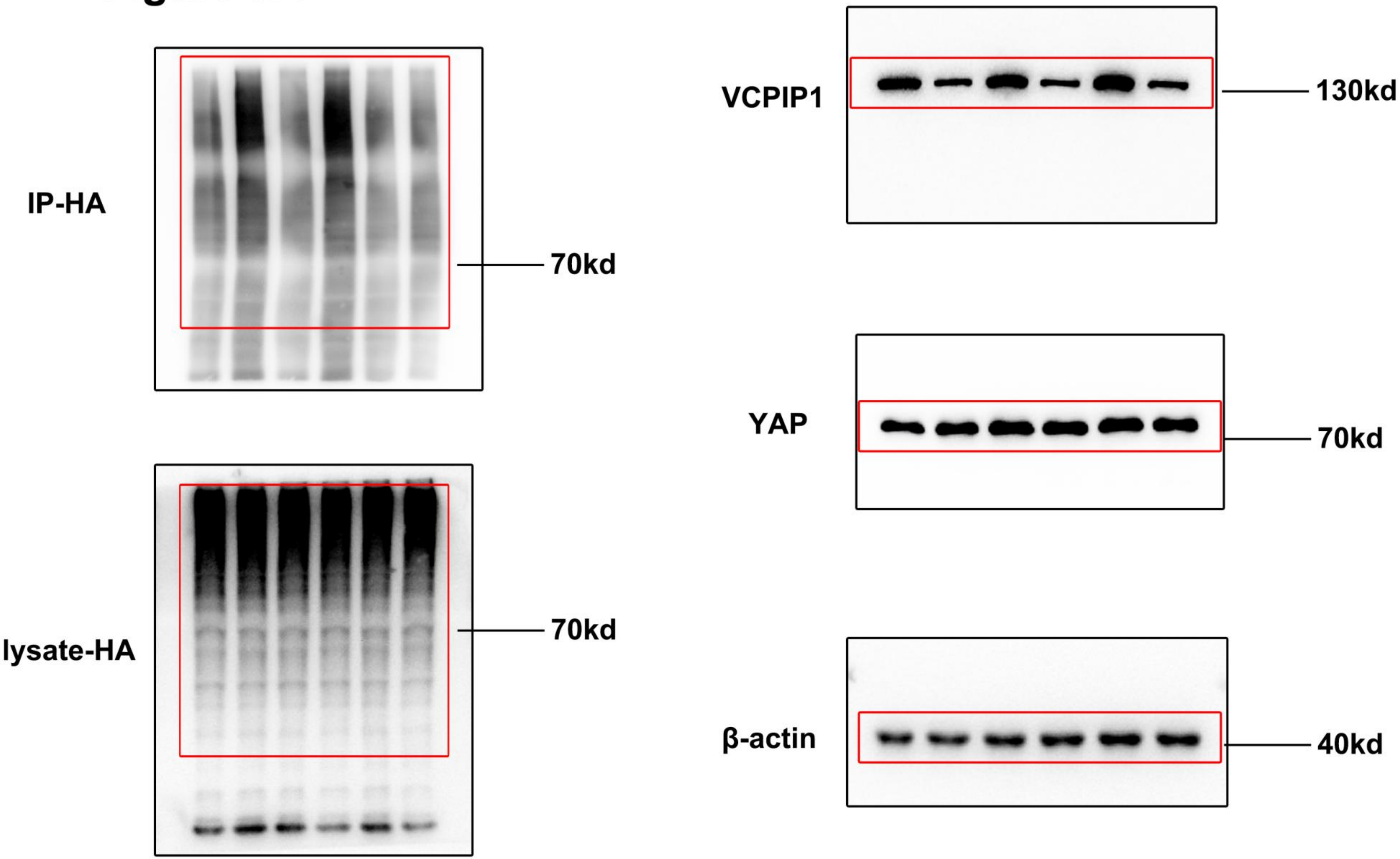

Figure 6B

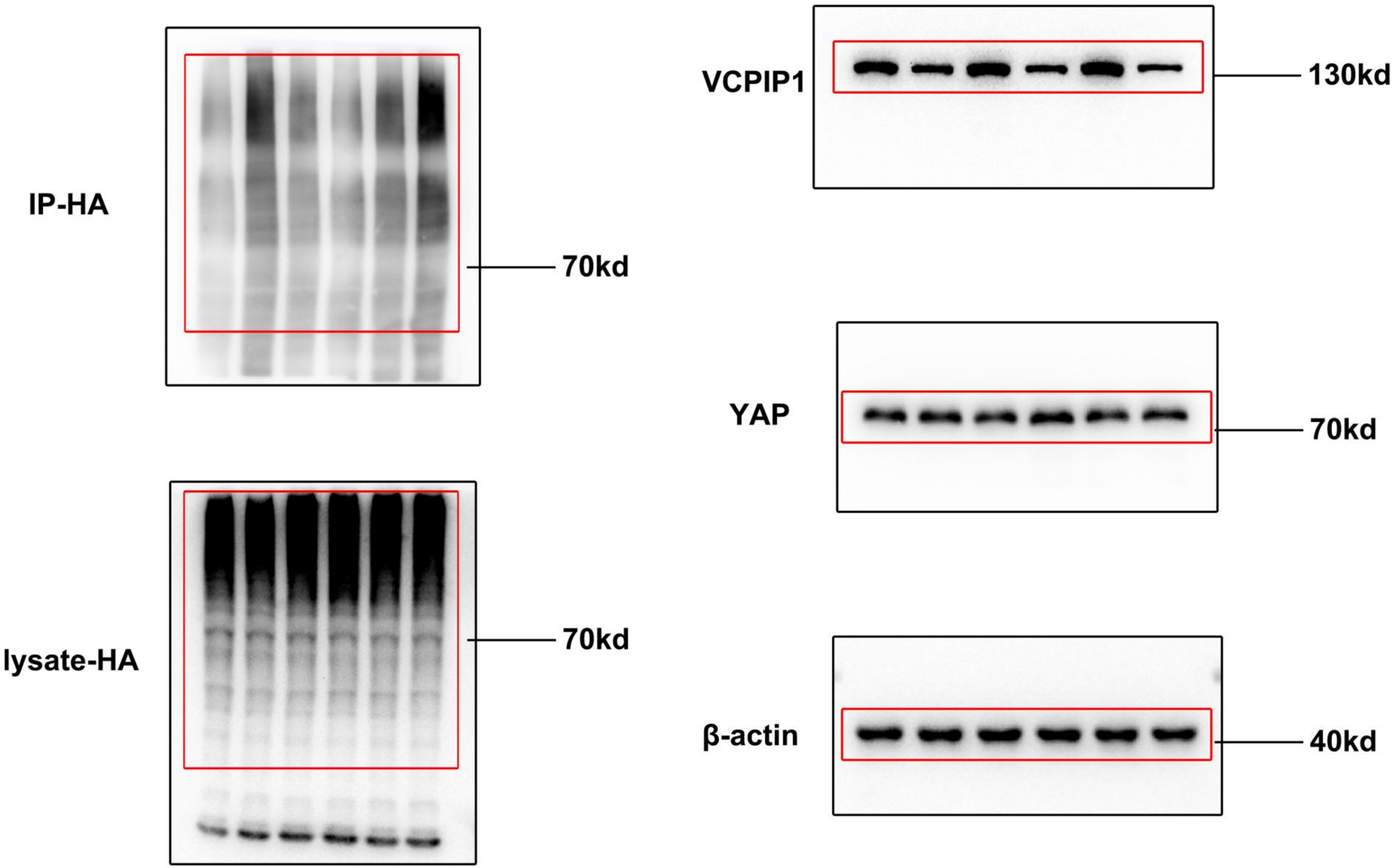

Figure 6C

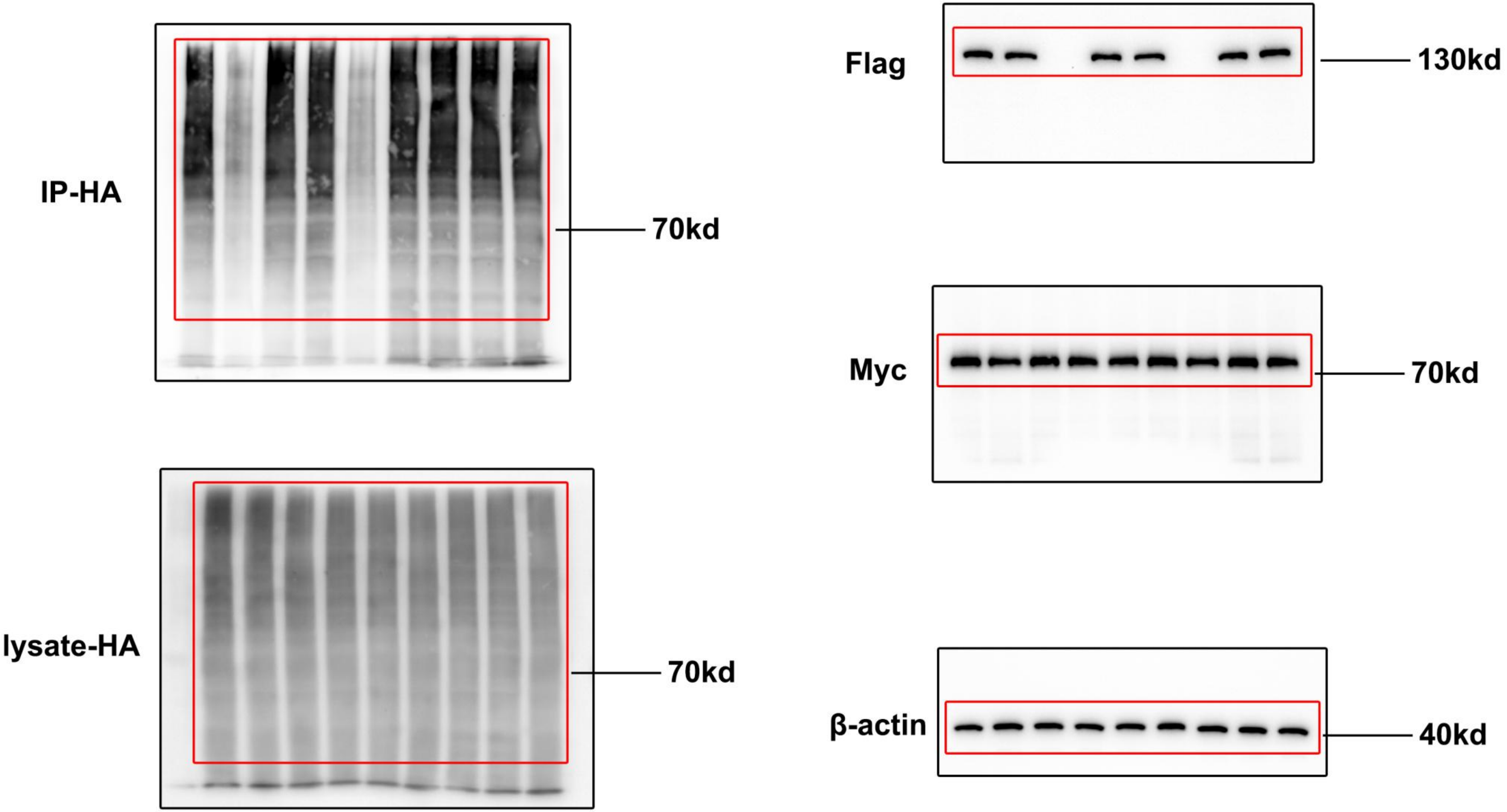

Figure 6D

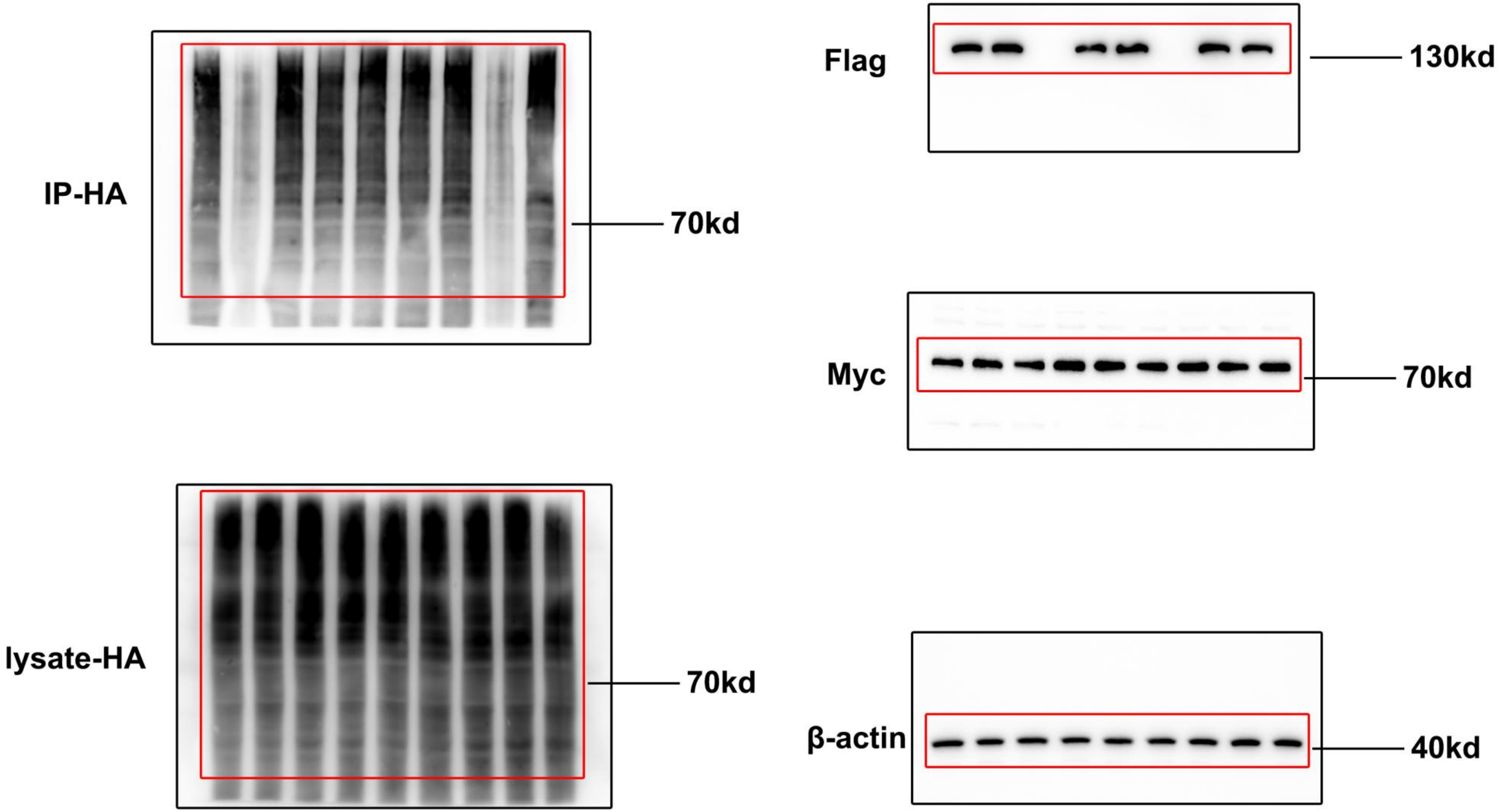

Figure 6E

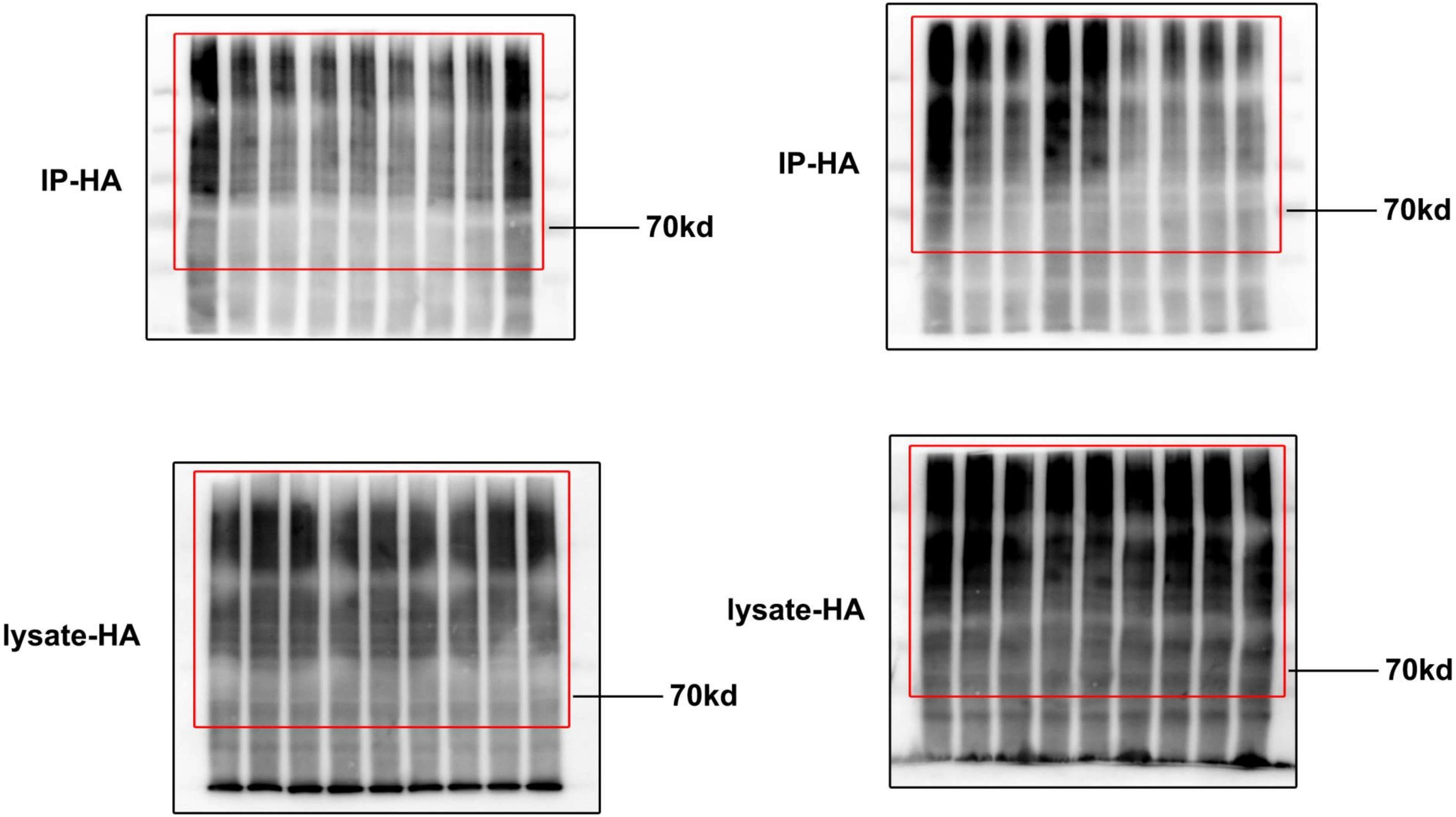

Figure 6E

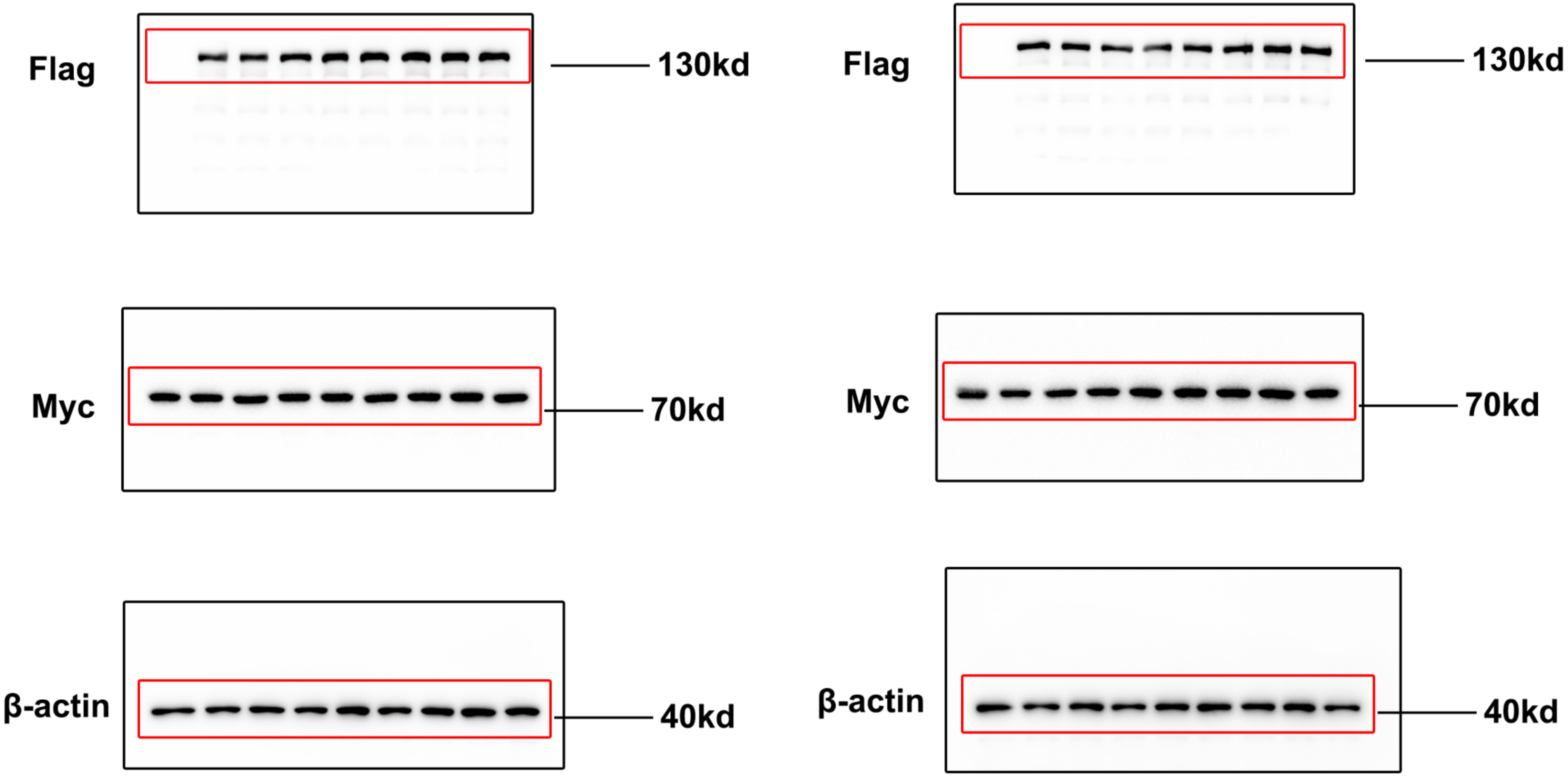

Flag
130kd
Myc
70kd
β-actin
40kd
Flag
130kd
Myc
70kd
β-actin
40kd

Figure 7F

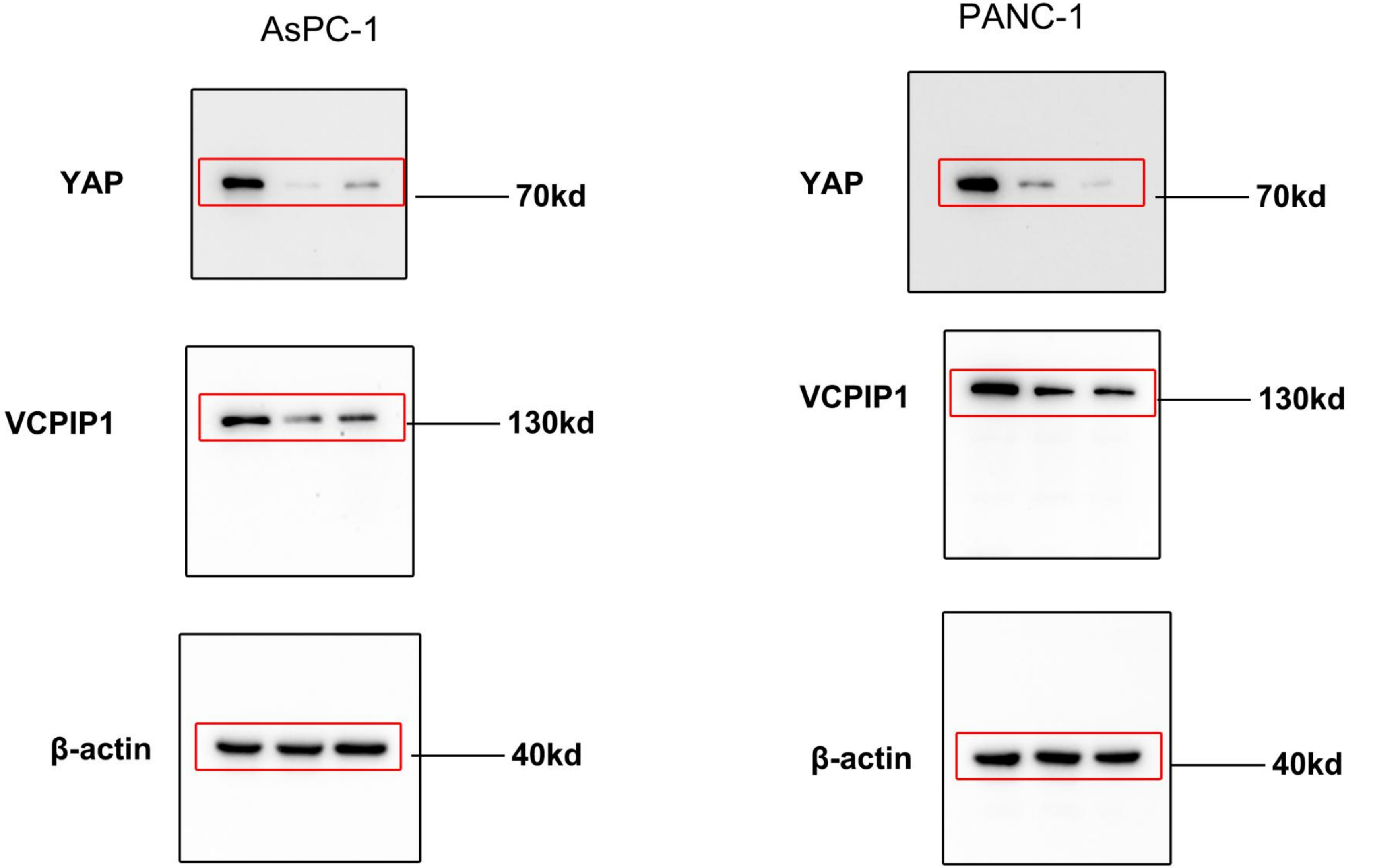

Figure 7I

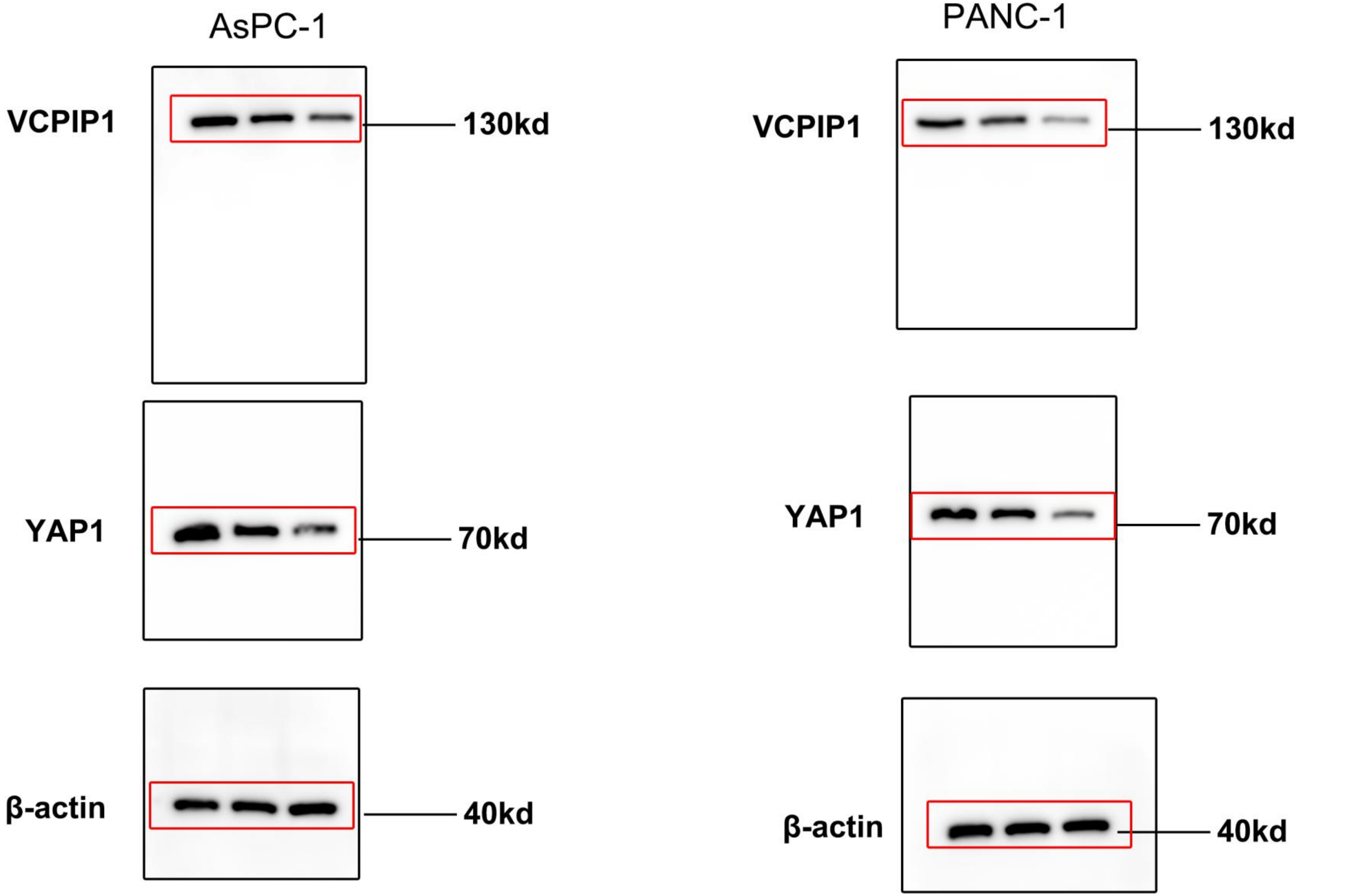
AsPC-1
PANC-1
VCPIP1
130kd
YAP1
70kd
β-actin
40kd
VCPIP1
130kd
YAP1
70kd
β-actin
40kd

Figure 7L

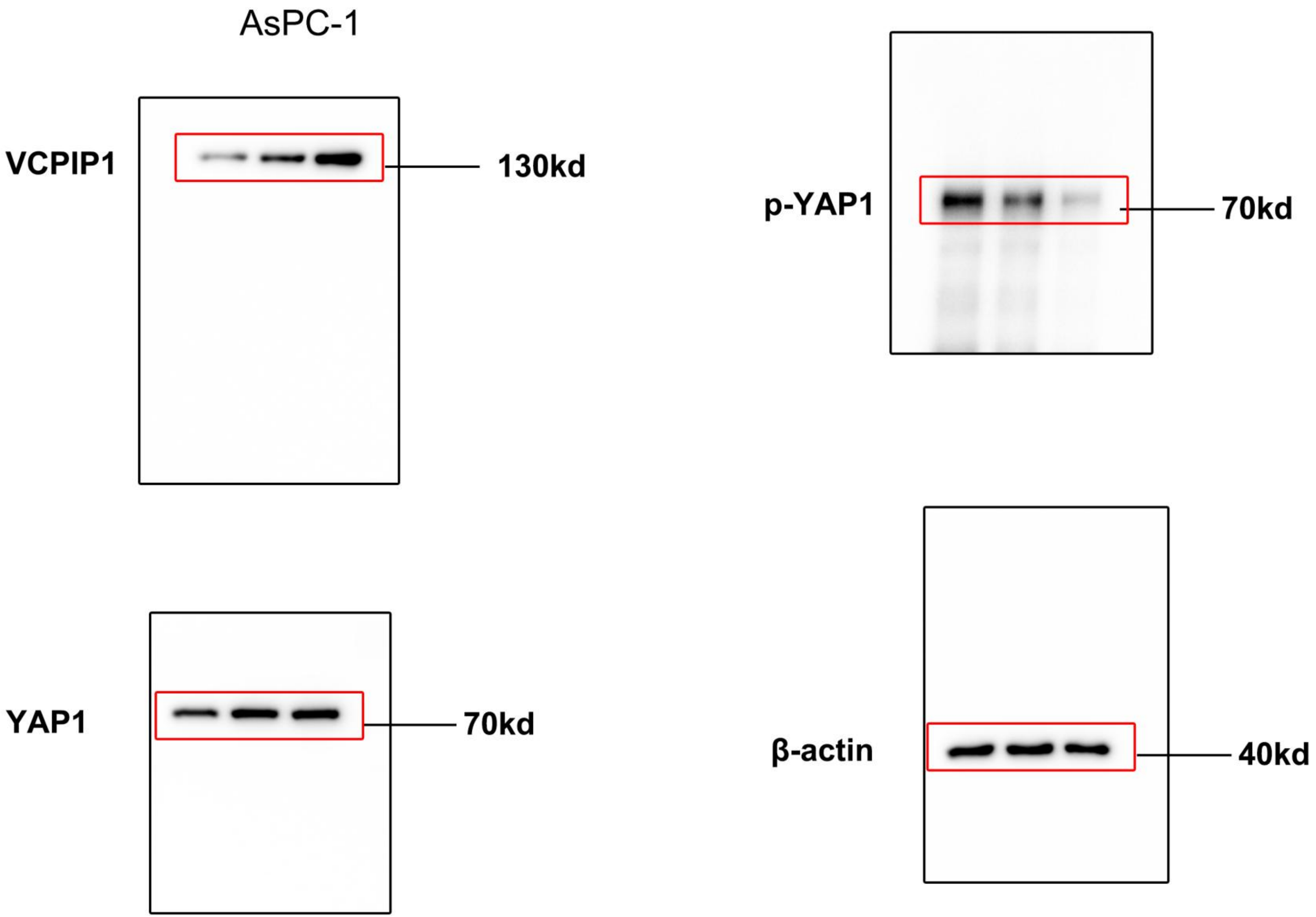

Figure 7L

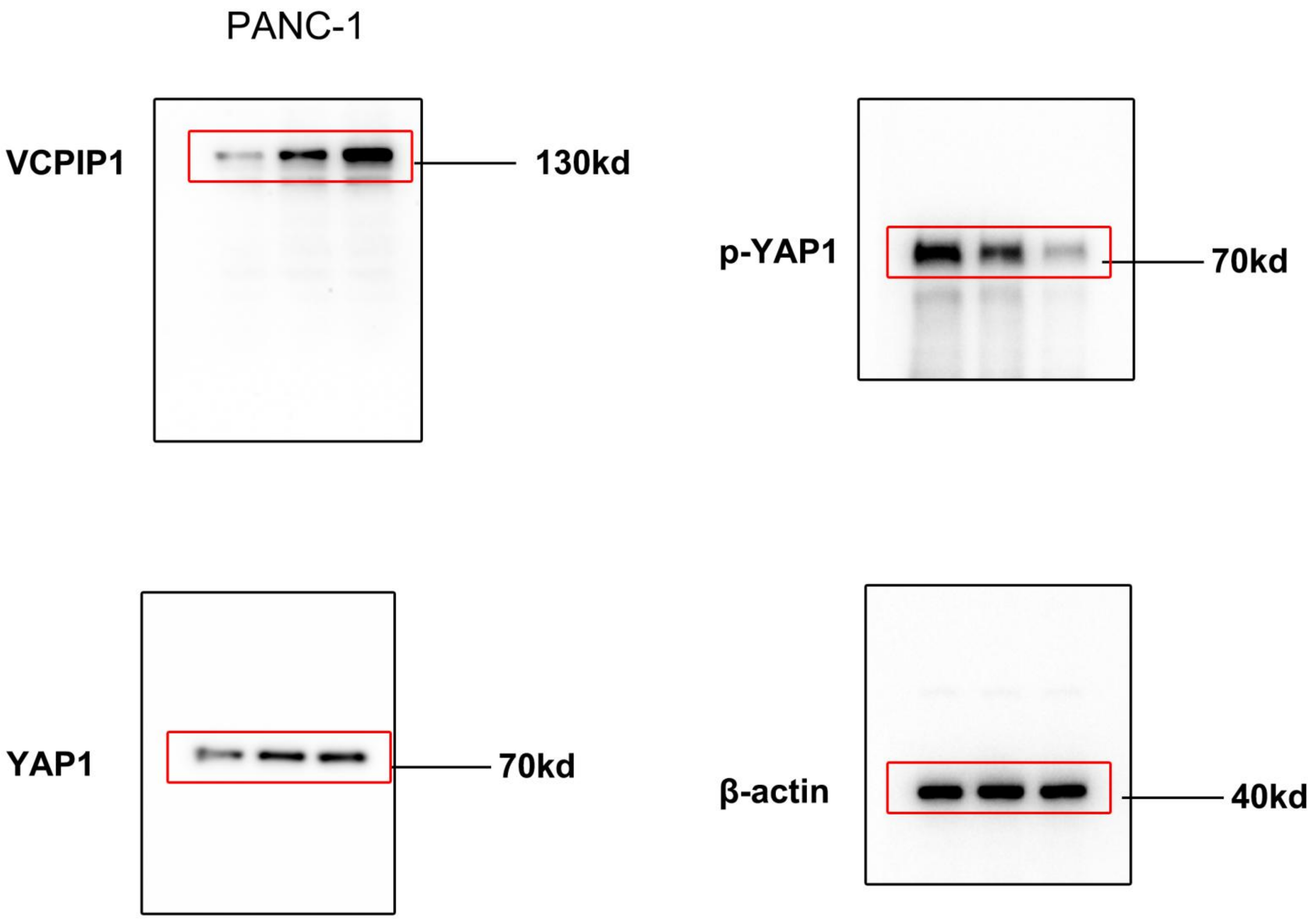

Figure 8B

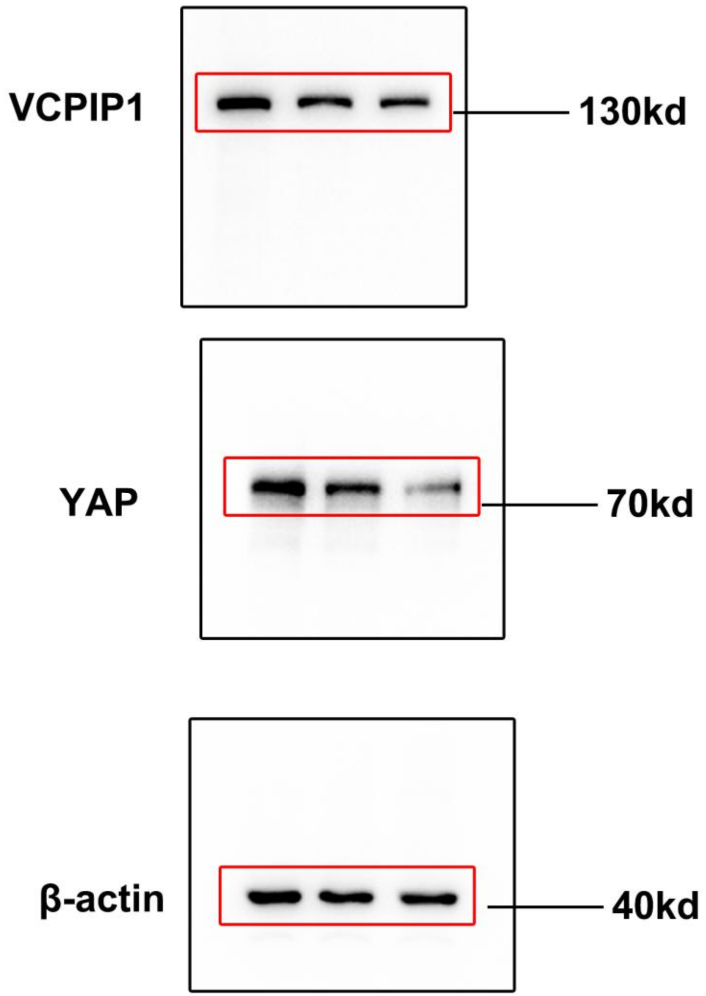

Figure 8L

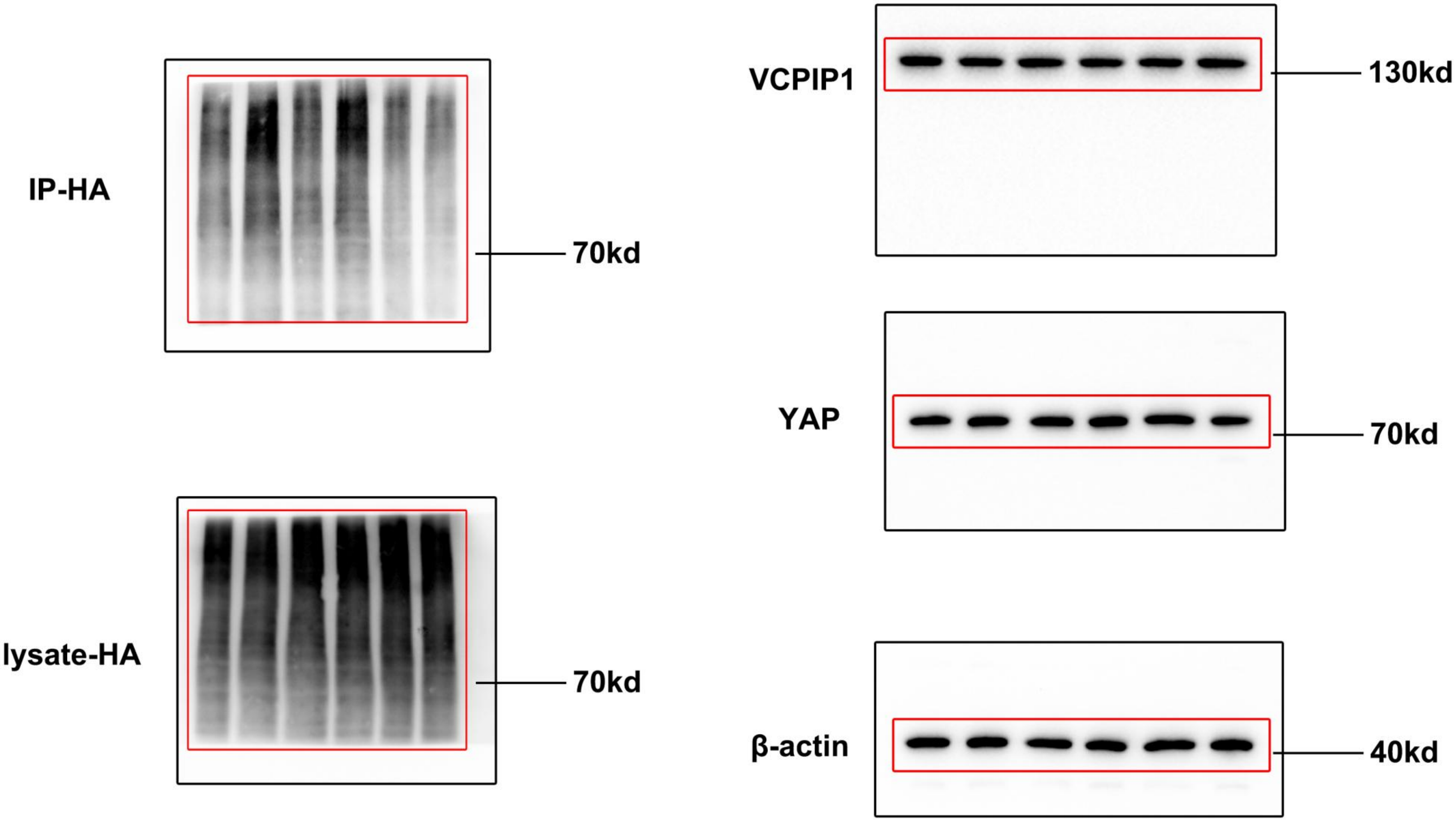

Figure 8M

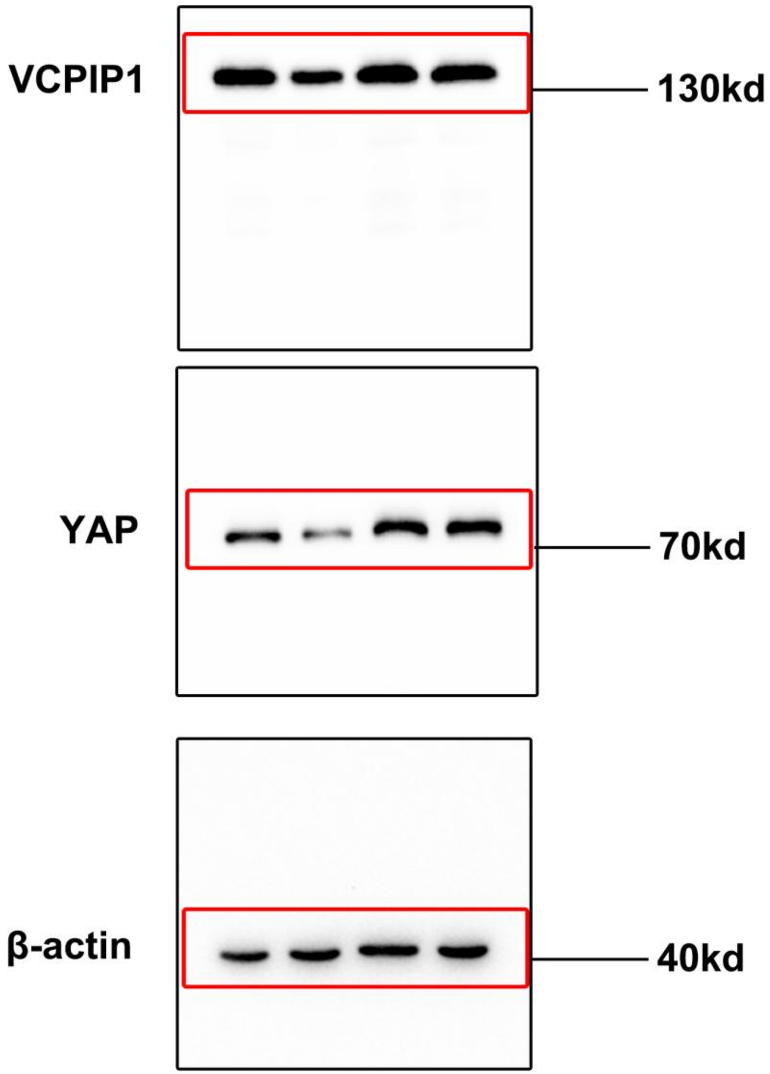

Figure 8N

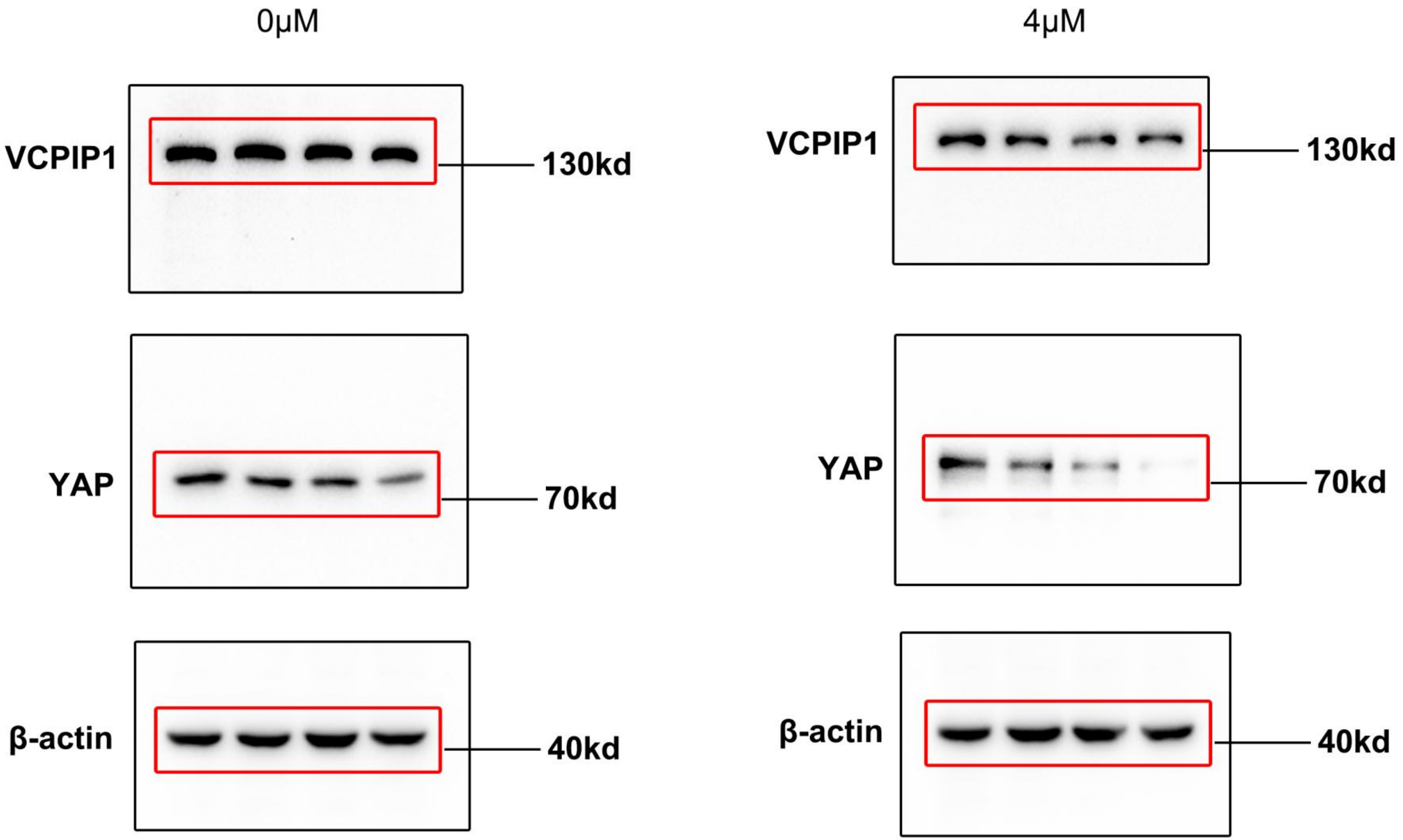

0μM
4μM
VCPIP1
130kd
YAP
70kd
β-actin
40kd
VCPIP1
130kd
YAP
70kd
β-actin
40kd

Figure S2A

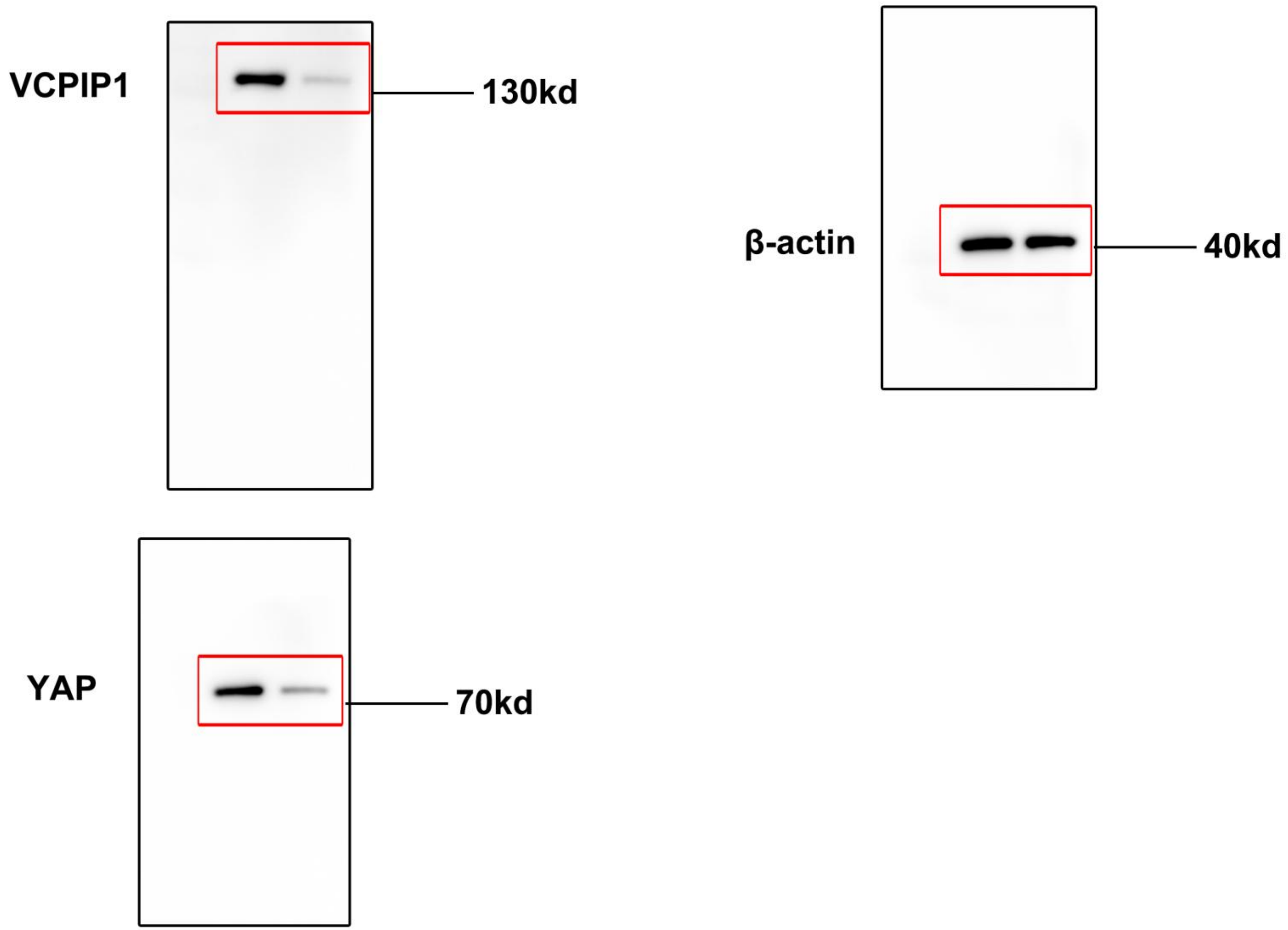

Figure S2D

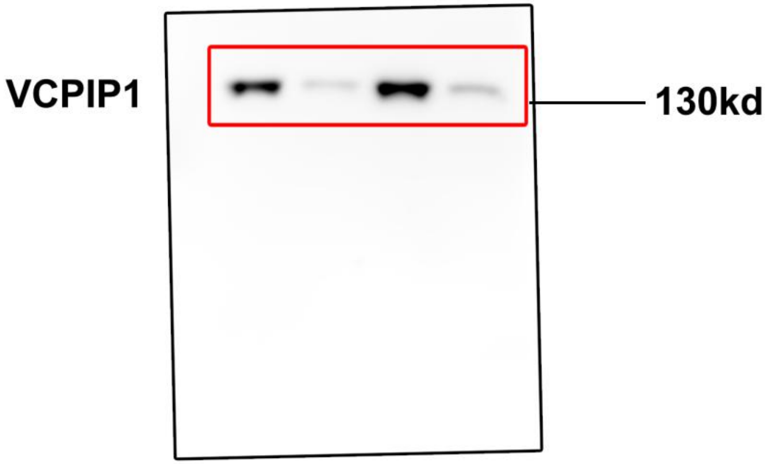

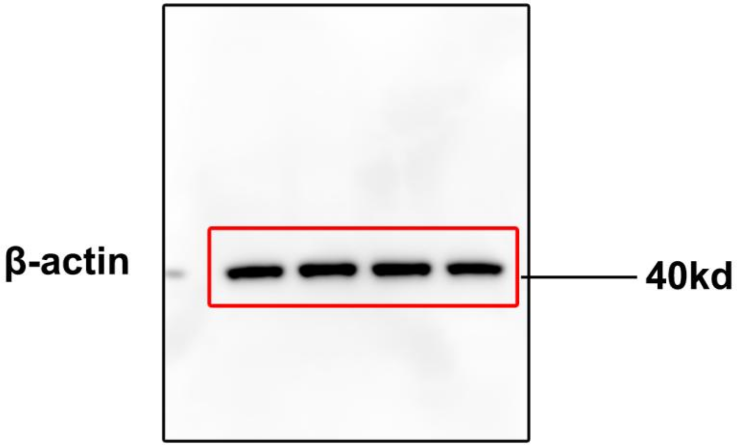

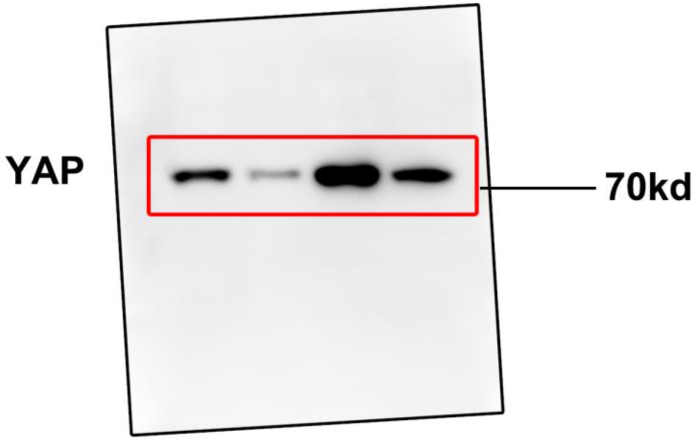

Figure S2E

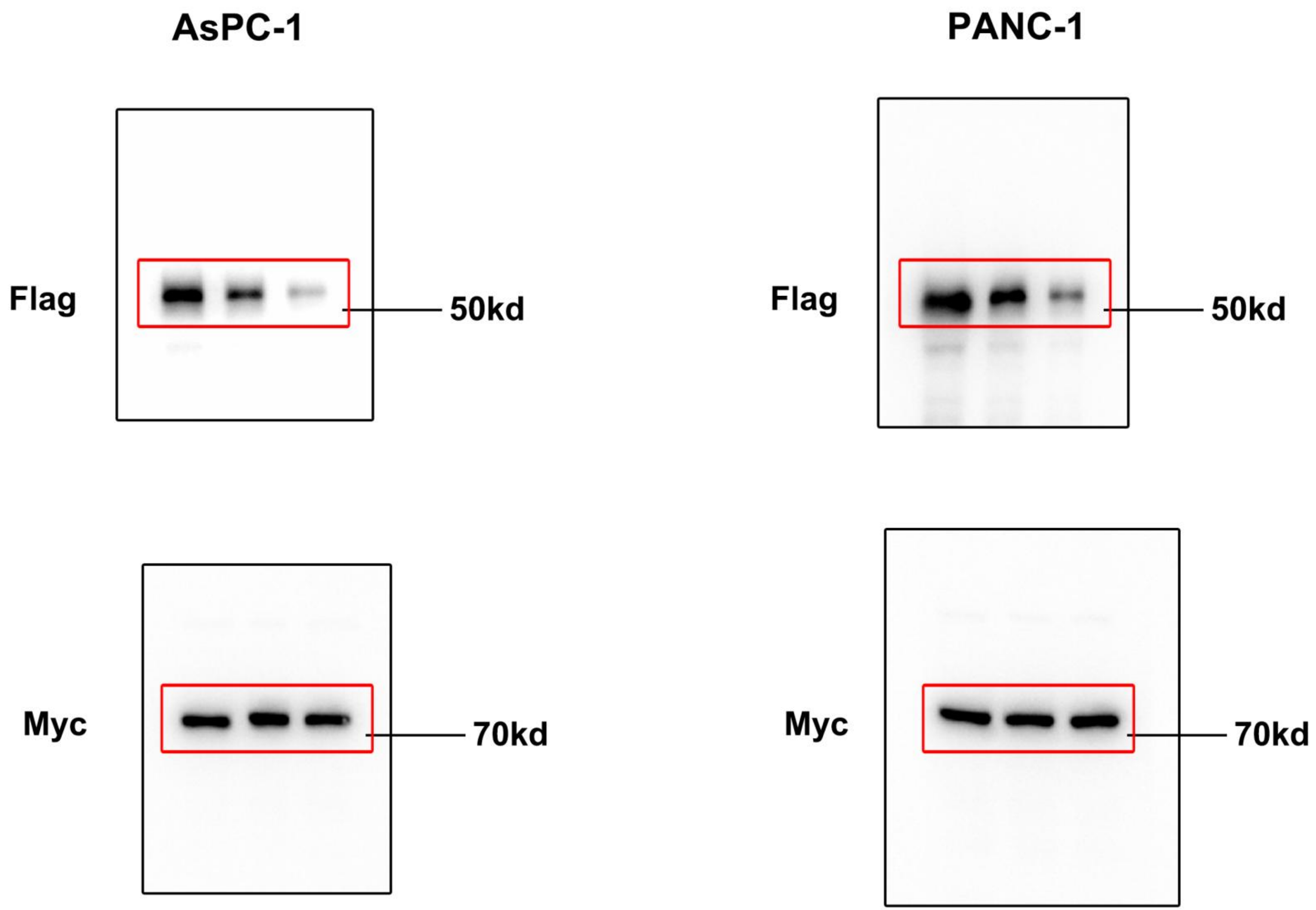

Figure S3A

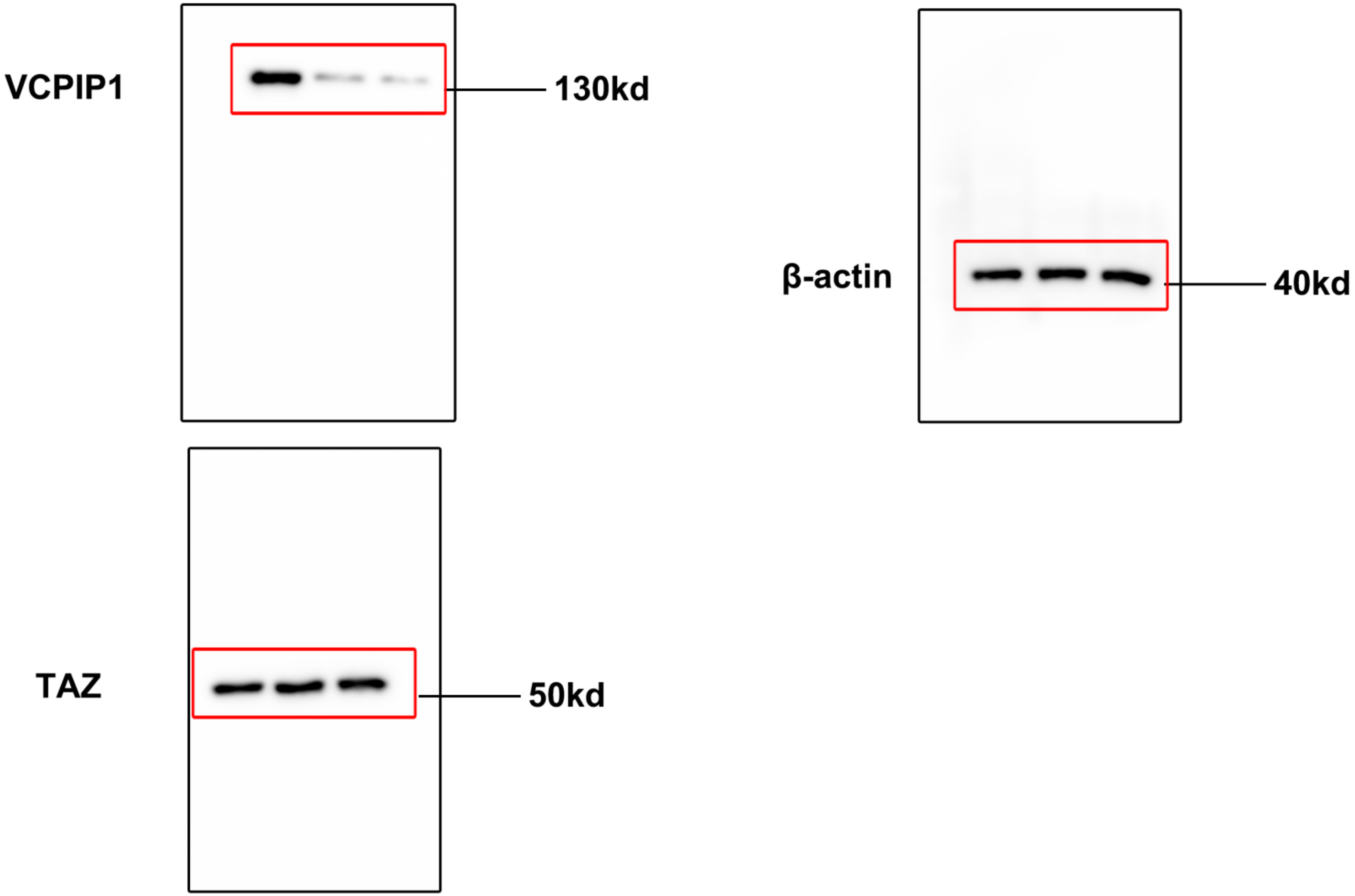

Figure S3B

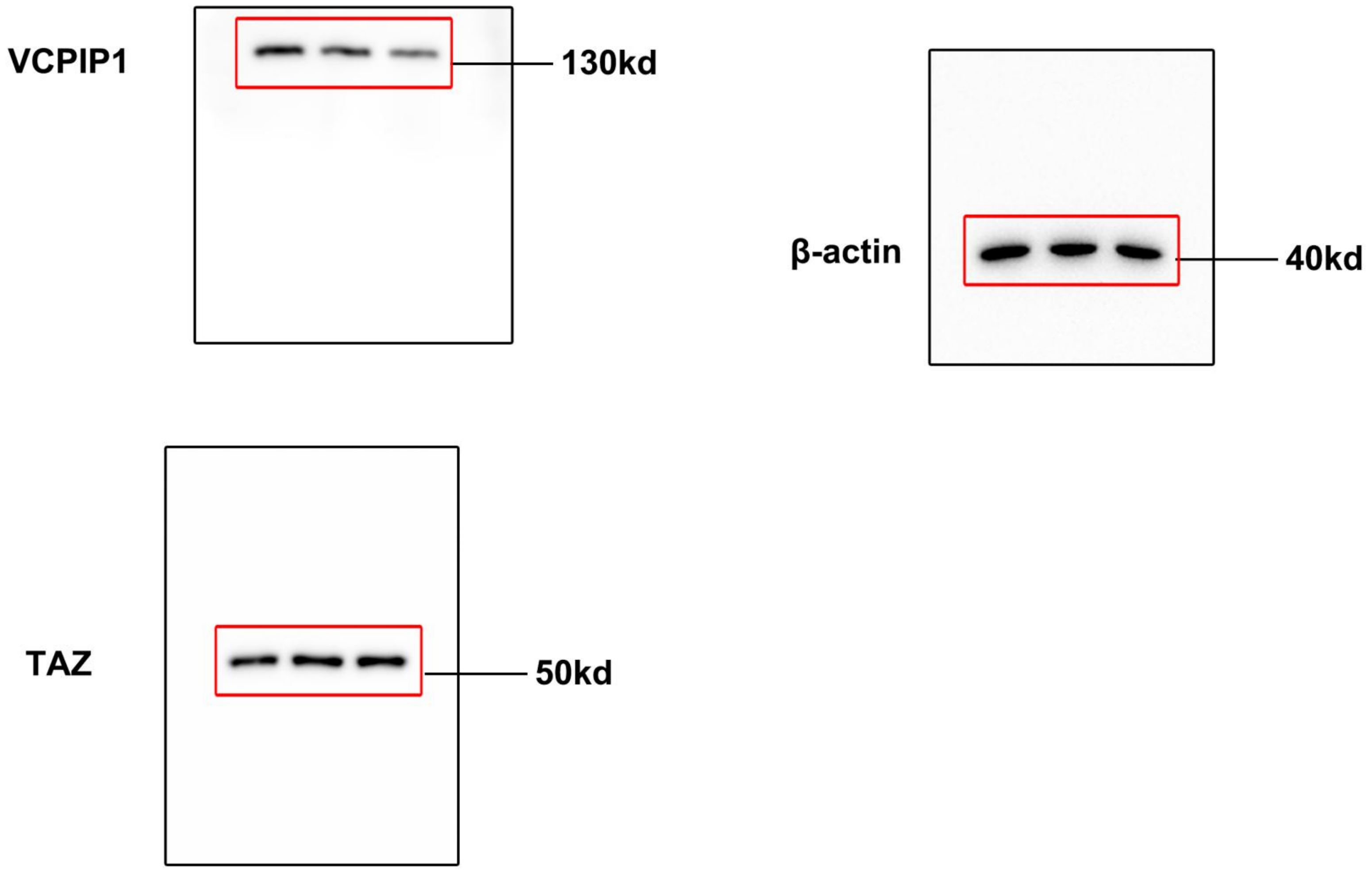

Figure S3C

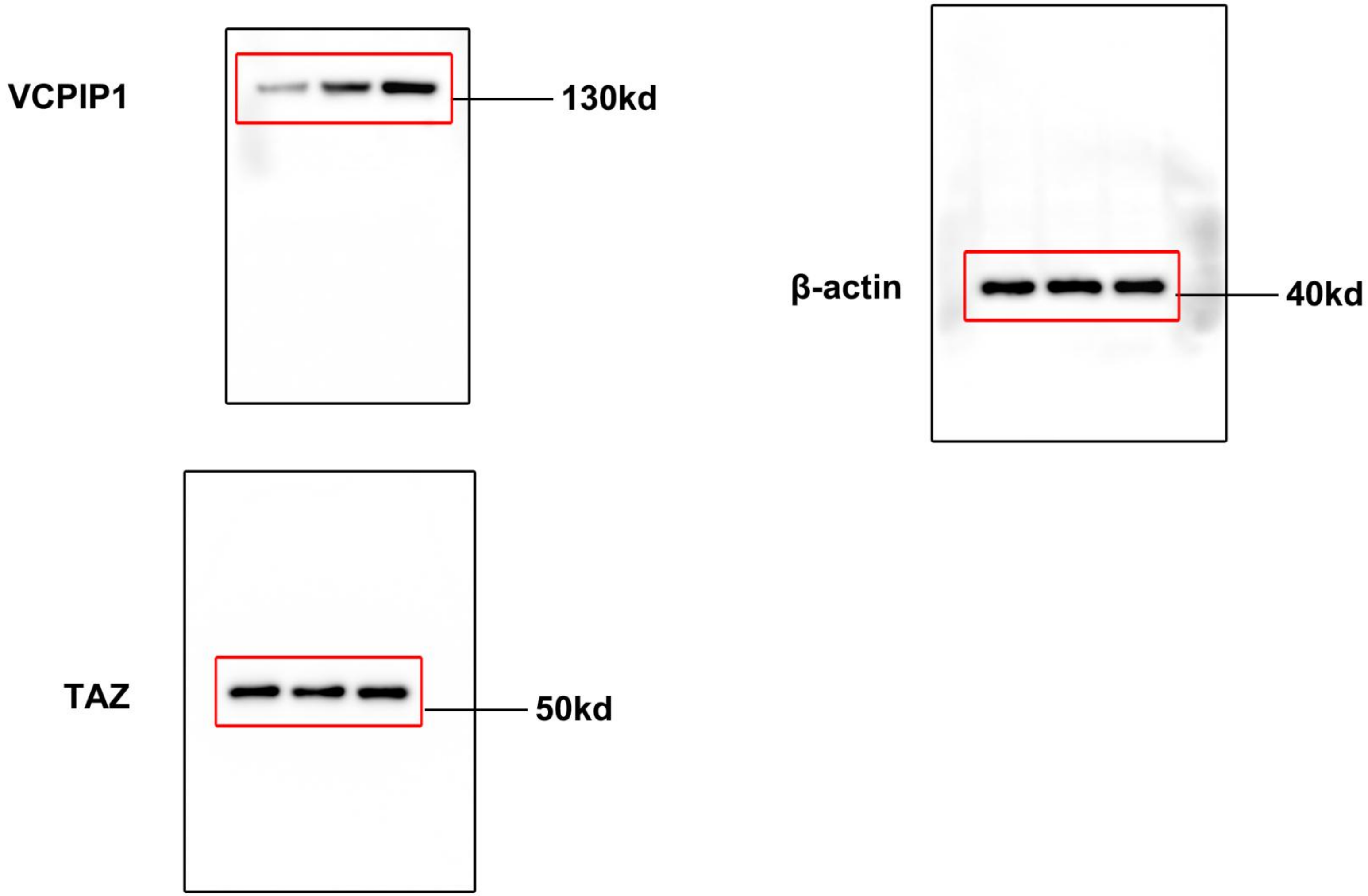

Figure S4A

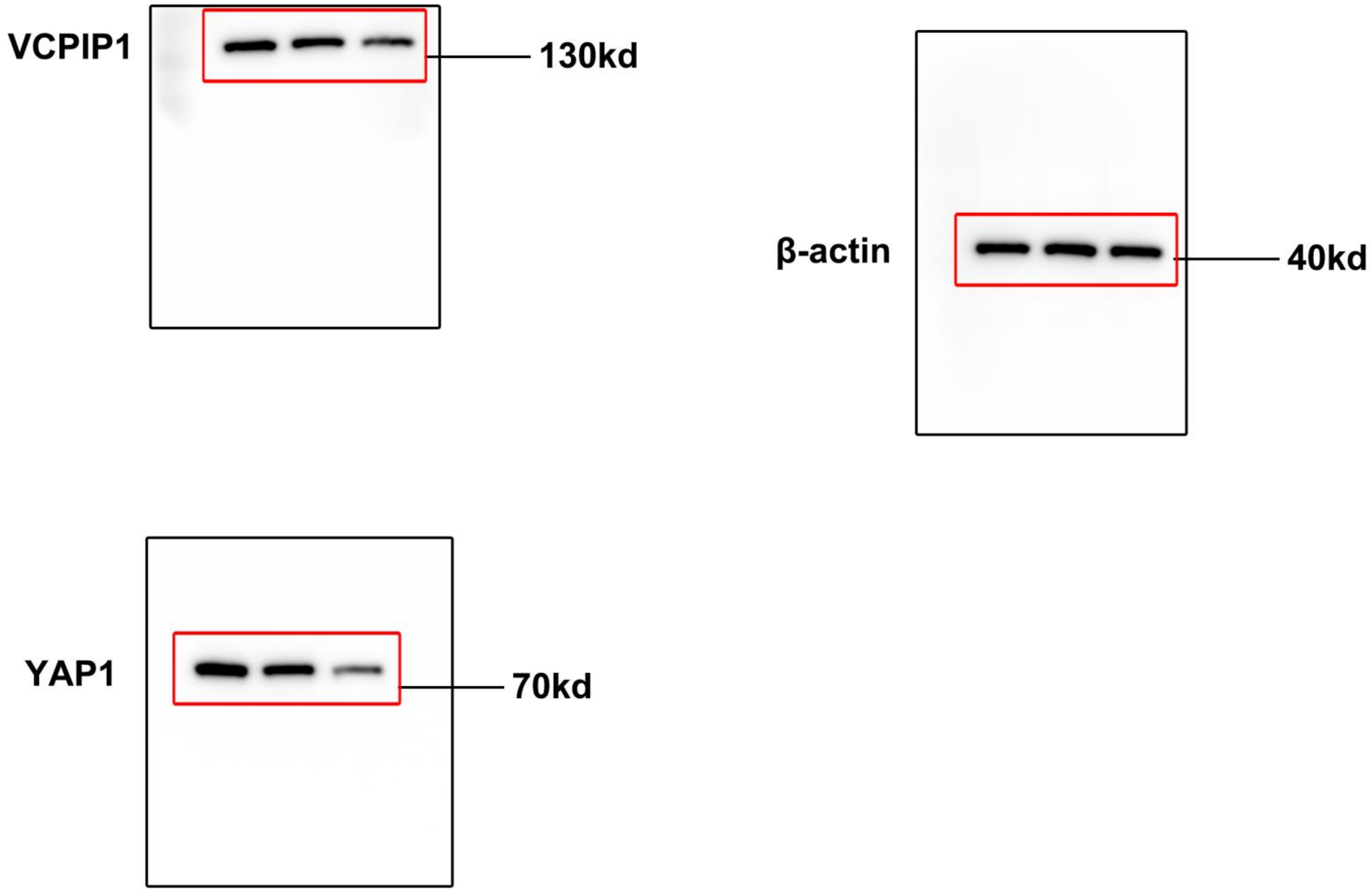

Figure S4B

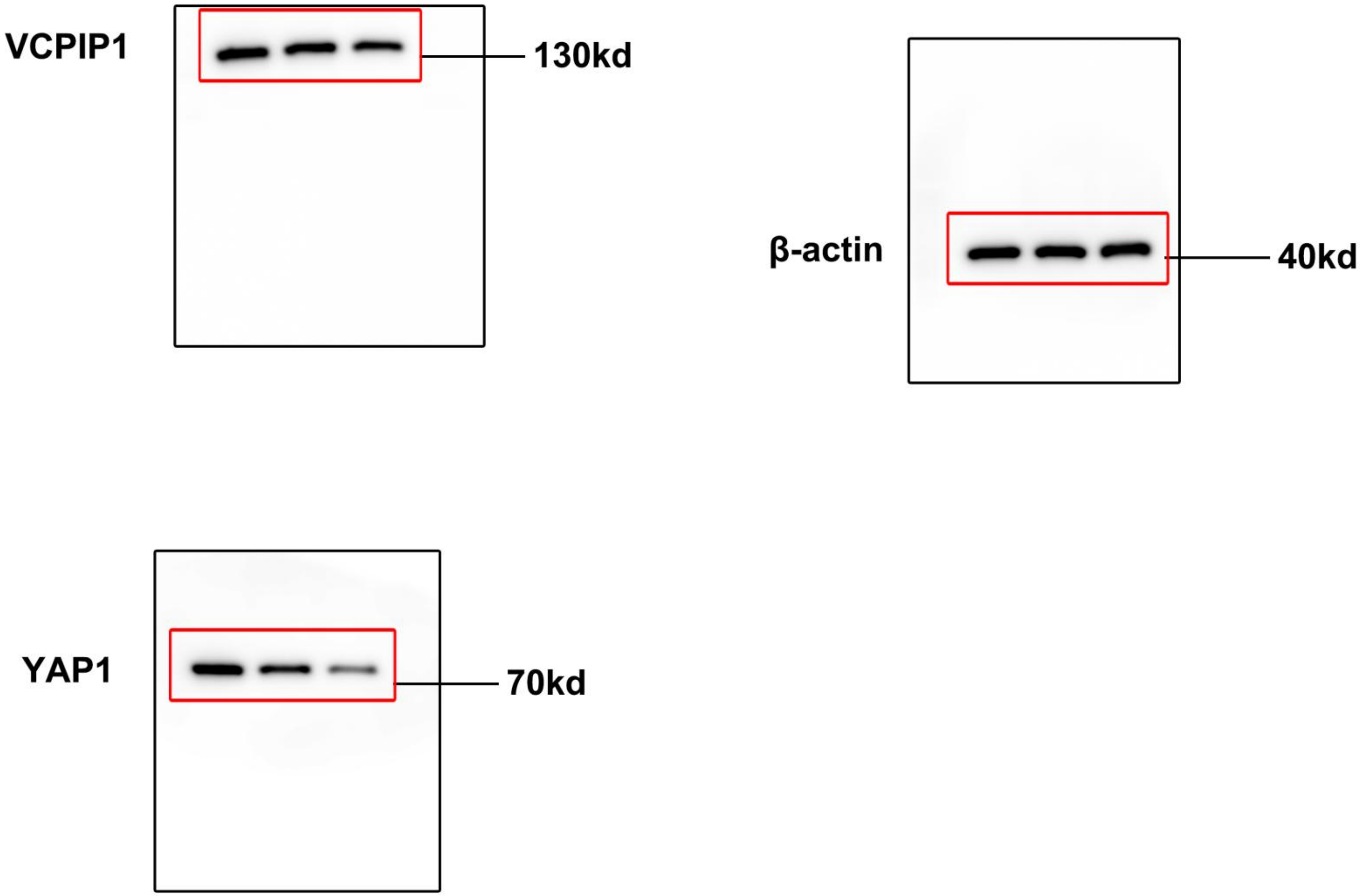

Supplement: Supplementary file 7 — VCPIP1 original data [file 41419_2025_7746_MOESM7_ESM.pdf]
